# 3D*μ*F - Interactive Design Environment for Continuous Flow Microfluidic Devices

**DOI:** 10.1038/s41598-019-45623-z

**Published:** 2019-06-24

**Authors:** Radhakrishna Sanka, Joshua Lippai, Dinithi Samarasekera, Sarah Nemsick, Douglas Densmore

**Affiliations:** 1 Boston University, Department of Electrical and Computer Engineering, Boston, 02215 United States; 2 Boston University, Department of Biomedical Engineering, Boston, 02215 United States; 3 Boston University, Biological Design Center, Boston, 02215 United States

**Keywords:** Biotechnology, Engineering

## Abstract

The design of microfluidic Lab on a Chip (LoC) systems is an onerous task requiring specialized skills in fluid dynamics, mechanical design drafting, and manufacturing. Engineers face significant challenges during the labor-intensive process of designing microfluidic devices, with very few specialized tools that help automate the process. Typical design iterations require the engineer to research the architecture, manually draft the device layout, optimize for manufacturing processes, and manually calculate and program the valve sequences that operate the microfluidic device. The problem compounds when engineers not only have to test the functionality of the chip but are also expected to optimize them for the robust execution of biological assays. In this paper, we present an interactive tool for designing continuous flow microfluidic devices. 3D*μ*F is the first completely open source interactive microfluidic system designer that readily supports state of the art design automation algorithms. Through various case studies, we show 3D*μ*F can be used to reproduce designs from literature, provide metrics for evaluating microfluidic design complexity and showcase how 3D*μ*F is a platform for integrating a wide assortment of engineering techniques used in the design of microfluidic devices as a part of the standard design workflow.

## Introduction

The integration of microfluidics into experimental microbiology is limited to very few specific use cases in both academia and industry despite numerous advances in the technology^1,2^. Even in research areas where microfluidic devices have the potential to address numerous challenges in biological computation, primarily when used as the platform on which synthetic biological systems can be specified, designed, built, and tested^3^, the absence of widespread use of microfluidic devices is symptomatic of the problems faced in engineering and manufacturing them.

Currently, the standard engineering practices within research groups for microfluidics design fall within one of two distinct groups: (1) Component Designers (2) System Designers. Component Designers consist of researchers who engineer new components that take advantage of fluid mechanical phenomena. System Designers consist of researchers who design large scale systems with tens to thousands of geometric features. While many System Designers use Computer Aided Design (CAD) tools like Solidworks and AutoCAD, a sizable portion of researchers use vector design tools like Adobe Illustrator to draw the layouts of large multilayered designs manually. The designer’s need to organize geometries patterned at different depths drives the usage of these tools. For Component Designers, the ability to carefully draft complex parametric geometries and visualize 2D/3D projections of the device is essential. System Designers, on the other hand, value the ability to manage multilayered designs and perform rapid iterations efficiently. Despite being sophisticated software tools, both the vector editors and general purpose CAD tools place the burden of engineering the device for manufacturing and runtime design onto the user.

In electronics, Electronic Design Automation (EDA) tools take on the bulk of the labor-intensive design tasks. Without the availability of these tools, modern-day electronics would not exist. The formalization of the engineering process and the development of EDA tools have, in turn, affected the entire electronics and technology industry resulting in the significant proliferation of electronic devices. Hence, the potential benefits of introducing domain specific Computer Aided Design (CAD) tools have motivated researchers to develop design automation tools for microfluidic engineering. The survey^4^ published by Araci *et al*. provides an excellent overview of the various design automation tools developed in the field.

Additionally, current CAD tools and vector editors do not capture any information about the topology of the microfluidic device. The effects of this loss of information propagate throughout the design flow and deteriorate the researcher’s ability to cycle through design iterations rapidly. In light of the potential to automate microfluidic device designs, McDaniel *et al*. surveyed approximately 100 groups in academia and industry. This study, conducted as a part of an NSF I-Corps program on mVLSI (Microfluidics Very Large Scale Integration) software tools concluded that an interactive design tool which integrates design generation algorithms would be of high value to the microfluidics community^5^.

To that effect, we introduce 3D*μ*F, an interactive design tool that can capture the architecture of the device during the design process. By incorporating the designs into a parametric microfluidic component library, 3D*μ*F enables component designers to share their work with the rest of the community while simultaneously allowing system designers to quickly create large and complex microfluidic designs by reusing existing microfluidic technologies. This method increases standardization and helps mitigate a source of experimental non-reproducibility which plagues both microfluidics and the associated application fields. Various components that mix, distribute, and manipulate fluids, in addition to those intended for performing biological functions such as trapping and incubating cells, are a part of the microfluidic component library implemented by 3D*μ*F. We envision that the microfluidic design automation (MDA) community would be able to extend the platform provided by 3D*μ*F by leveraging the data models and visualization provided by the tool. The designs built in 3D*μ*F would allow researchers to incorporate algorithms that could engineer the devices designed in 3D*μ*F to become more fault-tolerant^6^, have a longer lifetime^7^, support automated testing^8^ and allow for the optimization of the control line layout^9^.

Through a series of case studies that include nine devices from literature and devices that capture microbiological protocols, we demonstrate how the tool can be used to design microfluidic technologies useful for Lab on a Chip (LoC) applications. We then introduce metrics that capture the effort required to design devices and show how 3D*μ*F reduces the amount of effort to create them. We then show a collection of software examples, that demonstrate 3D*μ*F’s potential as an extensible software platform by demonstrating various examples of how data captured by this tool can be used to integrate techniques that Specify, Design, Simulate, Build and Execute microfluidic devices. Through component level standardization and the facilitation of a systematic design process, we believe that 3D*μ*F paves the first step in creating a standardized and automated engineering process for microfluidics similar to what is available to electronics designers.

## Designing with 3D*μ*F

**Figure 1:**
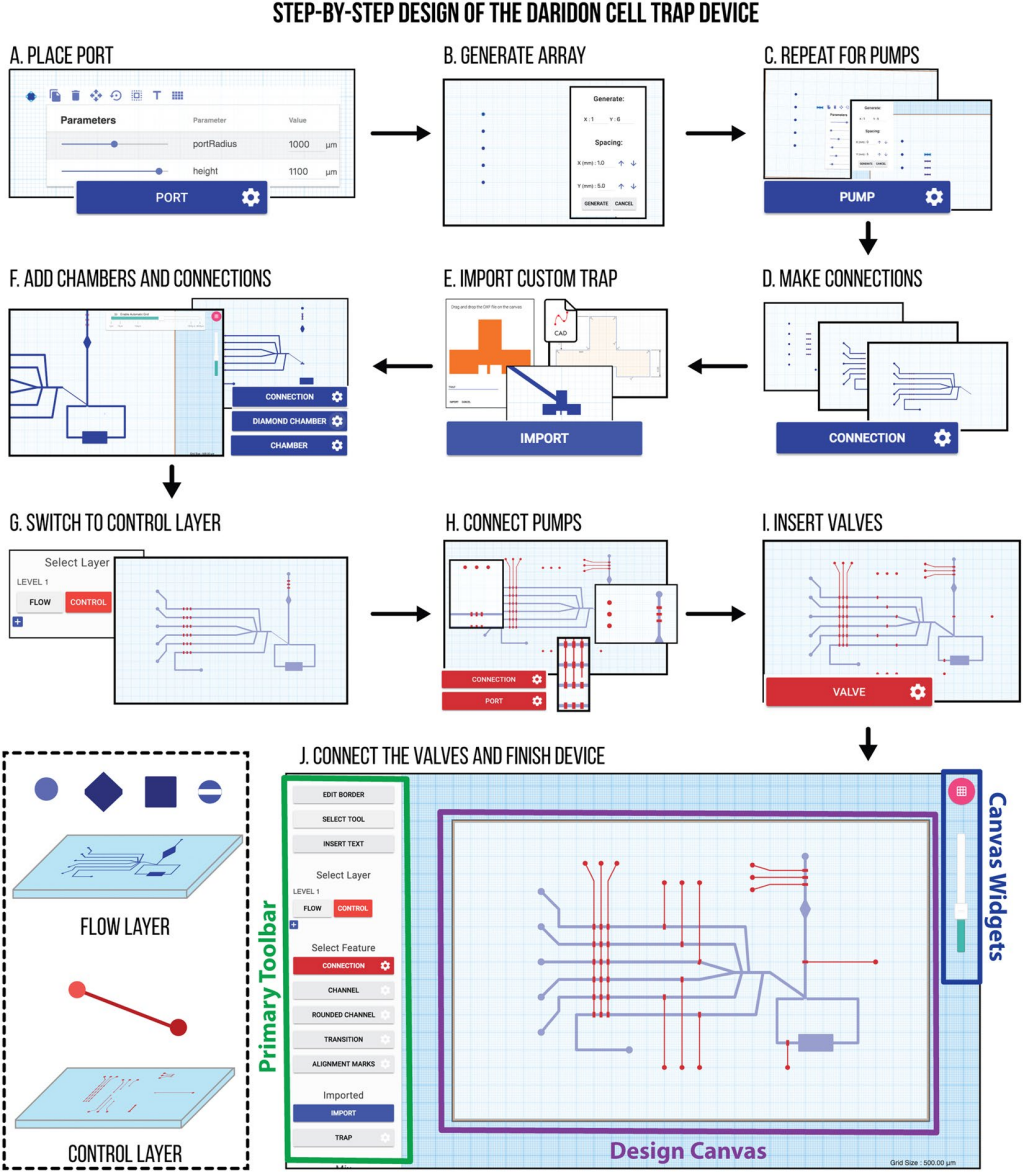
Sample steps to create a microfluidic device using 3D*μ*F. The interface provides the user with a design canvas onto which they can place parametric components, and various tools in the toolbar that allows them to customize the placed components, select whether to place the component in the FLOW or the CONTROL layer and to choose how to export the designs. Users can utilize the design functionality provided by the tool via the component context menus and the primary toolbar as seen above. The video demonstration of this example is available in the SI.

3D*μ*F is a web-based design tool which allows System Designers to engineer their microfluidic designs. Available at http://3duf.org, the design environment is built entirely in Javascript and runs within the browser without the need for any complex cloud infrastructure. When loaded, the tool provides the user with a simple user interface as seen in Fig. 1J. which consists of a Design Canvas where the user can interactively construct their microfluidic design and a Primary Toolbar where they can select the different tools necessary to generate the design.

Figure 1 shows how the Daridon Single Cell Trap^10^ is constructed using 3D*μ*F. In Fig. 1A, the first step illustrates how the user can place components on the canvas by activating its corresponding tool on the primary toolbar Fig. 1J. Since the design requires a series of uniformly spaced PORTs, replicas of this component with the same parameters can be generated using the *Generate Array* tool as seen in step Fig. 1B. We then repeat the same process for the PUMP components to generate an array of actuators that pull liquid through each respective port. In step Fig. 1D, we manually create all connections between the ports and components using the CONNECTION tool (automated routing will be available in future versions based on Hightower *et al*.^11^).

Since the device we intend to replicate employs a custom cell trap^10^, we create a custom component in 3D*μ*F by importing the design from an external CAD tool, as seen in Fig. 1E, in the form of a. *DXF* file. 3D*μ*F then converts the imported geometry into a component object by inferring all the closed shapes described in the. *DXF* file. We complete the FLOW layer design by adding the remaining Reaction Chambers and creating the corresponding interconnections in Fig. 1F. Upon completing the FLOW layer, the user can switch to the CONTROL layer to add Valves (using the VALVE tool) and the corresponding control infrastructure. The tool then automatically aligns the valve and redraws the connection in the case of Valve3D^12^. Finally, the user then creates the corresponding control infrastructure by placing Ports and Connections on the control layer in step Fig. 1G, by repeating the steps A and D. Using 3D*μ*F, this design with 47 components and 103 channel elements was constructed in less than 20 minutes and is available in the Supplementary Information (SI).

## Results

### Parametric Design Engineering with 3D*μ*F

**Figure 2:**
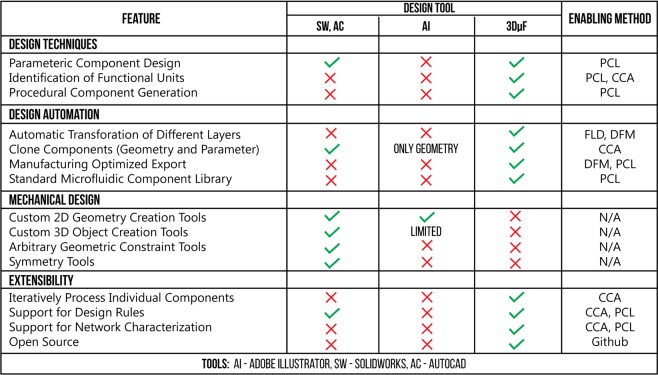
This table compares the functionality of different design tools to design and optimize microfluidic systems. It also includes the concepts that enable 3D*μ*F to provide the user with each of the features referenced in the Methods section: PCL - Parametric Component Library, FLD - Functional Layer Design, CCA - Component-Connection Architecture and DFM - Design for Manufacturing, Github - https://github.com/CIDARLAB/3DuF.

3D*μ*F is a semi-automated design tool that employs design techniques typically seen in electronics CAD tools, such as PCB (printed circuit board) editors, in the design of microfluidic devices. 3D*μ*F automatically captures the relationships between objects placed on the design canvas that would be necessary to understand the underlying architecture of a microfluidic device created within its design environment. While the provided user interface might seem like a simple environment lacking the advanced drafting and transformation features often seen in design tools such as SolidWorks and AutoCAD, the simple user interface abstracts much of the design automation that attempts to obviate the needs of the user to use the features as mentioned previously. The data captured using 3D*μ*F drastically reduces the effort necessary to create larger designs with moderately high complexity. We compare the various features that are uniquely available within 3D*μ*F against other tools typically used by researchers to design microfluidic devices, as seen in Fig. 2. While 3D*μ*F may appear to fall short in *Mechanical Design*, the simple user interface is sufficient for visualizing the 2D profiles of the placed components. 3D*μ*F excels in the areas of *Design Techniques*, *Design Automation* and *Extensibility* as it was designed explicitly for creating large microfluidic devices leveraging the various strategies used by researchers in microfluidic design automation.

**Figure 3:**
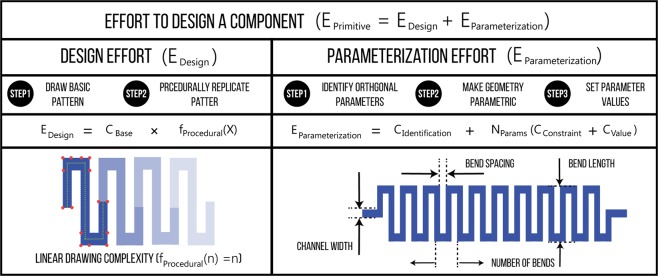
Effort can be quantitatively measured. The figure shows how the effort required create a component *E*_*Primitive*_ is composed of two expressions, Design (*E*_*Design*_) and Parameterize (*E*_*Parameterization*_). Both the Design and Parameterization involve two and three steps respectively and are represented by the different terms (*C*_*Base*_*f*_*Procedural*_(*X*), *C*_*Identification*_, etc.) in the *E*_*Design*_, *E*_*Parameterization*_ expressions. Additional details are available in the SI.

Since the effort required to design microfluidic devices can vary widely in each case, we developed metrics to measure the effort required to create each component in the 3D*μ*F library. We can then compute the effort required to design the full device by computing the total effort required to create all the components. Since the process of designing a component consists of two stages, *Design* and *Parameterization*, the total effort required is expressed as *E*_*Primitive*_ = *E*_*Design*_ + *E*_*Parameterization*_, where Effort E_*_ is measured in *actions*. Figure 3 illustrates how the design and parameterization efforts can be captured as *E*_*Design*_ = *C*_*Base*_*f*_*Procedural*_(*X*) and *E*_*Parameterization*_ = *C*_*Identification*_ + *N*_*Params*_(*C*_*Constraint*_ + *C*_*Value*_) respectively. Using these formulae, the *Effort* required to create a *MIXER* with 8 bends would be 88 *actions* (*C*_*Base*_ = 6, *C*_*Identification*_ = 20, *C*_*Value*_ = 4,*f*_*Procedural*_(*X*) = *X*|*X* = 8). Detailed explanation of each of the terms is available in SI, Section 8.1 and 8.2.

In addition to expanding the automation capabilities of the design, 3D*μ*F helps reduce the debugging effort required to design devices. When designing complex microfluidic systems, one of the most time consuming and error-prone portions of the design drafting process is the parameterization of the various geometries constituting the device design. 3D*μ*F inherently reduces a large amount of the effort that the designer has to undertake in assigning proper parametric geometry constraints.

### Case Study 1 - Microfluidics from Literature

**Figure 4:**
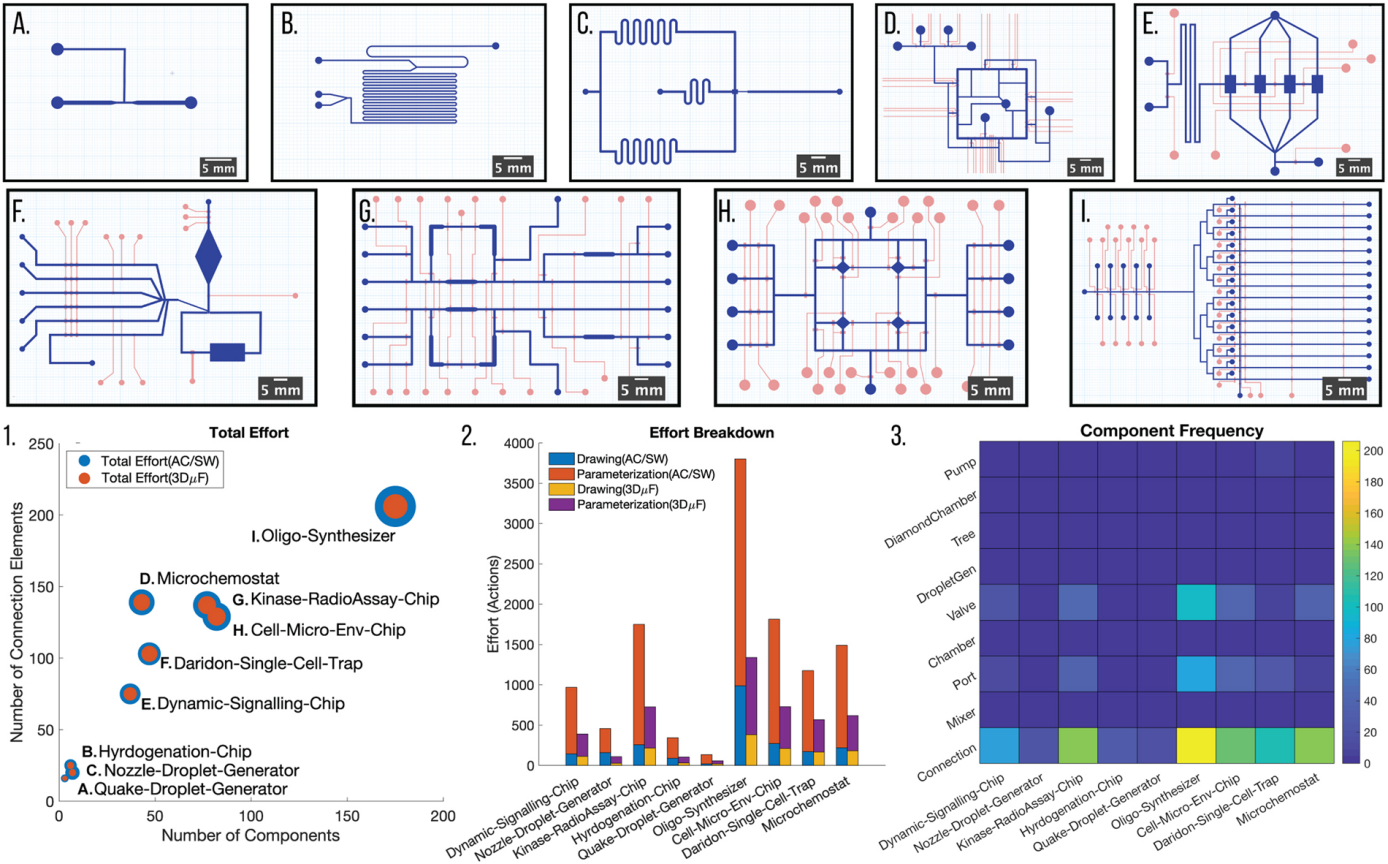
Reproducing Designs from Literature (A-I). Graph 1 characterizes the designs using the number of components and connection elements present in the design. The radius of the markers shows that the total effort (${E}_{Total}=\sum {E}_{Primitive}$) required to draw and parameterize the design using a CAD tool eclipses the effort required to create the design using 3D*μ*F. Graph 2 shows the breakdown of the effort required in Drawing and Parameterization for each of the design with different design tools. It also shows how the parameterization process requires more *E*_*Parameterization*_ since the manual addition of geometric constraints would typically require the user to continuously modify the constraints while incrementally building the design to prevent *overconstraining*. Graph 3 further shows the distribution by which different components compose the designs from literature, and it also shows how that even though the complexity of the design might scale in size, the relative distribution of different kinds of components is similar in all of the designs.

In practice, it is often difficult and sometimes impossible to recreate devices published in literature since the images and written specifications accompanying the manuscript are often insufficient to reproduce the device. Even in some cases, when the CAD designs are given, it is almost impossible to parameterize the design without substantial characterization work. Conventional CAD tools cannot capture the design as a composition of individual objects; they instead store the entire design as a large set of shapes. 3D*μ*F’s capability to generate a structured device described as a network of connected components and connections partially addresses this fundamental problem in engineering communication. Further, since 3D*μ*F encodes all of the designs in a superset of ParchMINT^13^ (an open microfluidic interchange format), anyone can write simple scripts to extract all the necessary design information. The effort estimations seen in Fig. 4 was computed by summing up the individual efforts required for designing each of the components in the design file using a python script(Available in SI, Section 9). In order to show the potential of 3D*μ*F in designing devices with the standard component library, we designed nine microfluidic devices found in literature used for lab automation. The different devices designed as a part of the case study were selected for their popularity(citations) or as their relevance as scientific research platforms^3^. The designs seen in Fig. 4 are: (A) Quake Droplet Generator^14^ [Citations:1917] (B) Kobayashi Hydrogenation Chip^15^ [Citations:561] (C) Nozzle Droplet Generator Chip^16^ [Citations:298] (D) A single microchemostat unit^17^ [Citations:595] (E) Dynamic Signaling Chip^18^ [Citations:76] (F) Daridon Single Cell Trap^10^ [Citations:636] (G) Kinase Radioassay Chip^19^ [Citations:43] (H) Cell-Microenvironment Chip^20^ [Citations:58] (I) Oligonucleotide Synthesis Chip^21^ [Citations:85]. Each of these publications were only accompanied by pictures and limited textual descriptions of some design parameters making it virtually impossible to recreate the device from what is given in the paper. All of the devices in Fig. 4 were fabricated with the intention of demonstrating 3D*μ*F’s capability for capturing the complexity of designs seen in literature and not to create functional prototypes. By fabricating the devices replicated in 3D*μ*F, we were able to show that reasonably complex devices, as seen in literature, can be designed and fabricated using 3D*μ*F. Further Fig. 4 1 and 2 shows how 3D*μ*F reduces the amount of effort required by the system designer to create each of the designs. Additional information is available in the SI, Section 9. The nine designs are available at https://cidarlab.github.io/3DuF-Paper-Designs/.

### Case Study 2 - Modular System Design

**Figure 5:**
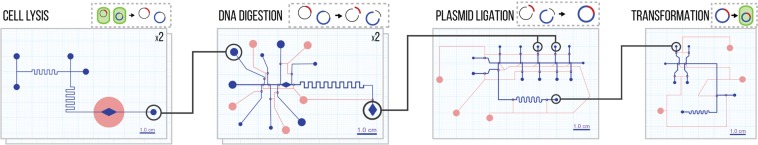
The figure shows the concept of a system that is intended to perform cloning. 3D*μ*F was used to rapidly manufacture and iterate through each of the stages within four months. The system reuses existing 3D*μ*F designs, allowing the user to design and test each subsystem separately before integrating them into a larger system with external purification steps^22^. In the Cell Lysis stage, the plasmids (genetic fragments) are released from cells and captured on magnets. In the DNA digestion stage, the plasmids are digested, using restriction enzymes, into compatible DNA fragments. In the Plasmid Ligation stage, the DNA fragments are ligated together to form a new, complete plasmid. Finally, in the Transformation stage, the new plasmid is transformed into *E. coli* cells for propagation and expression.

When designing large microfluidic systems to perform complex assays, using a modular design approach is advantageous. Each modular subsystem within a library is independently designed and tested to be functional. Therefore when building a complex system, each validated modular subsystem is already functional and “only” requires the user to create connections between them. Figure 5 represents a conceptual microfluidic system designed to perform cloning reactions illustrates an example of a system that performs complex assays. Cloning, a common molecular biology technique used heavily in synthetic biology, often uses restriction enzymes to digest DNA of interest into fragments with compatible ends which undergo ligation. This system, as shown in Fig. 5 reuses four different designs from the MARS repository (http://2017.igem.org/Team:BostonU_HW) which were each individually designed using 3D*μ*F. Because of the component connection abstraction internally used by 3D*μ*F, designers do not encounter problems where the geometric constraints of different geometries become *entangled* requiring them to spend a significant amount of time reassigning the geometric constraints when designs become ‘*overdefined*’(SolidWorks) or ‘*overconstrained*’(AutoCAD). Hence, users can safely import 3D*μ*F designs requiring no additional user effort to prevent changes made to an individual component to propagate through the rest of the design â€” additional information on the four MARS designs is available in the SI, Section 10.

### Platform Extensibility Demonstration

3D*μ*F not only serves as a design tool but also as a framework for the integration of advanced engineering practices into the design workflow. In this case study, we show five examples of such methodologies demonstrated using 3D*μ*F. They span five different stages of the device design lifecycle namely, *Specify*, *Design*, *Simulate*, *Build*, *Execute*. Extended summaries for each the demonstrations and links to the source code are available in the SI, Section 11.

#### Specify - Support for High-Level Device Descriptions

**Figure 6:**
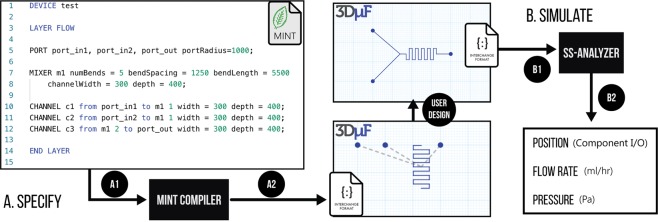
3D*μ*F is not just a design tool. The architectural topology captured by the tool allows 3D*μ*F to be used as a platform for engineering microfluidic devices. A1, A2. (Specify) Shows how the design described in a hardware description language (MINT) can be imported into 3D*μ*F and B1, B2 (Simulate) shows how the design files can be imported into external software that can analyze and estimate flow rates and pressure at different locations on the design.

Hardware Description Languages (HDL) have been critical in synthesizing digital electronic designs. They allow the engineer to specify the functionality of the device using programming language syntax, relying on automation algorithms to generate the actual circuit that performs the functionality. Figure 6A shows how MINT^23^ (a microfluidic HDL) can be compiled and imported into 3D*μ*F. This demonstration shows how a platform like 3D*μ*F can be used to integrate engineering workflows where designs are synthesized from high-level description languages.

#### Design - Support for Design of Experiment Techniques

Typically the design spaces for most microfluidic geometries are large, which can generate a wide range of performance. Researchers often utilize “Design of Experiments” methodologies to either simulate or rapid prototype^24,25^ orthogonal device designs that help them evaluate the performance of the device in the design space. Some of the methods often used in the generation of the orthogonal design of experiments methodologies are the Taguchi Methods^26^. System Designers can take advantage of such experimental methodologies and use 3D*μ*F’s inference of the design to generate the orthogonal array automatically. A video of this demonstration is available in the SI.

#### Simulate - Support for Network Characterization

In order to circumvent the limitations of finite element simulation, researchers have devised strategies that rely on data-mining pre-simulated results for design exploration^27^ and the interaction of particles in fluid flow^28^. By leveraging the component connection abstraction, it is possible to apply techniques used in electronic circuit simulation^29^ on the microfluidic network by deriving electrical circuit equivalent models^30^. In order to illustrate how designs created using the Component-Connection Abstraction can be simulated, we demonstrate a simple tool that converts the design created in 3D*μ*F into an equivalent electrical model to calculate the Pressure and Flow Rates at various points of interest as seen in Fig. 6.

#### Build - Support for Fabrication

The current release of 3D*μ*F has built-in support for generating design files for CNC micro-milling, utilizing the 2.5D Manufacturing Layer Generation technique. Similarly, the 2.5D Manufacturing Layer Generation algorithm could be adapted to generate design files targeting other popular manufacturing processes like Soft Lithography^31^.

#### Execute - Support for Valve Control Programmability

A significant factor that inhibits the transfer of designs is the non-standard electronic infrastructure used to run microfluidic devices. By creating designs in 3D*μ*F, designers can reuse the component names used in the design while describing the behavior in the form of valve actuation sequences(standardized). Additionally, the component-connection graph generated by 3D*μ*F would allow researchers to design algorithms that can synthesize the valve sequences necessary to control the fluid flow when integrating with high-level languages such as BioStream^32^.

## Discussion

3D*μ*F’s capabilities as an open-source, extensible design editor enabled usage highlighted by Case Studies 1 and 2. In particular, the ability of the component library to be used to design the existing literature-grade microfluidic device was demonstrated by Case Study 1, covering a range of widely used device designs. 3D*μ*F, as seen in Fig. 4 additionally reduces the effort to design devices by half. However, 3D*μ*F has the potential to reduce the effort even further with its ability to allow for different steps in the design process to be automated. 3D*μ*F’s use of standardization at multiple levels enables its interactions with existing microfluidic design tools and methodologies. Without any additional effort, each design in Case Study 1 could be imported into tools that integrate algorithmic advances in MDA^6,7,9,33^.

Though 3D*μ*F supports the import of components using DXF, the size of the component library is a significant drawback with 3D*μ*F since the geometries imported from DXF files lack the metadata required for fully integrating the design with any of its platform capabilities and since the user cannot create custom components using 3D*μ*F. However, as the LoC community using 3D*μ*F grows, the potential complexity of chips made by integrating existing work would only increase with every contribution. Every addition to the component library would then be able to leverage 3D*μ*F’s capabilities and allow for large-scale standardization of microfluidic device design and their corresponding operation and manufacturing infrastructure.

While Case Study 2 highlights 3D*μ*F’s usefulness in enabling larger microfluidic system designs, it could take substantial effort to modify such a design based on changes to a single component between design iterations and different operating conditions. Future work could automate this process by incorporating algorithms that synthesize designs for these different scenarios. 3D*μ*F could then be used to resize designs leveraging the underlying component-connection architecture. However, most of these parameters and rules for the synthesis of a microfluidic system would be context-, application-, flowrate-specific. Hence, we believe that the research community would leverage 3D*μ*F and create the necessary extensions.

## Methods

### Parametric Component Library

**Figure 7:**
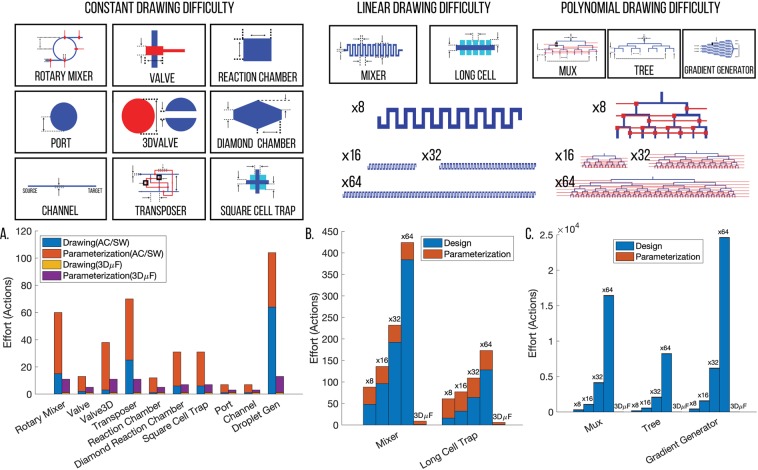
The parametric component library significantly reduces the effort required to draw different microfluidic geometries. Graph A compares the effort taken to design microfluidic geometries using CAD design tools vs. the effort required to design using 3D*μ*F. Graphs B and C show how the effort required to design the procedurally generated geometries scales with the number of outputs when created using CAD tools and how the effort required is nearly negligible when designing using 3D*μ*F. The Procedure and Calculations for computing the *Effort* for all the components is available in the SI, Section 8.

The components that are a part of the component library are all procedurally generated by 3D*μ*F. The components not only abstract microfluidic functionality but also lay the foundation to standardize the engineering flow. Figure 7 shows how using 3D*μ*F can drastically reduce the effort required to design and parameterize different geometries. Figure 7 also shows how the benefits of having procedurally generated component libraries can drastically reduce the effort involved in generating components like MIXER and MUX which require effort that scales by linear and polynomial difficulty. *Effort*, a heuristic metric used throughout the paper (measured in actions) attempts to naively capture the number of actions required by the user in different design tools to draw and parameterize for creating each component as seen in the SI, Section 8.

### Component Connection Architecture

The component connection architecture used by 3D*μ*F to model the microfluidic design is the core enabling methodology that allows the tool to set itself apart from all the other available tools. It enables the designer to capture the architecture underlying the geometries that are drawn on the canvas and is the foundation for most of the 3D*μ*F’s functionality. Rather than drawing polygons, manually setting geometric constraints, the component connection architecture allows the user to place predefined geometries in the form of components directly and then connect them using connections. Components allow the complexity of fluid mechanics to be abstracted, allowing engineers with little or no experience in the specific area of fluid mechanics to implement a diverse range of microfluidic functionality quickly.

### Functional Layer Design

Figure 1 shows how a microfluidic device’s design abstracted into components and connections belonging to different layers, the Flow *LAYER* which consists of geometries that perform the primary operation of the microfluidic device and the Control *LAYER* which consists of the geometries that control the movements of the fluids in its corresponding Flow *LAYER*. 3D*μ*F allows for multilayered designs in a manner that replicates the visualization in AutoCAD and Illustrator. For designs that have more than a single Flow and Control layer, 3D*μ*F allows for the creation of multi-leveled designs that can be easily added/deleted using the ‘+’ and ‘delete’ buttons seen in Fig. 1H. 3D*μ*F automatically creates a pair of corresponding Flow and Control layers for every new level that is added to design. The tool additionally updates the internal data model to store the relationship between the channels and the valves used in automatic control sequence generation algorithms^34^ as key-value pairs.

### Design for Manufacturing

3D*μ*F allows for the components placed in a single functional layer to have multiple heights. Typically during the fabrication, the designer would have to manually keep track of the features belonging to different depths (layers when using Adobe Illustrator or using a hierarchical coloring system when using AutoCAD^35^). 3D*μ*F internally assigns each of the geometries a DFM (Design for Manufacturing) class, namely *XY*, *XYZ*, *Z* and *EDGE* that indicating the fabrication complexity of the geometry. By grouping all of the features with different heights in the *XY* class, 3D*μ*F obviates the need for the user to follow any of the cumbersome schemes described earlier. In practice, the user would specify the ‘height’ parameter for each of the components placed and let 3D*μ*F handle the process of generating the different “Manufacturing Layers”. The combination of the “DFM Classes”, “Manufacturing Layers” and “Functional Layers” captures a majority of the complexity faced when designing mLSI designs and allows for the fabrication support to be extended for different kinds of fabrication processes. Details of the DFM classes, manufacturing layer generation, and the fabrication protocol are available in the SI.

## Conclusion

The design of microfluidic devices for the biological sciences is currently limited for numerous reasons. These include the lack of domain-specific computer-aided design environments. This paper presents a lightweight, web-based, open source tool (3D*μ*F) that addresses many of the current software shortcomings while providing several innovative features. We illustrate both how existing, popular microfluidics designs can be created as well as how to quantify the effort associated with creating microfluidic designs in-silico. Our approach builds on techniques used in the EDA community and provides a solid foundation that can be expanded on by individual, motivated software engineers. We have provided numerous design examples, videos, and demonstration software that complement the design tool its capabilities.

In electronics, the availability of free PCB design tools such as KiCAD (kicad-pcb.org/) (open source) and Eagle(autodesk.com/products/eagle/) helped reduce the barrier of entry and push the envelope of what can be designed by individuals, thus increasing new market opportunities. Similarly, 3D*μ*F is poised to play an essential role in the growing microfluidic design ecosystem that includes Metafluidics^36^, AutoPAD^37^ and other approaches to democratize microfluidics^38,39^.

## Supplementary information

Supplementary Information

Primitives Effort Workbook

SS-Analyzer Demonstration

Design of Experiments using Taguchi Methods Demonstration

Design of Daridon Cell Trap - 8x Normal Speed
