# Supplementary material for: 3DμF - Interactive Design Environment for Continuous Flow Microfluidic Devices
Source: Sci Rep. 2019 Jun 24;9:9166. doi: 10.1038/s41598-019-45623-z (PMC6591506; doi:10.1038/s41598-019-45623-z)
Supplement: Supplementary file 1 — Supplementary Information [file 41598_2019_45623_MOESM1_ESM.pdf]

3DμF- Interactive Design Environment for Microfluidic Devices

Radhakrishna Sanka, Joshua Lippai, Dinithi Samarasekera,
Sarah Nemsick and Douglas Densmore

May 30, 2019

Supplementary Material

Contents

1 Software 5

2 Parametric Component Library 6

3 Design Environment 9
- 3.1 Design Canvas . . . 10
- 3.2 Primary Toolbar . . . 10
- 3.3 Context Menu . . . 11
- 3.4 Tool Windows . . . 11

4 Semi-Automated Design Features 12
- 4.1 Design Primitives . . . 12
- 4.2 Parametric Design . . . 12

5 Mechanical Design Features 13
- 5.1 Position and Pattern Tools . . . 13
- 5.2 DXF Support . . . 13
  - 5.2.1 DXF Import Examples . . . 13
  - 5.2.2 Import Modes . . . 14

6 Extending the Component Library 16
- 6.1 Creating the Definition . . . 16
- 6.2 Registering the Definition . . . 19
- 6.3 Create UI Element . . . 19
- 6.4 Registering the Event Handlers . . . 20
  - 6.4.1 Query Selectors . . . 20
  - 6.4.2 Event Handlers . . . 20
  - 6.4.3 Tool Association . . . 21

7 Interchange Format 22
- 7.1 Overview . . . 22
- 7.2 Feature Object . . . 23

8 Parametric Design Engineering 24
- 8.1 Effort Metrics . . . 24
  - 8.1.1 Design Effort . . . 25
  - 8.1.2 Parameterization Effort . . . 25
  - 8.1.3 Constants and Assumptions . . . 26
- 8.2 Design Effort of a MIXER . . . 29
  - 8.2.1 Design Effort (E_{Design}) . . . 29
  - 8.2.2 Parameterization Effort ($E_{Parameterization}$) . . . 29

9 Case Study 1 - Devices from Literature **30**
- 9.1 Device Descriptions . . . 31
- 9.2 Milled Devices . . . 34
- 9.3 Designs Website . . . 41
- 9.4 Effort Calculation Script . . . 41

10 Case Study 2 - Modular System Design **42**
- 10.1 Discussion . . . 42
- 10.2 Transformation . . . 44
  - 10.2.1 Chip Design . . . 44
  - 10.2.2 Design Layers . . . 45
  - 10.2.3 Inputs and Outputs . . . 46
  - 10.2.4 Protocol . . . 47
- 10.3 Ligation . . . 51
  - 10.3.1 Chip Design . . . 51
  - 10.3.2 Design Layers . . . 52
  - 10.3.3 Inputs and Outputs . . . 52
  - 10.3.4 Protocol . . . 53
- 10.4 DNA Digest . . . 57
  - 10.4.1 Chip Design . . . 57
  - 10.4.2 Design Layers . . . 58
  - 10.4.3 Inputs and Outputs . . . 58
  - 10.4.4 Protocol . . . 59
- 10.5 Cell Lysis . . . 62
  - 10.5.1 Chip Design . . . 62
  - 10.5.2 Design Layers . . . 63
  - 10.5.3 Inputs and Outputs . . . 64
  - 10.5.4 Protocol . . . 64

11 Platform Extensibility Demonstrations **67**
- 11.1 Overview . . . 67
- 11.2 Specify - Support for High-Level Device Descriptions . . . 67
  - 11.2.1 Explanation of MINT Syntax . . . 68
  - 11.2.2 Demonstration . . . 69
  - 11.2.3 Resources . . . 70
- 11.3 Design - Support for Design of Experiments . . . 71
  - 11.3.1 Example - DOE to improve Mixer Efficiency . . . 71
  - 11.3.2 Generate Orthogonal Array . . . 72
  - 11.3.3 Demonstration . . . 72
  - 11.3.4 Resources . . . 73
- 11.4 Simulate - Support for Network Characterization . . . 74

11.4.1 Demonstration 74
11.4.2 Resources 74
11.5 Build - Support for Fabrication 75
11.5.1 Design For Manufacturing Classes 75
11.5.2 Manufacturing Output Generation 75
11.5.3 Demonstration 76
11.5.4 Resources 76
11.6 Execute - Support for Valve Control Programability 77
11.6.1 Demonstration 78
11.6.2 Sequential Command Examples 78
11.6.3 Resources 78

12 Makerfluidics Protocol - SVG Manipulation **79**
12.1 Preparing the SVG 79
12.2 Tool Settings 80
12.3 Tool-path Generation 80

1 Software

3DμF is entirely built in Javascript, enabling the software to be run entirely on the browser without the need for an internet connection. The core libraries that powers 3DμF are paper.js [1] to help it render the design canvas and generate SVG files, and dxf-parser to help the import of DXF files generated by other tools. Since the Javascript language is not strongly typed, the 3DμF source code takes advantage of ES6 syntax to allow for more developer friendly code organization.

Functionality	Library
Visualization and SVG Geometries	paperjs
	http://paperjs.org/
DXF Deserialization	dxf-parser
	https://github.com/gdsestimating/dxf-parser
DXF Shape Generation	graphlib
	https://github.com/dagrejs/graphlib
Zip File Generation	jszip
	https://www.npmjs.com/package/jszip
Zoombar UI	nouislider, wnumb
	https://refreshless.com/nouislider/<br>https://www.npmjs.com/package/wnumb

Table 1: List of libraries used in 3DμF that are necessary

Since 3DμF is a large project intended to be extendable by the community, the entire project was written using ES6 syntax to allow for more developer friendly code syntaxes, object-oriented programming primitives, block scope identifiers, and a simplified import system. The ES6 Javascript needs to be transpiled into standard Javascript that can be run by the browser. This is done using the babel compiler https://babeljs.io/. All of the development infrastructure required

[1] https://paperjs.org

2 Parametric Component Library

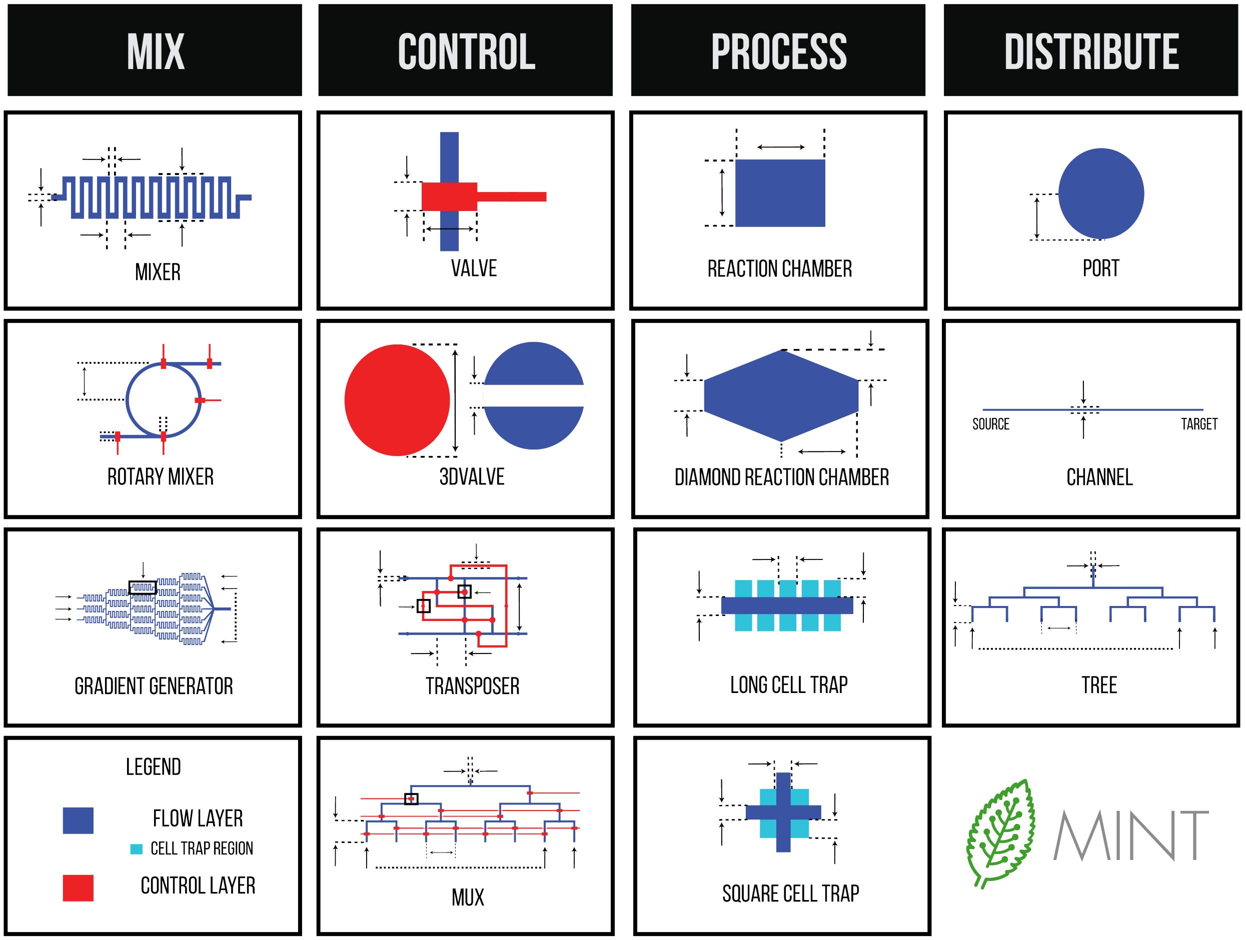

Figure 1: This figure shows the list of parametric components that are part of the MINT library. Their function broadly classifies the components: Mix, Distribute, Control and Process. The figure further illustrates the geometric features that are parametric in the component specification. Components such as the *TRANSPOSER* and *MUX* are composed of other components and include the parameters used by the individual component. These components are shown to be enclosed by the rectangular boxes.

In addition to streamlining the design flow, a component based abstraction would allow for refinement of fabrication processes involved with creating each of the components with various manufacturing technologies[14]. 3DµF uses a parametric component library where boolean and numeric parameters define the component geometry. The parametric component library was first introduced in 'Fluigi'[8], a microfluidic design automation tool, and was expanded to include additional microfluidic components from scientific and microfluidic literature. It was further extended to include the syntax and semantics to create a self-contained Microfluidic Hardware Description Language called MINT for describing microfluidics[7].

3DμF borrows the design paradigm introduced in MINT along with a subset of components. These components are broadly categorized into Mix, Distribute, Control and Process as described below and seen in Figure 1.

Mix: One of the most fundamental fluid operations in microfluidics is mixing. In order to effectively mix multiple fluids, various types of mixing components are a part of the MINT library. They consist of the *MIXER*[22], *GRADIENT GENERATOR*[4] and *ROTARY PUMP*[26].

Distribute: Components such as *CHANNEL*, *TREE* and *NET* are included as a part of the MINT library to allow fluids to be transported to various parts of the microfluidic chip. The *PORT* primitive is provided to allow for fluids to be transported into and out of the chip.

Control: mLSI chips require the presence of a large number of valves and constructs which allow for complex routing of fluids throughout the chip. The components include *MUX*[24], *VALVE*[24], *TRANSPOSER*[21] and *VALVE3D*[6].

Process: The components *LONG CELL TRAP*[18], *SQUARE CELL TRAP*[3], *T DROPLET GENERATOR*[23], *FLOW FOCUS DROPLET GENERATOR*[23], *DIAMOND REACTION CHAMBER* and *REACTION CHAMBER* perform the device's primary functions such as trapping cells, generating droplets and storing biological reagents and liquids on the chip.

3 Design Environment

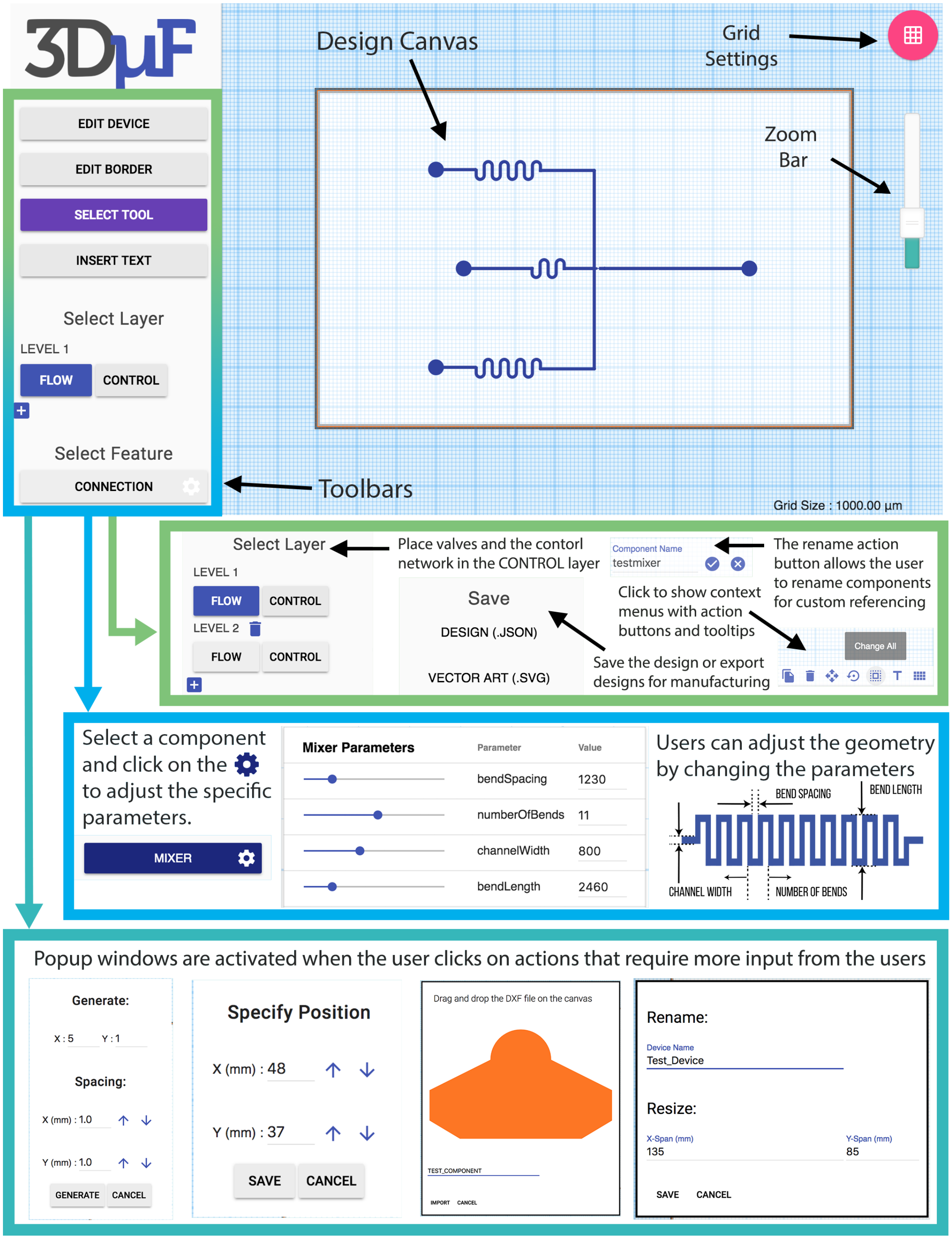

Figure 2: 3DμF's interface provides the user with a design canvas on which they can place parametric components, and various tools in the toolbar which allow the user to customize the placed components, select whether to place the component on the FLOW or the CONTROL layer and choose how to export the designs.

When the user opens 3DμF, they are presented with a design environment as seen in Figure 2. The design environment consists of four primary elements. Namely, the design canvas, the primary toolbar hovering on the canvas, the context menu and the windows. Each of these elements provides the user with the functionality that allows them to design microfluidic devices. To design a device using 3DμF, the user first selects the layer on which they would like to work by clicking on the respective button in the layer selection panel as seen in Figure 2, allowing them to choose between the various *FLOW* and *CONTROL* layers. Users typically start with the *FLOW* layer to make the functional design and then proceed to designing on the corresponding *CONTROL* layer to incorporate valves into the design.

3.1 Design Canvas

The primary UX (user experience) that is central to 3DμF is driven by the large design canvas that takes up the majority of the screen as seen in Figure 2. The design canvas gives 2D visualizations of the microfluidic designs constructed and illustrates how they would look when fabricated. 3DμF implements elements of a PCB design editor by displaying the borders of the device and a grid onto which placements snap automatically. In order to allow the user to easily edit the device and assist the snapping at various zoom levels, the design canvas has a built-in adaptive grid. The adaptive grid modifies the grid's spacing to $5\mu m$, $50\mu m$, $100\mu m$, $500\mu m$, $1000\mu m$ based on the user's zoom level which can be verified using the label in the bottom right corner of the canvas as seen Figure 2. The user can modify the grid spacing and enable/disable the adaptive grid by using the toolbar that is enabled by clicking the Grid Widget on the top right corner of the design canvas as seen in Figure 2. Additionally, the user can change the zoom level of the canvas by using the zoom slider as seen in Figure 2.

3.2 Primary Toolbar

3DμF provides all the microfluidic design functionality available to the user in the form of tool buttons in the Primary Toolbar. This design choice was implemented to help enable easy access to the essential tools in order to lower the learning curve associated with the tool. The toolbar consists of buttons that allow the user to place different microfluidic components[2] and feature onto the design canvas. Each of the placement tool buttons has a 'gear' icon which when clicked opens up the parameters window to allow the user to adjust the properties of the component that will be placed onto the design canvas as seen in Figure 2. Additionally, the toolbar provides the user with options that help them edit the device properties, import components, make connections, switch design layers and save the design as both the JSON file or as a Vector Design file (SVG) respectively used in 3DμF or manufacturing.

[2] The collection of tools can be extended trivially and will be expanded upon user requests and the extension of the MINT component library. Users can fork the 3DμF repository on Github and extend the tool collection on their own.

3.3 Context Menu

Once placed, the user can replicate, modify and delete elements of the microfluidic design on the canvas by selecting (using the Select Tool) and then right-clicking on the selected component to show the context menu as seen in Figure 2. While the context menu typically allows the user to change the parameters of the component, the component menus, however, have additional functionality embedded into them in the form of context menu buttons present in the first row of the menu. Clicking each of the buttons allows the user to *Copy*, *Delete*, *Move*, *Revert Parameters*, *Change All*, *Rename* or *Generate GRID/BANK* of the selected component.

3.4 Tool Windows

While a majority of the user interactions are quite straightforward and require no additional user input, in order to accommodate additional user inputs for some of the actions, the context menu buttons and some of the primary toolbar buttons generate additional tool windows that allow the user to give additional input to the tool as seen in Figure 2.

4 Semi-Automated Design Features

Being the first tool (to the best of our knowledge) combining the elements of both schematic editors from EDA and mechanical engineering CAD tools, the design environment looks like a WYSIWYG (what you see is what you get) user interface with specialized tools that help enable the user to construct the device using specialized primitives that allow for design automation. The strength of 3DµF in comparison to other design tools is when it comes to the ability to understand and construct a logical model of the device internally while simultaneously providing the user with a simple interactive user interface to construct the design.

4.1 Design Primitives

Primitives are the basic building blocks used by design automation tools to abstract the functional/logical units of the design. The user then constructs the device as a composition of primitives. 3DµF, like any other design tools takes advantage of various primitives that abstract the device design. The primitives are namely, *LAYER*, *COMPONENT*, *CONNECTION* and *FEATURE*. 3DuF internally models the design as a collection of *COMPONENTS* linked together with *CONNECTIONS*. It then understands the function of these primitives based on the *LAYER* they belong to. 3DµF models all geometrical artifacts inserted into the design as *FEATURE*s in order to accommodate variations made to the logical design model. By retaining a separate data-structure for representing geometries in the design, 3DµF allows researchers to extend the design models for engineering microfluidics beyond the *COMPONENT* and *CONNECTION* abstraction.

4.2 Parametric Design

One of the most fundamentally important features of 3DµF is the ability to parametrically design the microfluidic design by encoding the definitions of the library components based on the parametric dimensions of its principal geometries[7]. 3DµF enables the user to adjust the parameters of *COMPONENTS*, *CONNECTIONS* and *FEATURES*. It allows users to revert the component parameters to the inbuilt defaults and propagate the parameters to all the other components of the same type as seen in Figure 2 via their respective context menus.

5 Mechanical Design Features

3DµF in addition to giving schematic editor-like functionality to the user, also has some built-in features that help microfluidics designers fine tune the physical characteristics of the device itself. These features can be used to help the device match different interface requirements used by various research groups in industry and academia.

5.1 Position and Pattern Tools

The *Position* and *Generate GRID/BANK* tools enable the user to adjust the position of the components placed on the device canvas and generate 1D and 2D arrays (BANK, GRID) of the component with predefined spacing information. The positioning tool allows the user to match the position of components and ports to industry-wide standards such as the 96 well plate formats[3], or for custom input/output interfacing standards used by different instruments.

5.2 DXF Support

The Component and Border import features in 3DµF allow for an engineering process that allows for the integration of the non-standardized components and mounting equipment. Engineers can simultaneously work on their favorite CAD tools like AutoCAD or SolidWorks to design complex geometries and integrate those components into more extensive and complex device architectures.

5.2.1 DXF Import Examples

Since the DXF designs generated by different tools have variations in coordinate tolerances, DXF primitive utilization. 3DµF's support for DXF files is a work in progress. Figure 3 are some of the examples DXF files that were tested with 3DµF.

Supported DXF Primitives:

- **LINE**
- **POLYLINE**
- **ARC**
- **ELLIPSE**
- **CIRCLE**
- **LWPOLYLINE**

[3] http://www.slas.org/resources/information/industry-standards/

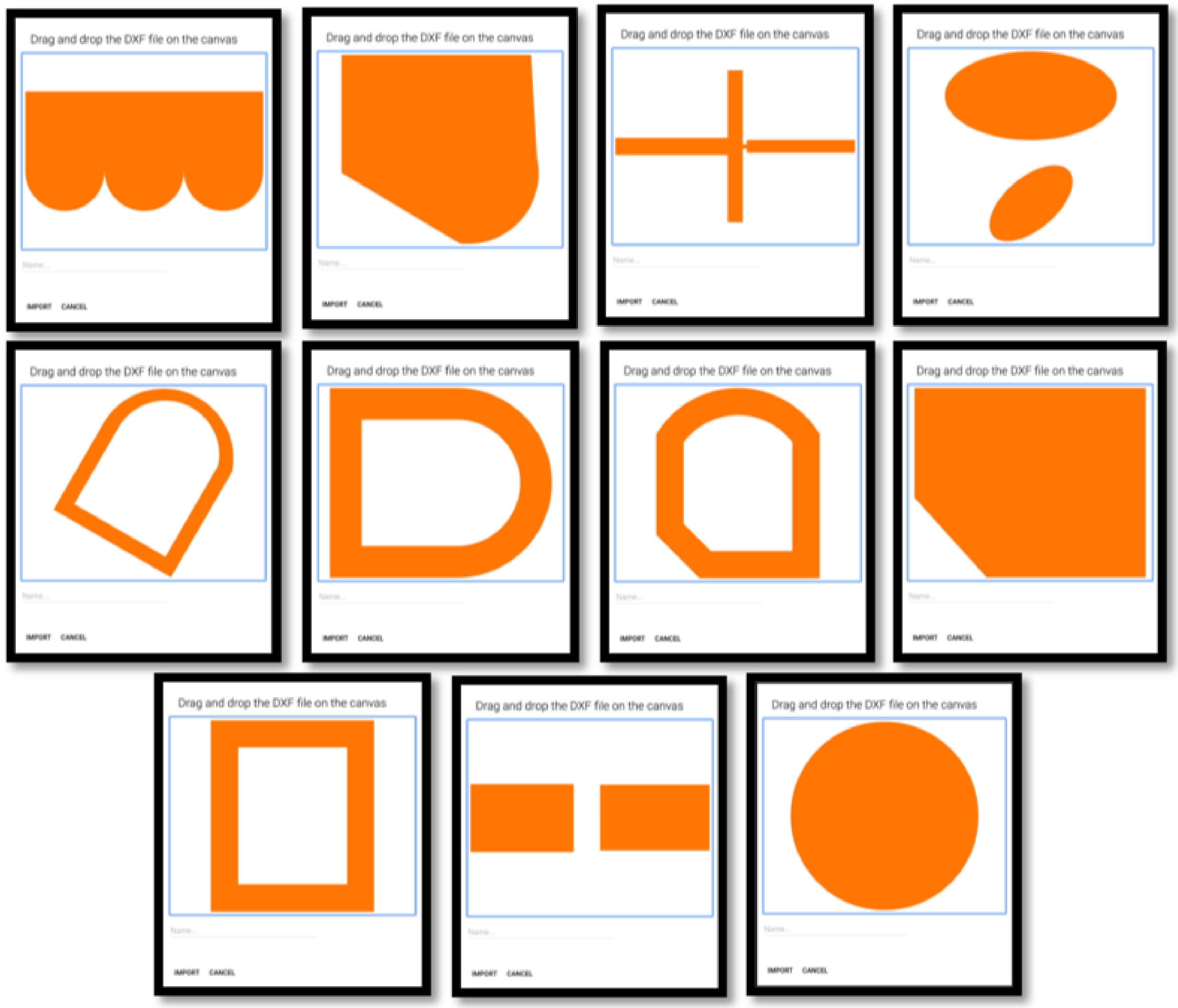

Figure 3: Visualization of DXF Files Imported into 3DµF

5.2.2 Import Modes

3DµF supports the import of DXF files in two modes, *outline* and *filled*. 3DµF uses the DXF importer in the *outline* mode when the user imports border designs. In this mode, 3DµF translates the DXF geometries into SVG geometries with no fill properties.

When the user imports a custom component, 3DµF imports the geometry in the *filled* mode. 3DµF parses through the DXF data and creates a graph $G = (V, E)$ where $V = Geometry\,Vertices$ and where $E = Connectivity\,between\,Geometries$ is computed by computing exhaustive searching for overlap or juxtaposition to a tolerance of $1nm$. Since each shape in the geometry would form a component in a disconnected graph, each of the disconnected components is processed individually to recreate the geometry defined in the DXF file.

3DµF creates a *CompundPath* (defined in the *paper.js* drawing library) for each compo-

nent and creates a continuous geometry by traversing the Geometry Graph G utilizing the geometry information stored in each of the vertices V. 3DµF then drawing library's ability to automatically generate solid fills for overlapping compound paths to generate the filled geometry rendering that would be used to manufacture the component. The implementation can be found in the source code files: */src/app/geometry/geometryEdge.js*, */src/app/geometry/geometryGraph.js* and */src/app/view/render2D/dxfSolidObjectRenderer2D.js*.

6 Extending the Component Library

In version 1.1 of 3DμF, the addition of new components to component library requires the user to perform the following steps:

1. Create the definition of the component in the 3DμF code (Adding a ¡component¿.js file to /src/app/library/).
2. Register the rendering definitions (Modifying /src/app/view/render2D/featureRenderer2D.js).
3. Create UI Elements in the front-end (Modifying index.html).
4. Register the event handlers for the UI Elements with the Placement Tool (Modifying /src/app/view/ui/componentToobar.js and /src/app/viewManager.js).

6.1 Creating the Definition

Create the */src/app/library/newComponent.js* file containing the definition.

```
import Template from "./template";
import paper from "paper";

export  default class NewComponent extends Template{
    constructor(){
        super();
    }

    __setupDefinitions() {
        ...
    }

    render2D(params, key) {
        ...
    }

    render2DTarget(key, params){
        ...
    }
}
```

Populate the definition fields required to by overriding the *__setupDefinitions()* method. More information on the definitions and the different types of definitions are available at https://github.com/CIDARLAB/3DuF/wiki/Adding-new-components-v2

```
__setupDefinitions() {

    /*
    Describes the parameters the positioning too needs to capture
    */
    this.__unique = {
        "<Parameter Name>" : "Point | Segment | String | Point
        | Integer | Float | Boolean",
        ...
    };

    /*
    Describes the parameters that define the component/feature
    */
    this.__heritable = {
        "<Parameter Name>" : "Point | Segment | String | Point
        | Integer | Float | Boolean",
        ...
    };

    /*
    Default Values
    */
    this.__defaults = {
        "<Parameter Name>" : "<Value>",
        ...
    };

    /*
    Display string used for the units
    */
    this.__units = {
        "<Parameter Name>" : "<HTML Display String>",
        ...
    };

    /*
    Minimum value for slider in the parameter window
    */
    this.__minimum = {
```

```
             "<Parameter Name>" : "<Value>",
            ...
        };

        /*
        Maximum value for slider in the parameter window
        */
        this.__maximum = {
             "<Parameter Name>" : "<Value>",
            ...
        };

        /*
        Tool to activate when creating the feature/component
        */
        this.__placementTool = "Existing Tools | Custom Tool";

        /*
        Parameters captured by the tool
        */
        this.__toolParams = {
            "<Parameter Name>" : "<Value>",
            ...
        };

        this.__featureParams = {
            "<Drawing Code Variable Name>" : "<Parameter Name>"
            ...
        };

        this.__targetParams = {
            "<Drawing Code Variable Name>" : "<Parameter Name>"
            ...
        }


    }
```

Finally, the user needs to create the drawing code for allowing 3DµF to render the geometries. *render2D()* is used for generating the device geometry that is displayed and is used for fabrication and *render2DTarget()* is used for generating the translucent geometry

that trails the mouse pointer when the component placement is initiated, describing the target. The drawing utilizes the library paperjs (https://paperjs.org)

6.2 Registering the Definition

In order to enable 3DµF to understand that a new feature has been added, the user needs to "register" the new component into the code. This is done by modifying the constructor in the file: */src/app/featureSets/featureSet.js* as shown below.

```
...
import NewComponent from "../library/newComponent";

export default class FeatureSet {
    constructor(definitions, tools, render2D, render3D, setString) {
        ...
        this.__library = {
            ...
            "NewComponent": {"object": new NewComponent(), "key":"FLOW" },
            "NewComponent_control": {"object": new NewComponent(),
            "key":"CONTROL"}
        };
        ...
    }
```

6.3 Create UI Element

In order to add a new button to the 3DµF user interface, the user must include a new HTML button set into the UI. The following snippet should be added into */index.html*.

```
    <div id="button_drawer" class="mdl-layout__drawer">
        ...
        <nav class="mdl-navigation" id="<feature-??>">
            ...
            <div class="button_row">
                <a id="newComponent_button" class="mdl-button mdl-js-button
                mdl-js-ripple-effect mdl-button--raised feature-button">Mixer</a>
                <button id="newComponent_params_button" class="params-button
                mdl-button mdl-js-button mdl-button--icon">
                    <i class="material-icons">settings</i>
                </button>
```

```
            </div>
            ...
        </nav>
        ...
    </div>
```

6.4 Registering the Event Handlers

In order to register the event handlers for the button and to enable the activation of the corresponding placement tool many additions need to be made to the file */src/app/view/ui/-componentToolBar.js*.

6.4.1 Query Selectors

Create a reference field inside the *ComponentToolBar* object with the button ID used in the modified HTML (both tool and params).

```
export default class ComponentToolBar{
    constructor(viewmanagerdelegate){
        ...
        this.__newComponentButton =
        document.getElementById("newComponent_button");
        ...
        this.__newComponentParams =
        document.getElementById("newComponent_params_button");
        ...

        this.buttons = {
            ...
            "NewComponent" = this.__newComponentButton,
            ...
        }
    }

    ...
}
```

6.4.2 Event Handlers

Now set up the 'click' event handlers.

```
__setupEventHandlers() {
    this.__newComponentButton.onclick = function() {
        Registry.viewManager.activateTool("NewComponent");

        ref.setActiveButton("NewComponent");
        ref.__viewManagerDelegate.switchTo2D();
    };
}

__setupParamButtonEventHandlers() {
        this.__newComponentParams.onclick =
        ComponentToolBar.getParamsWindowCallbackFunction("NewComponent",
        "Basic");
}
```

6.4.3 Tool Association

Now that the event handlers are set up we need to link the event handler to the UX tool that performs the placement; this is done by modifying the */src/app/viewManager.js* where we instantiate the placement tool for each type of component that is selected. This can be of the type MultilayerPositionTool or ComponentPositionTool or CellPositionTool or a user-defined positioning tool.

```
setupTools() {
    ...
    this.tools["NewComponent"] = new MultilayerPositionTool();
    ...
}
```

7 Interchange Format

7.1 Overview

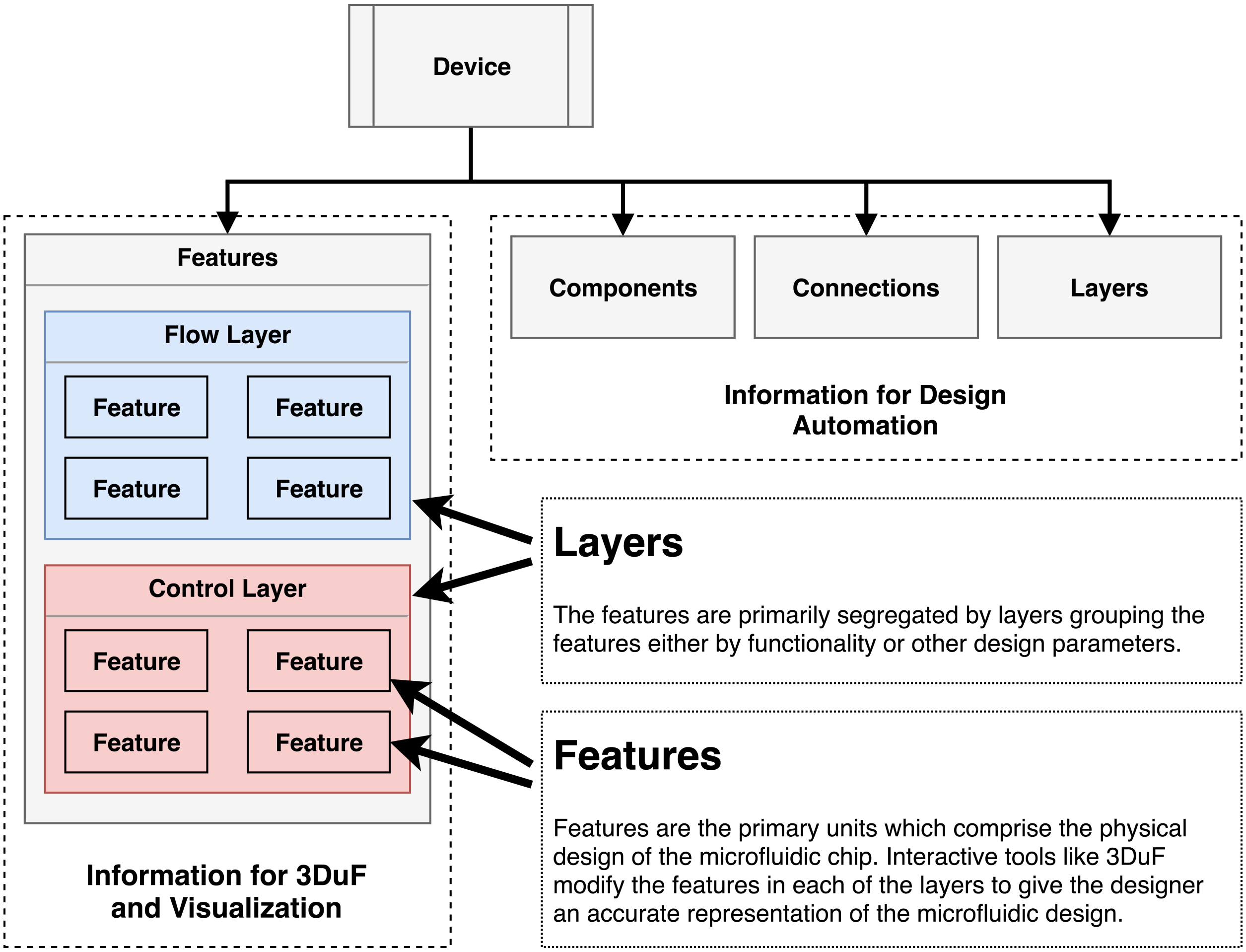

Figure 4: This figure is a representation of the overall schema used for describing the microfluidic devices.

One of the primary hurdles in technology transfer and research repeatability has been the standard for information exchange of microfluidics designs, especially in academic literature. Most works only include photographs of the microfluidic device with colored fluids and schematics have missing feature dimensions that are usually critical in order to replicate the design functionality. Even in cases where CAD designs are available, it takes manual effort to separate individual geometries and strokes that constitute a drawing from the CAD design, and the designs are not easily modifiable for reuse. As a part of this paper, we are also implementing a standard interchange format for displaying devices on visual editing tools. The interchange file is encoded in JSON[4] format, making the files generated by 3DμF both

[4]JSON (Javascript Object Notation) is a plain-text notation for describing data structures. It has parsing

human readable and accessible to any other CAD tool that intends to utilize the microfluidic design made using 3DμF.

The interchange format is specified in the JSON format and the major fields that constitute the design are the *Components*, *Connections*, *Layers* and *Features*. These can be further segregated by their intended function, i.e., for design automation or visualization. Figure 4 is a visualization of how all of the fields can be organized.

The *Features* field contains a set of *Layer* Collections. In turn, each *Layer* Collection contains a set of *Feature* objects. Each feature object contains the information necessary for manufacturing the geometries that need to be rendered on the artwork file. It is the artwork file (e.g., SVG) that would be used in the subsequent manufacturing process.

7.2 Feature Object

The feature object encapsulates the geometries that are manufactured. They contain vital information that is necessary for tools to generate artwork files for each of the layers that would be manufactured. Each object contains attributes that are necessary for describing the geometry and helping the rendering and artwork generation tool realize the design.

```
{
    "id": "unique-string",
    "type": "EDGE|XY|XYZ|Z",
    "macro": "draw-definition",
    "params": {
        "key1": "Value",
        "key2": "Value"
    }
}
```

Figure 5: This figure shows the minimal definition of the Feature object. Each of the attributes that are a part of this definition is vital to render the feature correctly.

The attributes shown in Figure 5 is the specified minimal definitions that are necessary for rendering the feature both in the artwork file and for visual tools such as 3DμF. The *id* attribute contains the unique identifier for each of the feature that can be used to identify individual features that need to be modified when automated design rule checks are performed. The *macro* is a string that tells the software tool how to draw the geometry from the *params* provided with the feature. Finally, the *type* attribute contains one of the following values: **EDGE, XY, XYZ, Z** (described in table 16). Each of the different values signifies how the tool would group the features to generate the artwork files.

libraries available in all major programming languages

8 Parametric Design Engineering

When designing analog integrated circuits, engineers first design the architecture of the circuit where they determine the different components and the connection topologies that perform the desired function. After completing the design of the architecture, they then adjust the electronic parameters of each of these components by adjusting their physical dimensions[5]. Since the functionality of a microfluidic component is dependent on its geometry, it is possible to parameterize the geometry of the microfluidic components. Automated design tools allow the engineer to tweak the functionality, the operation and the performance of the microfluidic design by varying each of the parameters. By composing the entire design with parametric components and connections, it is possible to design and test large complex design spaces by implementing the design of experiments methods used in [19].

Parameters capture the intent of the component designer, who considers how the variations of the parameters would continue to produce the intended effect. The capture of the 'intent' is fundamentally important when designing a standardized components library. The process of constructing device designs with parameters require the designer to incorporate individual parameters to each geometry that is drawn on the canvas. As the size of the design increases and the geometries are joined, all the parameters on individual geometries become interdependent. Typical designs require the designer to revisit how the parameters on the individual geometric feature are defined to ensure that the design does not become overconstrained. This also increases the burden on component designers to carefully pick the component parameters that would minimize the risk of over-definition. 3DμF eliminates the sources of most of these issues by atomically generating the individual component geometries based on the parameters used to define the component's functionality and dimensions.

8.1 Effort Metrics

In order to formally evaluate the effort of the engineer designing the microfluidic device, we introduce and utilize a quantitative measurement to quantify the effort involved with the design process called *Effort* (measured in *actions*). Since each microfluidic device design might vary vastly, we first break down the process of designing each type of component. The various steps involved with designing components can be assigned to two broad categories. Namely, *Design Effort* and *Parameterization Effort*. The total effort involved in creating each component is defined in Equation (1).

$$E_{Primitive} = E_{Design} + E_{Parameterization} \tag{1}$$

The *Effort* metrics are a heuristic metric that approximate the effort involved in designing a device. They are **not** an exact quantification of the design process. By tuning the values used in Table 5 and Table 4 one can increase the accuracy of the quantification. We selected

[5] The electronic properties of integrated circuit components are determined by their material properties which in turn depend on the dimensions of the features on the semiconductor substrate.

each of the costs for this study from user experiences drawing geometries with CAD tools and examining the complexity of the drawing routines in the 3DµF code. Readers are encouraged to utilize the spreadsheet provided in the SI and generate the *Effort* metrics with different cost values.

8.1.1 Design Effort

Design Effort - CAD Tools The Design Effort encapsulates all steps typically involved with generating designs. The steps and the associated variables used to quantify the effort can be seen in Table 2.

Step	Description	Design Effort Variable	Symbol
1.	Creating the base design[6]	Base Cost	C_{Base}
2.	Replicating the base design	Procedural Scaling Formula	$f_{Procedural}(X)$
3.	Scaling the design	Scale Factor	X

Table 2: Design Effort - CAD Tools

$$E_{Design} = C_{Base} \times f_{Procedural}(X) \tag{2}$$

Design Effort - 3DµF Since 3DµF is built with tools that automatically create the component geometry with a single action. Hence it can be seen that $E_{Design} = 1$.

8.1.2 Parameterization Effort

Parameterization Effort - CAD Tools The Parameterization Effort encapsulates all the steps typically involved with making the geometry parametric. The steps and the associated variables used to quantify the effort can be in Table 3.

Step	Description	Design Effort Variable	Symbol
–	Geometric Parameters Defining the Geometry	Number of Parameters	N_{Params}
1.	Identification of Orthogonal Parameters	Identification Cost	$C_{Identification}$
2.	Setting Geometry Constraints in Tool	Setting Constraint Cost	$C_{Constraint}$
3.	Setting Parameter Values	Set Value Cost	C_{Value}

Table 3: Parameterization Effort - CAD Tools

$$E_{Parameterization} = C_{Identification} + N_{Params}(C_{Constraint} + C_{Value}) \tag{3}$$

[6]This is usually equivalent to the number of vertices or the total number polygons in the geometry

Parameterization Effort - 3DμF Since 3DμF is built with tools that procedurally generate the component geometry, the geometries are inherently parameterized, and the user is only burdened with the task of setting the values as seen in Equation (4).

$$E_{Parameterization} = N_{Params} \times (C_{Identification}(Simple) + C_{Value}) \tag{4}$$

8.1.3 Constants and Assumptions

Base Drawing Cost (C_{Base} The *Base Drawing Cost* is the cost (# of *actions*) required to draw the basic geometry on the design canvas, this is especially useful when dealing with geometries that have repeating geometry, since we calculate the total cost by multiplying the C_{Base} by the scaling factor $f_{Procedural}$. The base costs used for the different components in this manuscript are given in Table 6. Since there might be variations in how a designer might construct the design, we took approximated the number of actions to be the number of vertices/base polygons the user would have to create and merge to create the geometry. **Note: This is an approximation.**

Value Setting Cost (C_{Value}) The *Value Setting Cost* is the cost (# of *actions*) involved in setting the correct numerical value of the parameter to the geometric constraint. The value chosen for C_{Value} in this manuscript is given in Table 4. The value 1 was chosen because it would take the user approximately 1 *action* to select and set the numerical value of the constraint.

Constraint Creation Cost ($C_{Constraint}$) The *Constraint Creation Cost* is the cost (# of *actions*) involved in setting the geometric constraint to a given set of vertices/lines/polygon in the drawing canvas. The value chosen for $C_{Constraint}$ in this manuscript is given in Table 4. The value 4 was chosen because it would take the user approximately 4 *actions* to set a geometric constraint, namely: Activate the tool, Identify and Select 2 vertices/edges, setting the initial value/parametric variable.

Cost	Symbol	Value
Value Setting Cost	C_{Value}	1
Constraint Creation Cost	$C_{Constraint}$	4

Table 4: Parameteric Design Costs

Identification Cost ($C_{Identification}$) The variable *Identification Cost* ($C_{Identification}$) which involves the designer to identify orthogonal parameters that do not get disturbed by design operations that are typically performed by the designer. Since the process of identification is subject to vary for each, conservative estimates were determined for components of different

complexity, and their respective classification in design and parametrization geometry is shown in Table 5 and Table 6 respectively.

Description of Complexity	Class	$C_{Identification}$
Predefined parameter	Simple	1
Geometries consisting of a single closed shape	Nominal	10
Geometries consisting of multiple closed shapes	Composite	20
Geometry that repeats geometries	Linear	20
Geometry that procedurally repeats geometries	Polynomial	40

Table 5: Identification Costs used for $C_{Identification}$

Component	$C_{Identification}$ Class	$f_{Procedural}$ Formula	Base Cost	N_{Params}
Mixer	20	n	6	4
Rotary Mixer	20	–	15	5
Gradient Generator	40	n^2	6	5
Valve	1	–	2	2
Valve3D	10	–	3	5
Transposer	20	–	25	5
Mux	40	$n^2/2$	8	7
Reaction Chamber	1	–	1	2
Diamond Chamber	10	–	6	3
Long Cell Trap	20	n	2	5
Square Cell Trap	10	–	6	3
Port	1	–	1	1
Channel	1	–	1	1
Tree	40	$n^2/2$	4	4
Droplet Generator	10	–	8	6

Table 6: Drawing, Parameterization Complexity and Base Drawing Costs

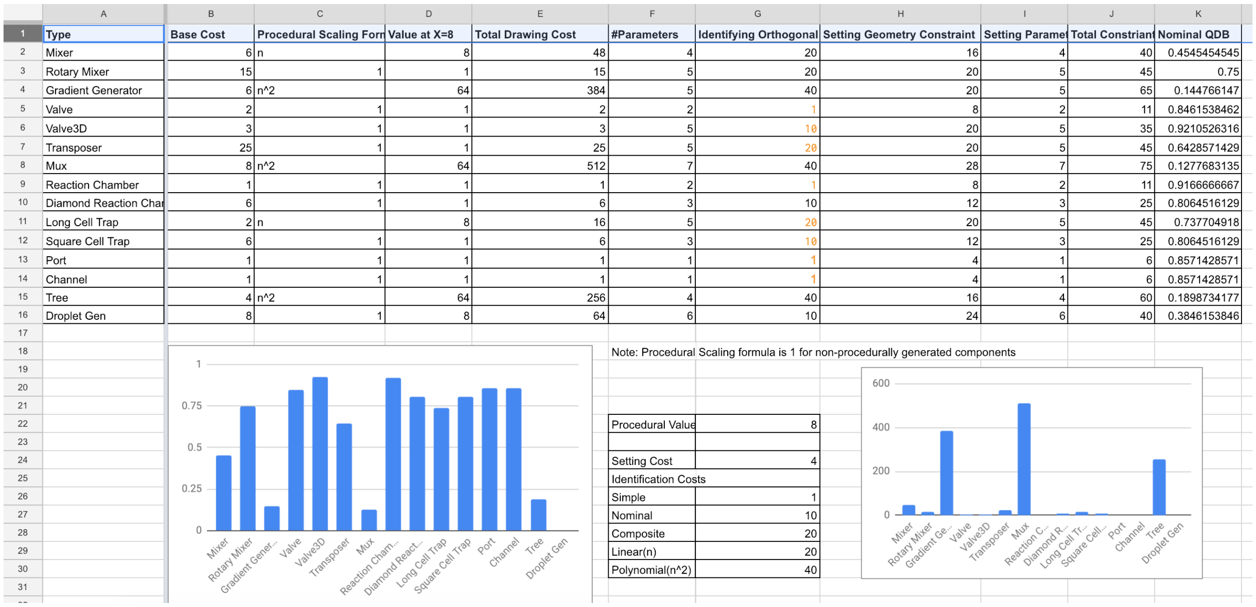

Type	Base Cost	Procedural Scaling Forn	Value at X=8	Total Drawing Cost	#Parameters	Identifying Orthogonal	Setting Geometry Constraint	Setting Paramet	Total Constriant	Nominal QDB
Mixer	6	n	8	48	4	20	16	4	40	0.4545454545
Rotary Mixer	15	1	1	15	5	20	20	5	45	0.75
Gradient Generator	6	n^2	64	384	5	40	20	5	65	0.144766147
Valve	2	1	1	2	2	1	8	2	11	0.8461538462
Valve3D	3	1	1	3	5	10	20	5	35	0.9210526316
Transposer	25	1	1	25	5	20	20	5	45	0.6428571429
Mux	8	n^2	64	512	7	40	28	7	75	0.1277683135
Reaction Chamber	1	1	1	1	2	1	8	2	11	0.9166666667
Diamond Reaction Chai	6	1	1	6	3	10	12	3	25	0.8064516129
Long Cell Trap	2	n	8	16	5	20	20	5	45	0.737704918
Square Cell Trap	6	1	1	6	3	10	12	3	25	0.8064516129
Port	1	1	1	1	1	1	4	1	6	0.8571428571
Channel	1	1	1	1	1	1	4	1	6	0.8571428571
Tree	4	n^2	64	256	4	40	16	4	60	0.1898734177
Droplet Gen	8	1	8	64	6	10	24	6	40	0.3846153846

Note: Procedural Scaling formula is 1 for non-procedurally generated components

Procedural Value	8
Setting Cost	4
Identification Costs	
Simple	1
Nominal	10
Composite	20
Linear(n)	20
Polynomial(n^2)	40

Figure 6: Screenshot of the Design Effort Excel Sheet (part of the supporting data)

8.2 Design Effort of a MIXER

In order to compute the component *Effort* required for drawing the mixer, we compute the E_{Design} defined by Equation (2) and the $E_{Parameterization}$ defined by Equation (4) separately and then compute the total *Effort* ($E_{Primitive}$) defined by Equation (1).

8.2.1 Design Effort (E_{Design})

The steps shown below follow the procedure followed in Table 2

Step 1. In order to compute the effort required to design the device, we first compute the effort required to create the base of the design which we need to replicate. This value is captured in the term C_{Base}. We can get the value $6\,actions$ for C_{Base} from Table 6.

Step 2. Now that have computed the C_{Base}, we now compute the scaling factor $f_{Procedural}$ for a MIXER of 8 bends. The term $f_{Procedural}$ captures how many times the base geometry is repeated and hence would have to be recreated by the user. Since different kinds of components have different levels of complexity in replication, $f_{Procedural}$ is defined a function $f(X)$ where $X = replicates$. Since the complexity of the MIXER is linear, we set $f(X) = X$ as given in Table 6. For the given value of $X = 8$, the scaling factor can be computed to be 8.

Subtotal: Hence, we can compute the E_{Design} defined by Equation (2) to be $48\,actions$.

8.2.2 Parameterization Effort ($E_{Parameterization}$)

Step 1. The first step in parameterization is the identification of the parameters and understanding which dimensions should be constrained. In order to capture the identification costs for a mixer we take the value given in Table 6 for the MIXER which is $20\,actions$.

Step 2. The second step in parameterization is the *Effort* required to add the geometric constraints in a CAD tool. We compute this by multiplying the number of parameters N_{Params} (Table 6) with the constraint cost $C_{Constraint}$ given in Table 4. This gives us the value of $16\,actions$.

Step 3. The third step in the parameterization is to set the actual value of the parameter, since the $C_{Value} = 1\,action$ (Table 4, the cost for this step would be $4\,actions$.

Subtotal: Hence the subtotal of $E_{Parameterization}$ is $40\,actions$

Total: Hence the subtotal of $E_{Primitive}$ is $88\,actions$ (defined by Equation (1)).

9 Case Study 1 - Devices from Literature

In order to show the capability of 3DµF , nine designs from the literature were chosen to be replicated and fabricated using 3DµF. This section contains a more detailed look at the designs that were fabricated and the associated analysis that was done using ImageJ to demonstrate the veracity of the designs specified in 3DµF. All of the design files are available at https://cidarlab.github.io/3DuF-Paper-Designs/.

Comprehensive Component Library Most system designers typically use a standard set of components when designing devices because it allows them to leverage their experience with specific geometries when dealing with unexpected behaviors. As can be seen in Figure 7, devices used for vastly different LoC applications along with their control infrastructure could be recreated using the standard set of components in 3DµF.

Custom Component Features In scenarios where specific custom geometries were required (single cell trap in Figure 7 F), the custom geometries were imported as custom components via the DXF import feature present in 3DµF. In order to address the issue where some of the designs are usually composed of just channels (Figure 7 D), 3DµF was modified to be able to create ad-hoc custom components out of a selection of FEATUREs, CONNECTIONs, and COMPONENTs. However, the most significant benefit of using the component library was the availability of components whose geometries are procedurally generated like those in *MIXER* (Figure 7 B, C) and *TREE* (Figure 7 I).

9.1 Device Descriptions

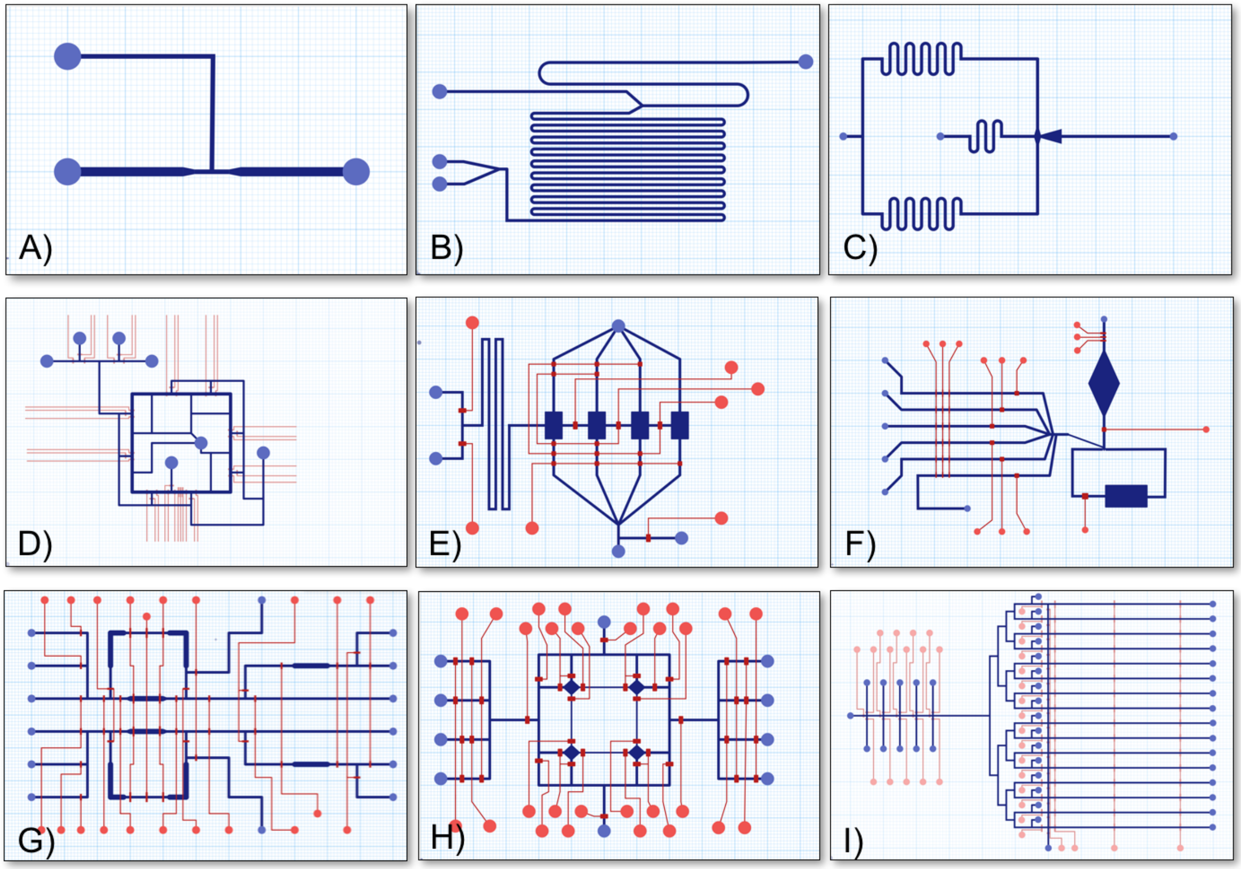

Figure 7: This figure contains recreations of the microfluidic chips from literature. They are : A) Quake Droplet Generator [25] B) Kobayashi Hydrogenation Chip [10] C) Nozzle Droplet Generator Chip [11] D) A single microchemostat unit [1] E) Dynamic Signaling Chip [27] F) Daridon Single Cell Trap [28] G) Kinase Radioassay Chip[5] H) Cell-Microenvironment Chip [15] I) Oligonucleotide Synthesis Chip[13]

Device 1.1 - Quake Droplet Generator This design as seen in Figure 7 (A) was referenced from the paper by Thorsen et. al. [25]. The design consists of a single droplet generator that was constructed by intersecting two perpendicular channels, in practice, many different droplet generators can be arrayed in a line to generate samples from multiple reagents. Engineers can modify the rate of droplet generation and the droplet size by varying the widths and heights of both the channels that constitute the droplet generator.

Device 1.2 - Kobayashi Hydrogenation Chip The Kobayashi Hydrogenation chip as seen in Figure 7 (B) is an example of a device that from literature [10] that is used for organic compound synthesis and shows the potential of 3DμF to create the devices using a set of the curved mixers. The user can modify the reaction area and volumes for specific reactions by

just modifying the parameters of the component. However one of the drawbacks of design is that the specialized surface used for the reaction cannot be specified in the tool.

Device 1.3 - Nozzle Droplet Generator Chip The Nozzle Droplet Generator [11] as seen in Figure 7 (C) is an example of a device that generates droplet at different sizes and frequency based on the geometry of the nozzle that initiates the droplet generation process. Users can easily vary the frequency and rates of droplet generation by changing geometric parameters.

Device 1.4 - Microchemostat Unit This device, as seen in Figure 7 (D) is a reproduction of a single functional unit as seen in the paper by Balagadde et al. [1]. This design is also a good example that shows the difficulty in designing devices that require replicated subunits that need to be automatically parameterized when tuning the performance of the device. Currently, 3DµF does not support the automated parameterization of these subunits, future versions of 3DµF would introduce algorithms that will compute parameters for both imported geometries and selected microfluidic subunits.

Device 1.5 - Dynamic Signaling Chip The Dynamic Signaling chip as seen in Figure 7 (E) is a reproduction of the device published by Wang et al. [27]. Since 3DµF stores, the internal connections and the valving between reaction chambers, future control automation algorithms can leverage the availability of this information along with the detailed geometric parameters that are used to generate valve control and timing sequences that can be used in executing complex protocols.

Device 1.6 - Daridon Single Cell Trap This device as seen in Figure 7 (F) is a reproduction of the device published in the paper by Wheeler et al. [28]. Since the single cell trap utilized by this device is unique the functioning of the corresponding assay, the single cell trap is implemented by importing the DXF design of the trapping chamber. Many of the devices in the literature that works with cells have unique geometries and designs that take advantage of both fluid mechanics and particle motion when subjected to unique operating conditions; hence the user can take advantage of the DXF import functionality for unique components that are impractical to implement into 3DµF.

Device 1.7 - Kinase Radioassay Chip The device seen in Figure 7 (G) was designed for performing the Kinase Radioassay [5] the device was one of the harder devices to reproduce since it uses channels of different widths throughout rather than take advantage of reaction chamber components, since some of the valves as sieve and pump valves which are currently not a part of the standard component library. This design is but one of the examples that is representative of the lack of standardization of the parts, formalization of the components and their functionalities. By standardizing these components, the debugging and integration

challenges associated with incorporating these functions into a device can be mitigated systematically.

Device 1.8 - Cell-Microenvironment Chip The Cell-Microenvironment chip as seen in Figure 7 (H) was easily reproduced using 3DµF since the design only consists of Ports, Channels, Valves, and reaction chambers. While the network was tedious to build using 3DµF, future algorithmic extensions would help engineers to scale the complexity of device with minimal effort quickly.

Device 1.9 - Oligonucleotide Synthesis Chip The device represented in Figure 7 (I), though it seems complicated is easy to reproduce using Trees. The entire design can be scaled to accommodate a more significant number of synthesis chambers by just changing a single parameter on the Tree component.

9.2 Milled Devices

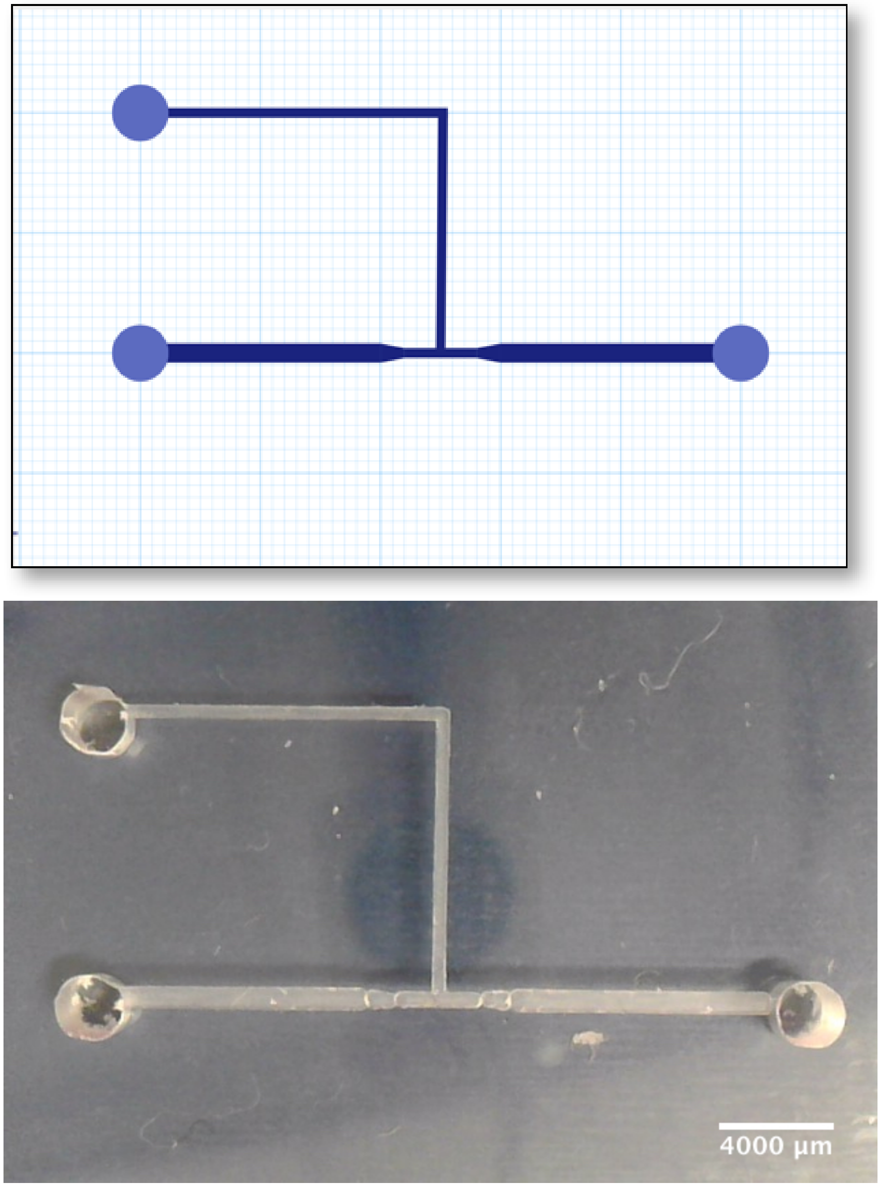

Figure 8: Quake Droplet Generator - Top: 3DµF Design Bottom: Milled Device

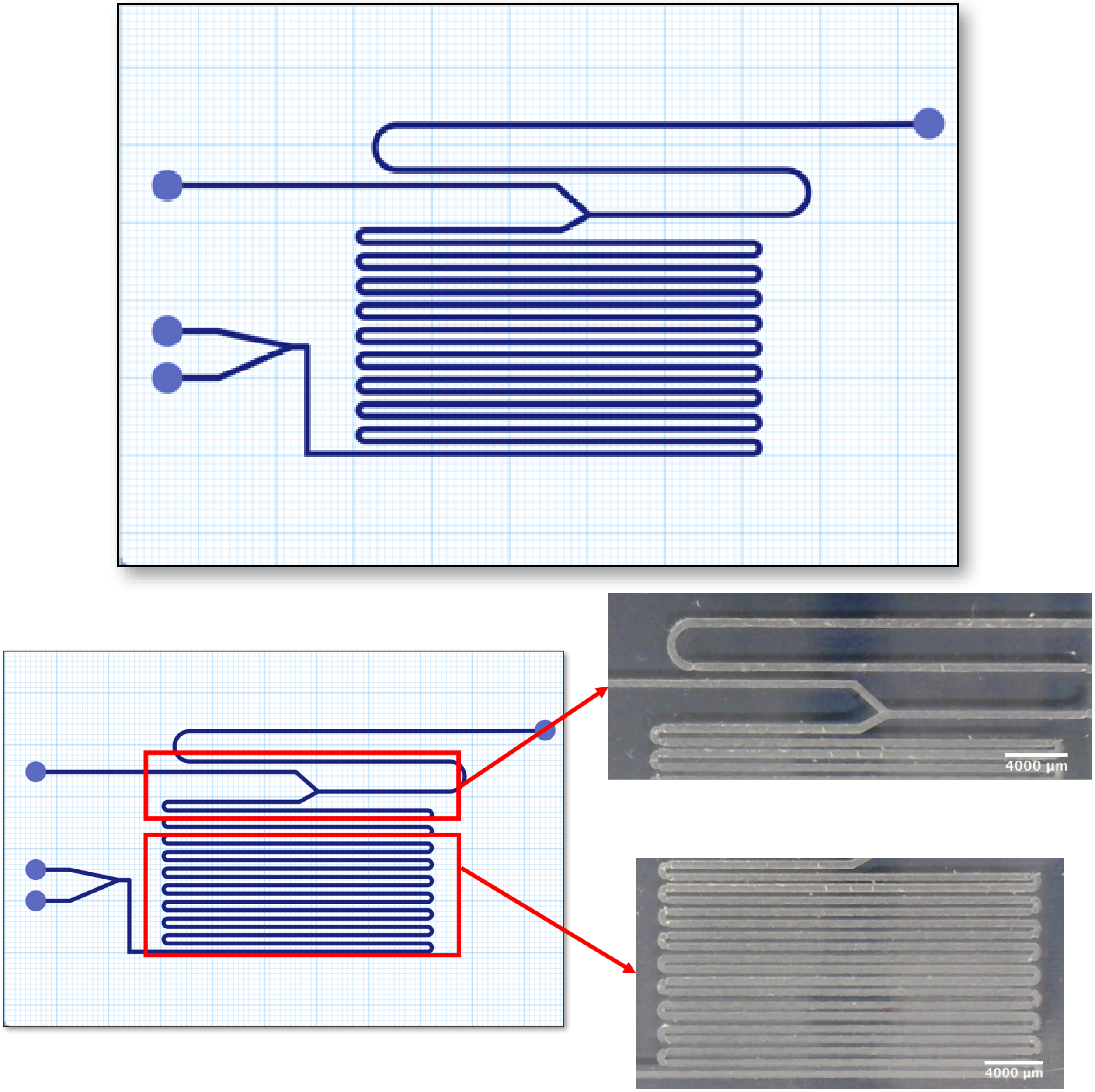

Figure 9: Kobayashi Hydrogenation Chip - Top: 3DµF Design Bottom: Milled Device

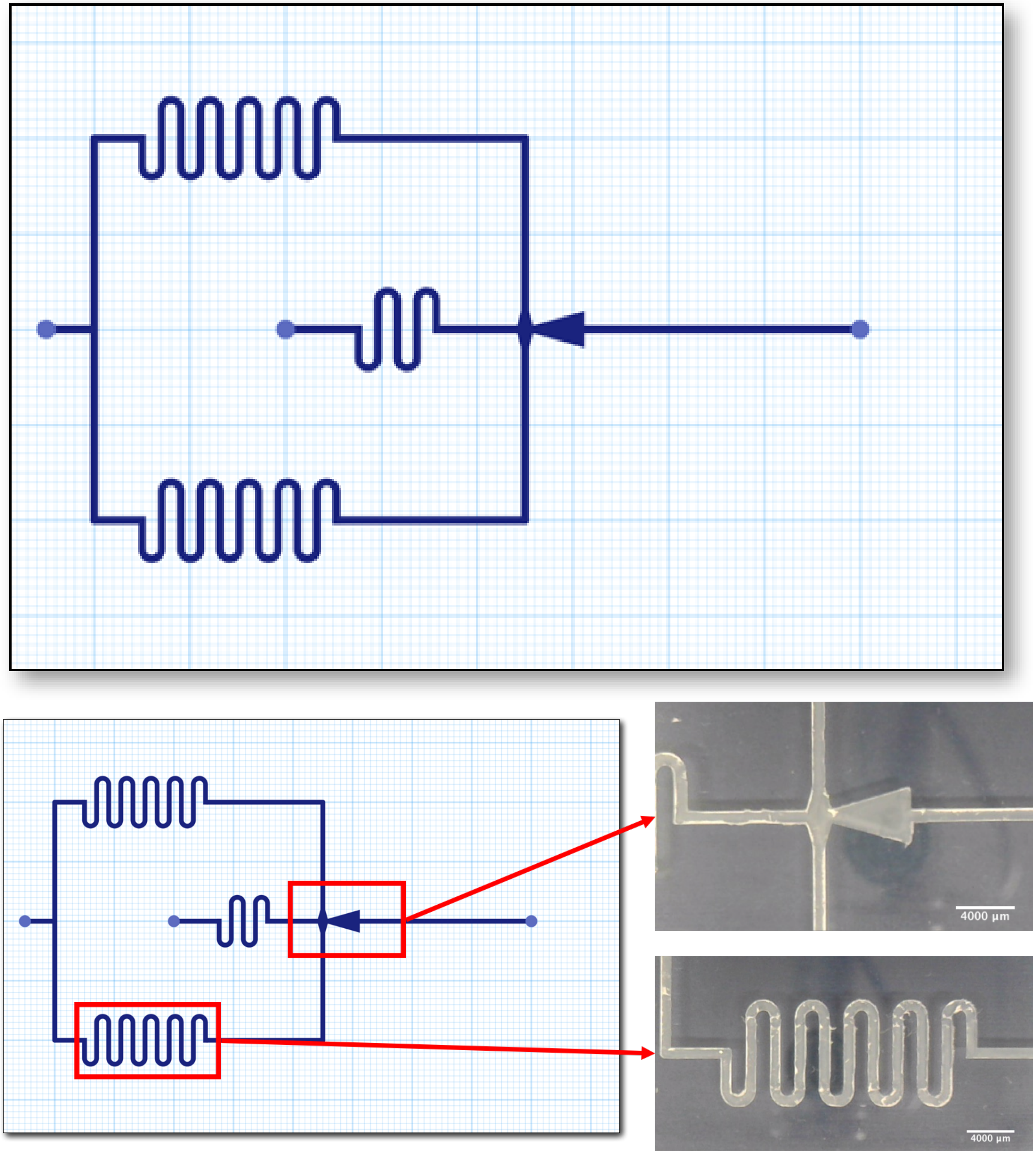

Figure 10: Nozzle Droplet Generator - Top: 3DμF Design Bottom: Milled Device

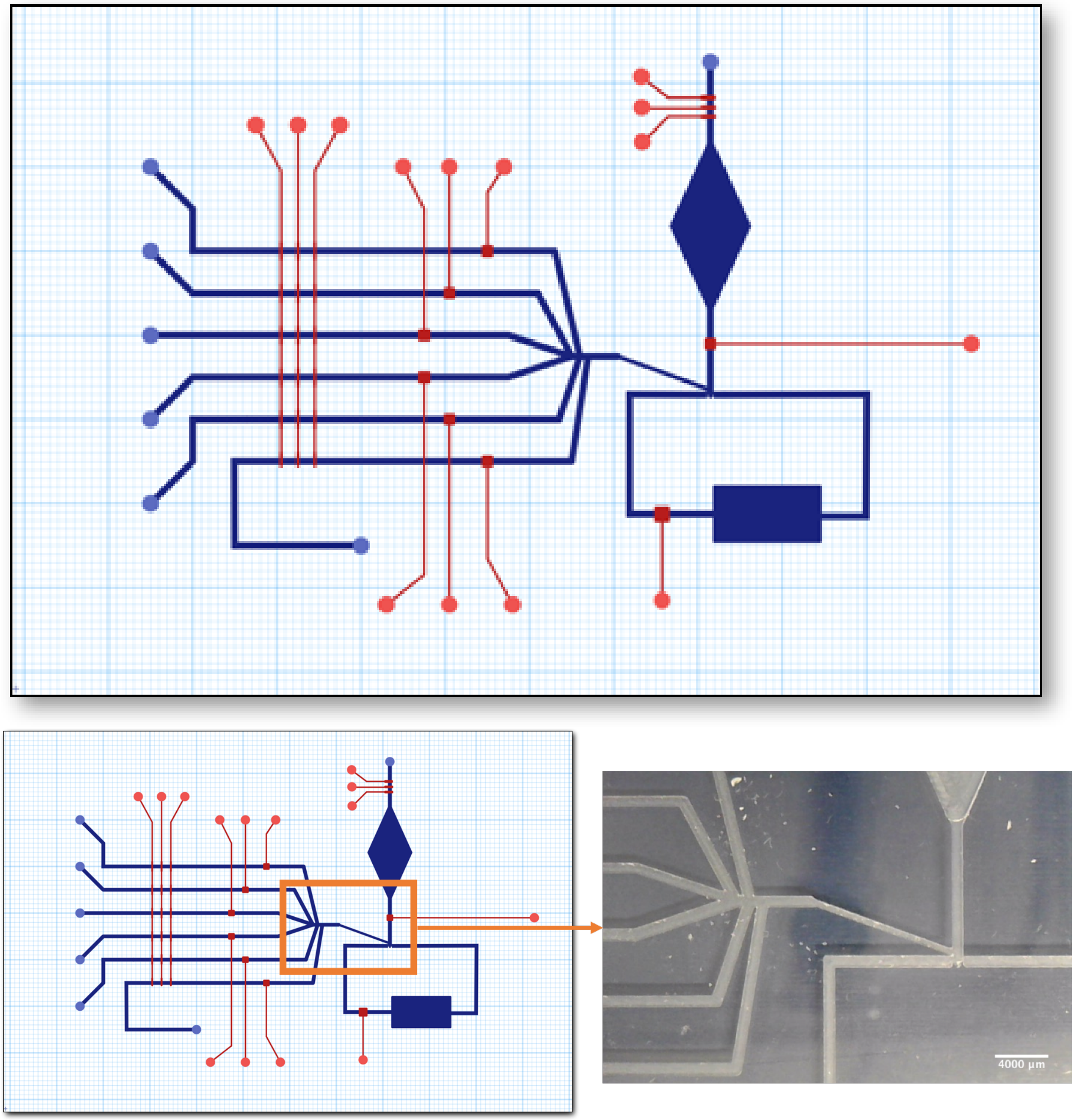

Figure 11: Daridon Single Cell Chip - Top: 3DµF Design Bottom: Milled Device

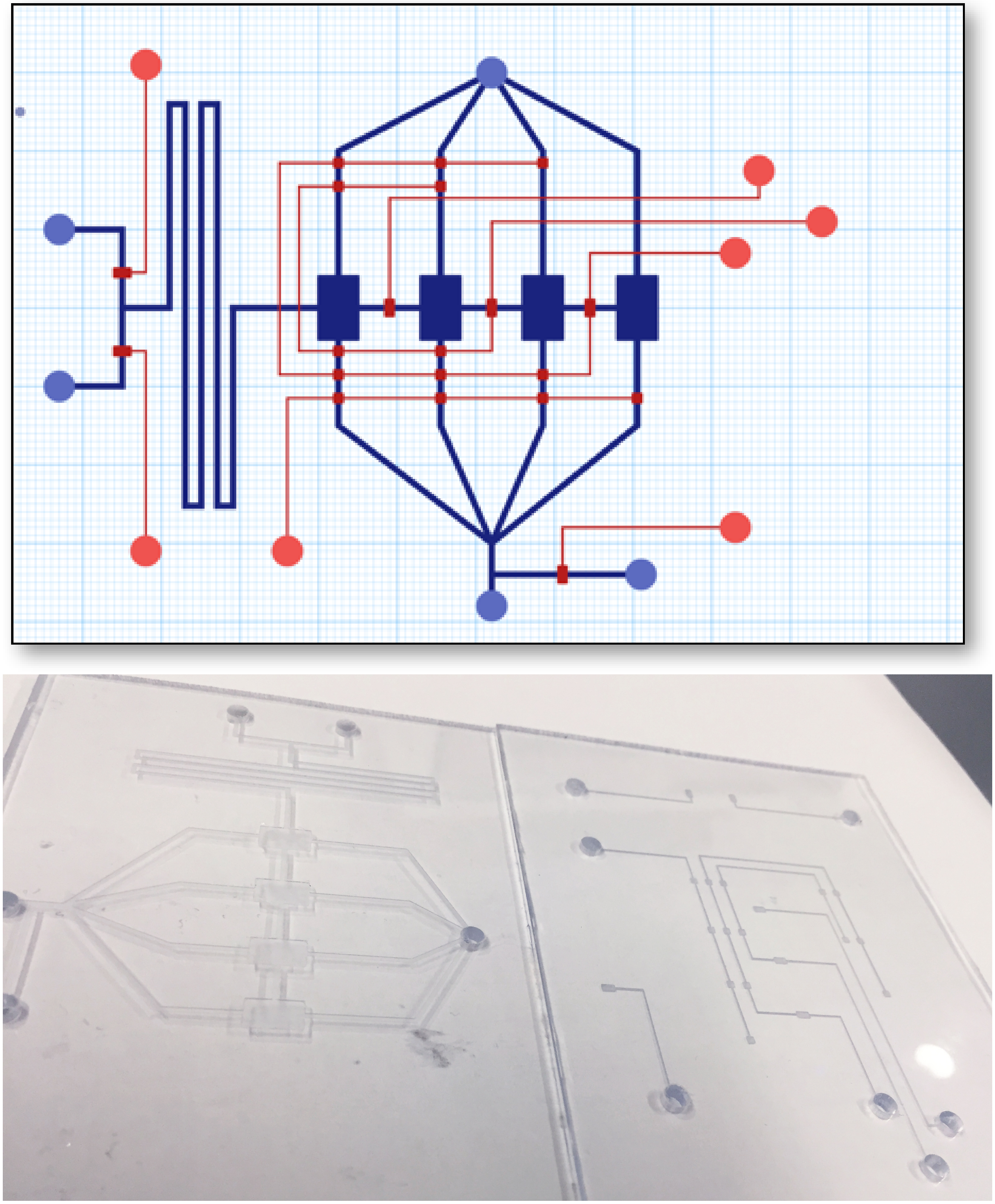

Figure 12: Dynamic Signaling Chip - Top: 3DµF Design Bottom: Milled Designs

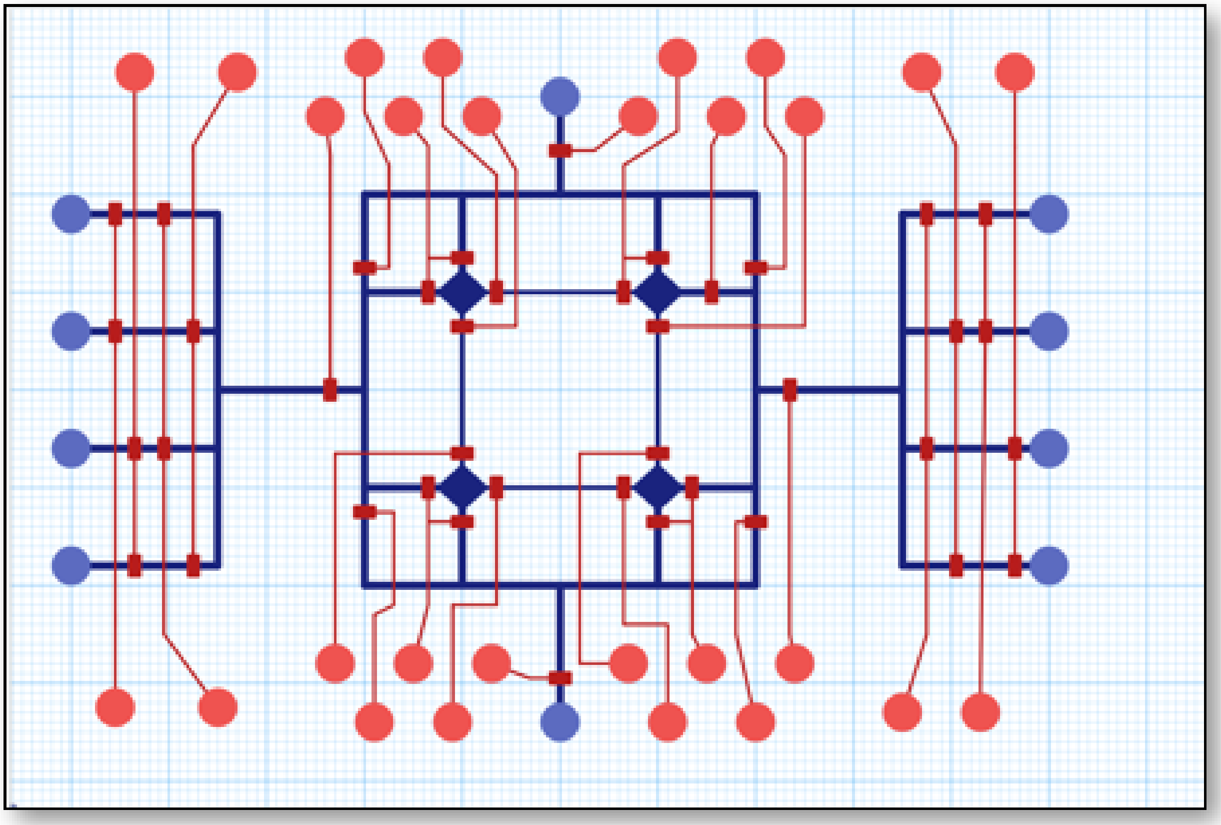

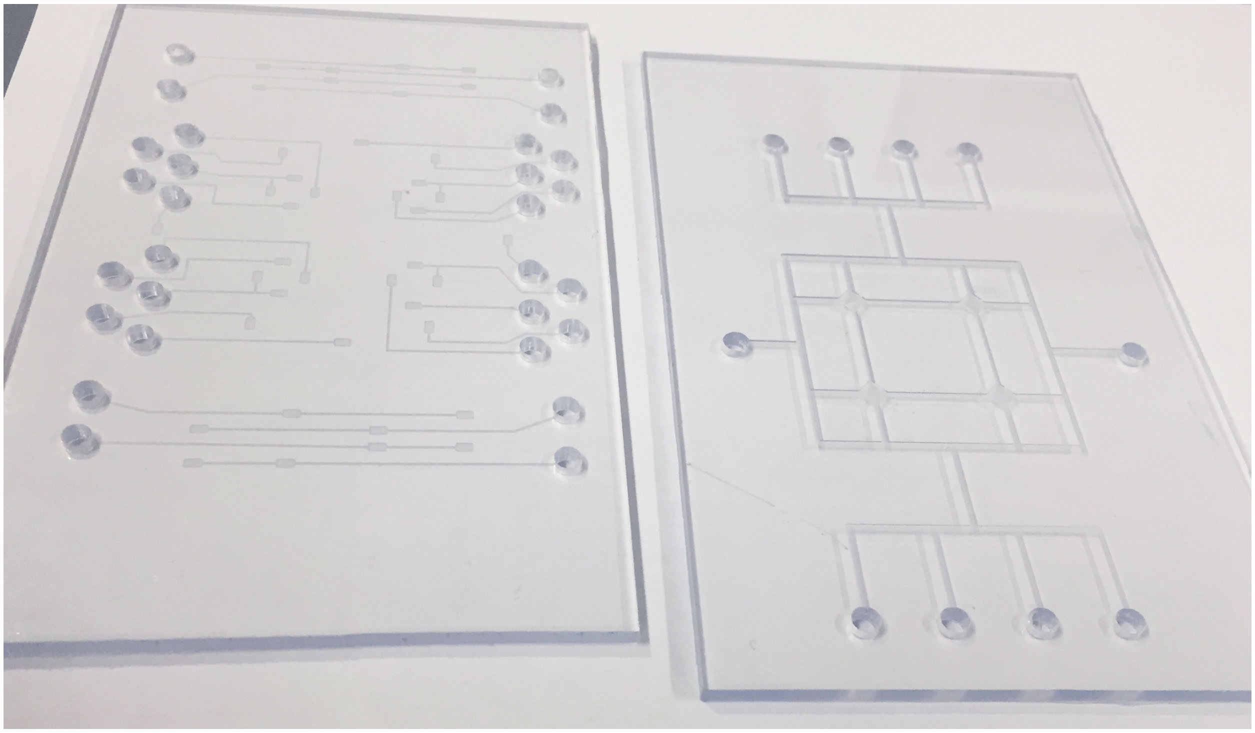

Figure 13: Cell Microenvironment Chip - Top: 3DμF Design Bottom: Milled Device

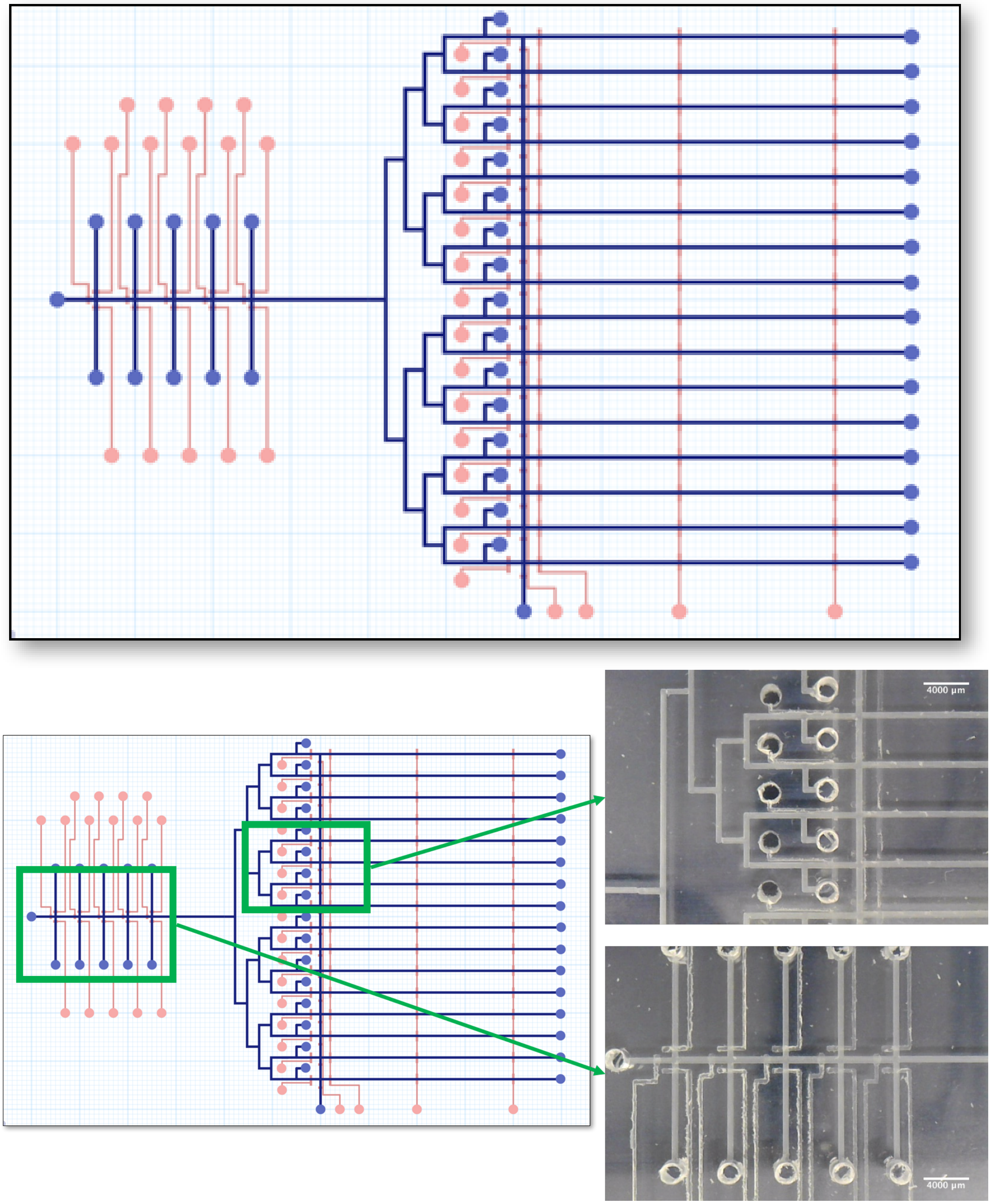

Figure 14: Oligonucliotide Synthesis Chip - Top: 3DμF Design Bottom: Milled Device

9.3 Designs Website

Each of the design presented in the paper is available for editing via https://cidarlab.github.io/3DuF-Paper-Designs/

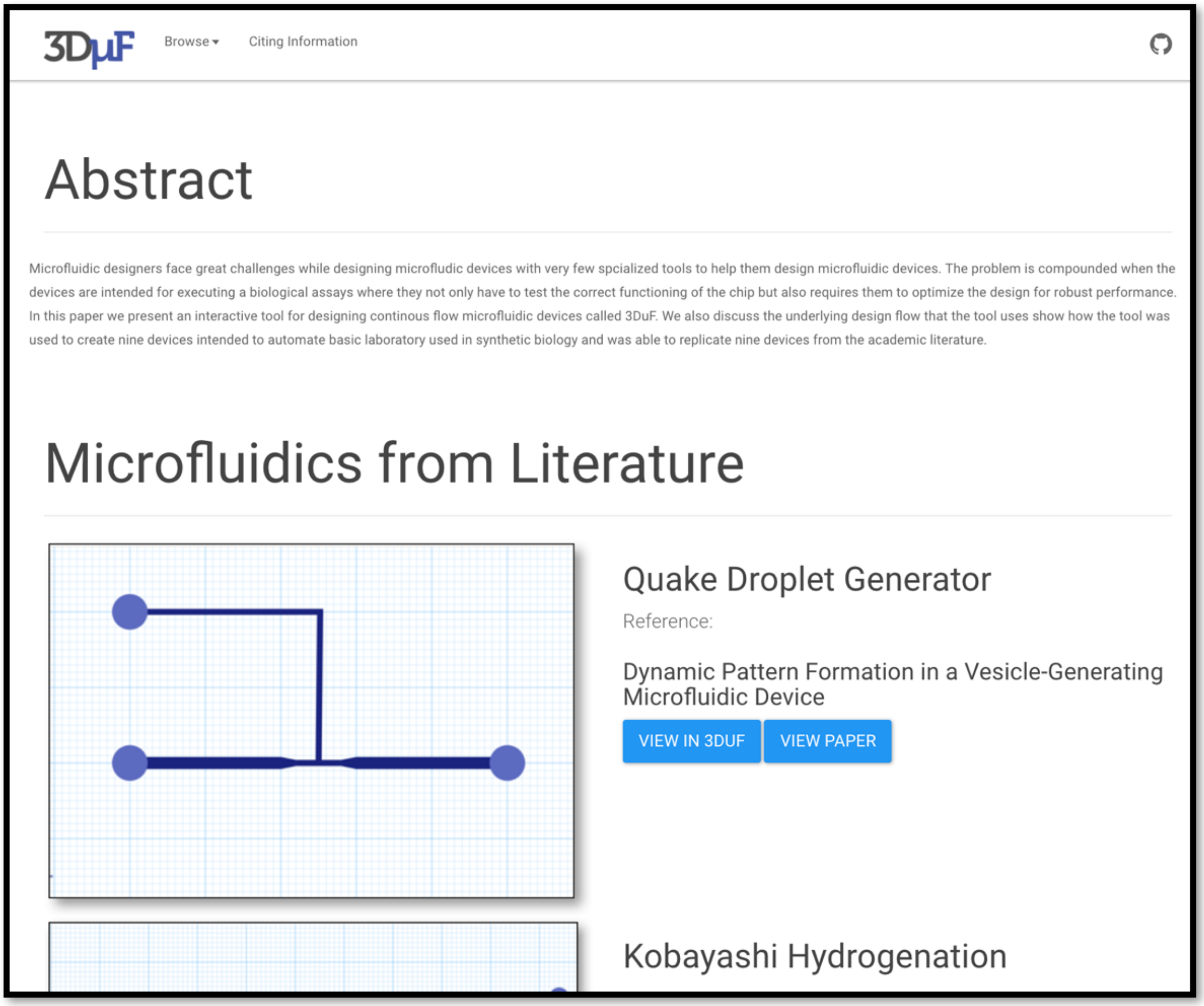

Figure 15: Preview of the website

9.4 Effort Calculation Script

The script used for calculating the efforts for the benchmark is available at https://github.com/CIDARLAB/3DuF-Paper-Designs/blob/master/characterize/benchmarkefforts.py

10 Case Study 2 - Modular System Design

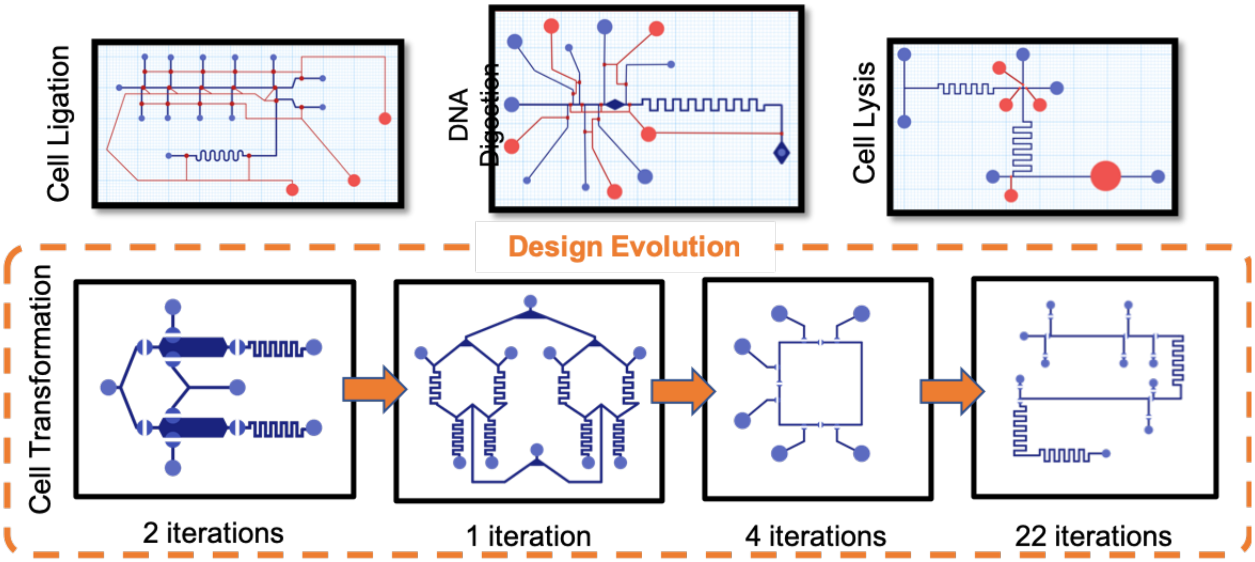

Figure 16: Overview of the different devices created using 3DμF for the MARS project.

Each of the devices shown in the paper can be found online on the paper's associated website, https://cidarlab.github.io/3DuF-Paper-Designs/. Additional information along with the artwork files, 3DμF designs, and the demonstration videos can be seen on the 2017 Boston University iGEM Hardware team `http://2017.igem.org/Team:BostonU_HW/Demonstrate`. The links to the specific pages for each of the devices is given in Table 7.

#	Name	Info	Video
1	Cell Sorting	http://2017.igem.org/Team:BostonU_HW/Isolation	Yes
2	Antibiotic Resistance	http://2017.igem.org/Team:BostonU_HW/Antibiotic	No
3	Cell Ligation	http://2017.igem.org/Team:BostonU_HW/Ligation	No
4	DNA Digestion	http://2017.igem.org/Team:BostonU_HW/Digestion	No
5	Cell Lysis	http://2017.igem.org/Team:BostonU_HW/Lysis	Yes
6	Fluorescence	http://2017.igem.org/Team:BostonU_HW/Fluorescence	No
7	Cell Transformation	http://2017.igem.org/Team:BostonU_HW/Transformation	Yes

Table 7: MARS Devices

10.1 Discussion

All 9 MARS modular microfluidic chips were fabricated using the *Makerfluidics*[20] protocol. In brief, SVG design files are generated using 3DμF. Using the CNC mill and its accompanying software, the designs are etched onto polycarbonate pieces, creating the flow and control layers. A thin piece of PDMS (Polydimethylsiloxane) is inserted between the

properly-aligned layers. The edges of the device are clamped together using binder clips, and the assembled chip is then desiccated. Once desiccated, the chip can be tested.

While useful for rapid and inexpensive iteration, *Makerfluidics*[20] has design and manufacturing limitations. Chip feature sizes and depths are limited by available end mills. The lack of permanent bonding of PDMS and polycarbonate means that chips have a vulnerable seal that can be prone to leakage. This is most often seen in chips containing valves, as PDMS is being stretched, leaving small areas for the liquid to penetrate during valve manipulation. Overcoming and adapting to these limitations is integral to the microfluidic design process. Furthermore, in practice, the manufacturing protocols vary between manufacturers and research labs. Hence, adapting microfluidic concepts to new manufacturing protocols is part of the standard design process.

The MARS Transformation Chip, as seen in Figure 16 went through 2 significant conceptual design changes. The initial design was a modified reproduction of the device published by Hui et al. [16] which utilizes metering and a peristaltic pump to measure and mix exact proportions of liquids. The metering functionality was isolated and replicated, as transformation requires accurate proportions of plasmid to cells. However, there were difficulties in replicating the original design. Since 3DμF was not capable of replicating the original devices circular design, a modified square design was used instead. The distance between valves created air gaps in the liquid, decreasing the accuracy of the measurements. Sealing limitations meant that the devices clustered valves, integral to the metering functionality, were also accompanied by leakage issues.

The second major design attempted to improve and build upon the metering functionality and to overcome manufacturing limitations. Instead of a circular/square shape, the metering section was redesigned to be linear. Valves were relocated to reduce air gaps. Smaller ports were used to reduce leaking. A time-dependent-mixer was added to perform heat-shock transformation for precisely 30 seconds. Pressure drops caused by square mixers required them to be replaced by curved mixers. In total, the device would measure the correct volumes of plasmid and cells, mix them, then perform heat-shock based transformation. However, the manufacturing process created the additional design and testing limitations. Feature size limitations meant that valves could not be as small as desired. Sealing limitations meant that the clustered valves continued to experience significant leakage. The design underwent continuous iteration regarding valve shape, size, depth, location, and distance. Over 20 iterations were dedicated to modifying valves. While leakage persisted through all iterations, the severity was able to be decreased. Leakage associated with valves also rendered time-dependent-mixers unreliable.

The significant design challenge in this device was adapting an established microfluidic concept to a new manufacturing protocol with different limitations. The final device is tentatively functional. It can, in theory, perform metering and time-dependent-mixing, but unreliable. Leakage is the primary issue still encountered limiting its functionality. Because of this persistent issue, heating elements have also not been tested in this device.

10.2 Transformation

Bacterial transformation is a commonly used protocol in synthetic biology. It can be used for a variety of functions, such as testing whether or not a genetic circuit is functional. Transformation allows bacterial cells, such as Escherichia coli, to take in and express external DNA fragments. Transformation consists of heat shock to damage cells and promote the taking up of external plasmids, recovery to prevent cells from dying, and a final culturing. From there, cells are analyzed.

This device underwent roughly four significant conceptual design changes, each with multiple design iterations and varying component parameters. Itemize evolved throughout four months. At each iteration, the designs were manufactured and tested for their functional correctness. The first design was dropped when the team realized that the shared inputs in the design allowed for contamination via diffusion and that the large chambers in the design often trapped air bubbles. The second iteration tried to implement a continuous flow system that addressed the issue of air bubbles.

Further testing showed that a significant impediment to the design was that it was difficult to dispense the correct reagent quantities necessary for the assay. In order to address the issue of metering the right amounts of fluids, we decided to adopt the metering system used in [16]. The rectangular design was discarded after four design iterations and adapted into a staged design to accommodate syringe pumps instead of the peristaltic pump design utilized in the original paper.

10.2.1 Chip Design

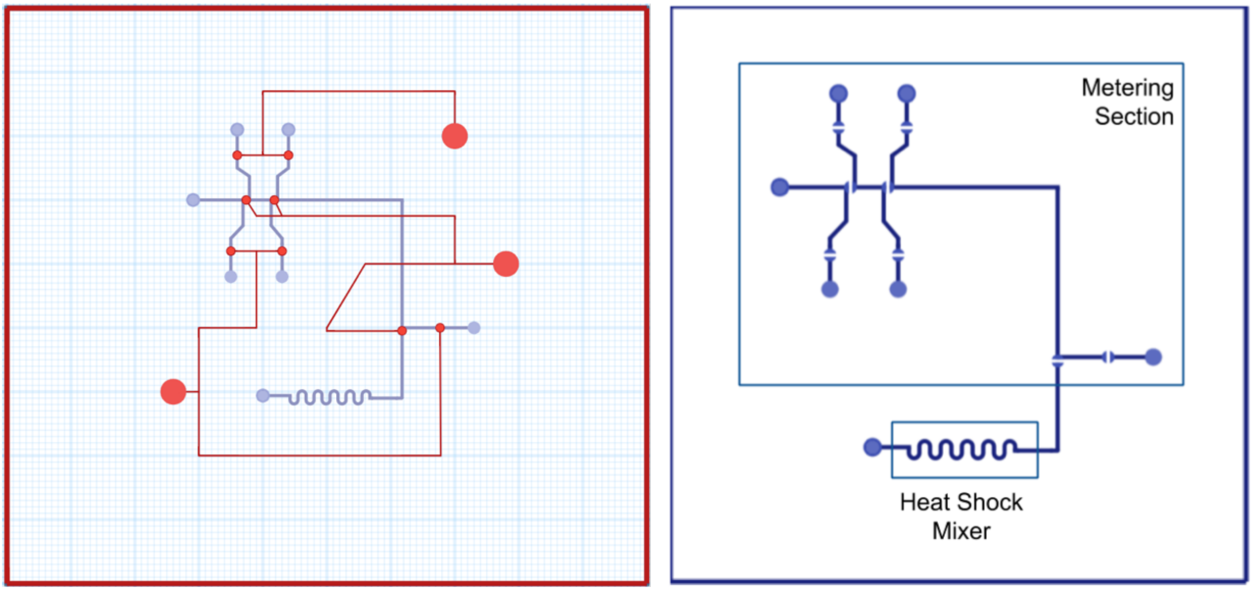

This microfluidic chip is designed to perform transformation. Suspended cells and plasmid are metered on the chip and are then mixed together. The solution then undergoes heat

shock in a time-dependent mixing element for exactly 30 seconds. The solution can then be pipetted out from the chip into a recovery tube on ice.

10.2.2 Design Layers

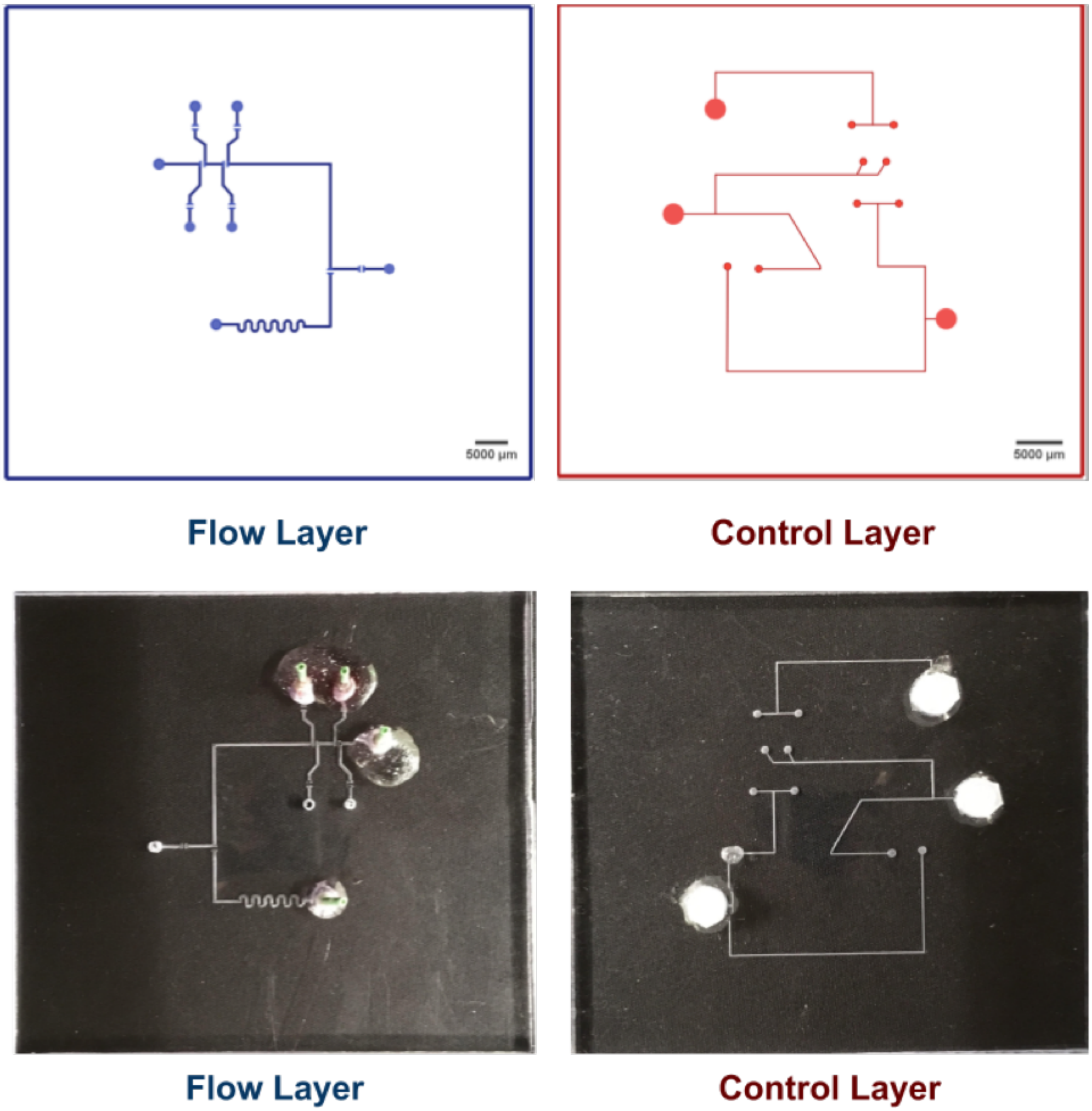

10.2.3 Inputs and Outputs

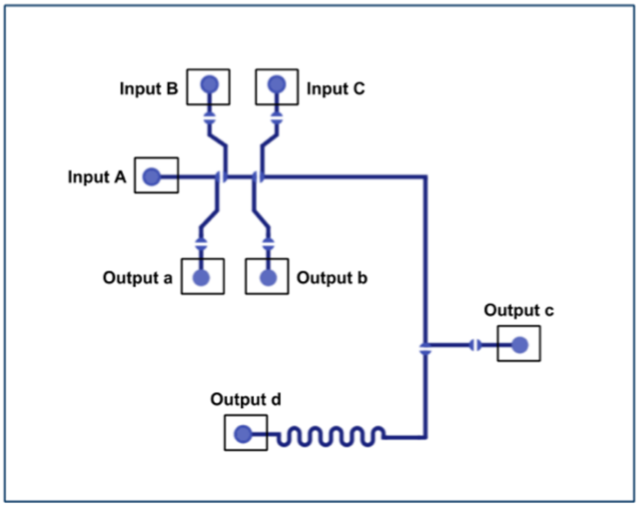

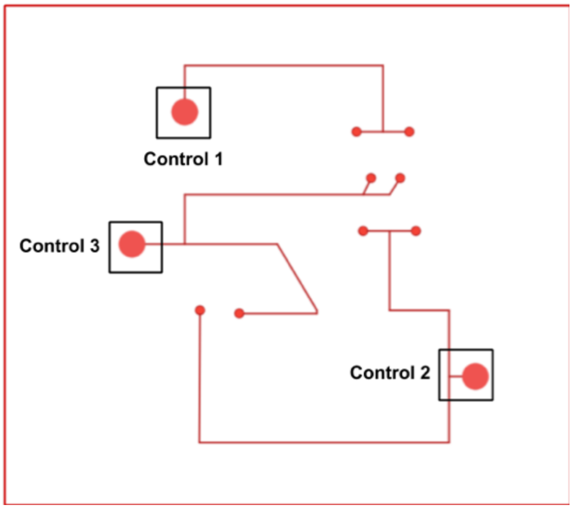

Name	Liquid	Flow Rate
A	Mineral Oil	0.5 mL/hour (metering)<br>0.835 mL/hour (pushing)
B	Plasmid<br>Represented by black colored water	0.5 mL/hour
C	Cell Suspension<br>Represented by red colored water	0.5 mL/hour

Table 8: Inputs

Name	Liquid
A	Excess Mineral Oil
B	Excess Plasmid
C	Excess Cell Suspension
D	Final Output

Table 9: Outputs

10.2.4 Protocol

Setup

1. Prepare 7 syringes
   (a) 1 filled with black colored water
   (b) 1 filled with red colored water
   (c) 1 filled with mineral oil
   (d) 3 empty 3 mL control syringes
2. Attach your syringe containing mineral oil to Input A
3. Attach your syringe containing black colored water to Input B
4. Attach your syringe containing red colored water to Input C
5. Attach your waste output tubing to Outputs a, b, and c; this liquid will be excess fluid
6. Attach your output tubing to Output d; this tube should connect to an eppendorf or other small collection receptacle located in a cold water bath
7. Attach three separate control syringes to Control 1, Control 2, and Control 3
8. If a heating element is being utilized, ensure this element is turned on and at the correct temperature

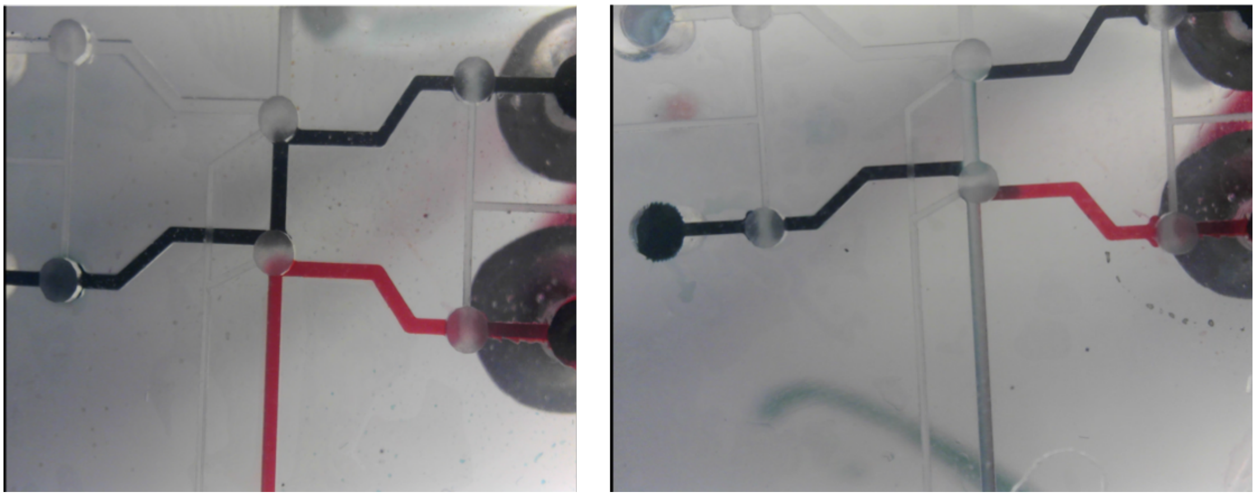

Figure 17: Left - Liquids filling up the metering section Right; Right - Pushing & Mixing of metered liquids

Execution

1. Open Control 1, then Control 2; you should feel significant resistance while you open these control valves

2. Begin flowing your mineral oil, black colored water, and red colored water at flow rates of 0.5 mL/hour each

3. Once the mineral oil, red colored water, and blue colored water have filled their metering sections and have begun filling the output port, ensure all of their syringe pumps have been turned off

4. Close Control 1, pause, then close Control 2

5. Open Control 3

6. Flow the mineral oil again at 0.835 mL/hour

7. The oil will push and mix the two colored waters

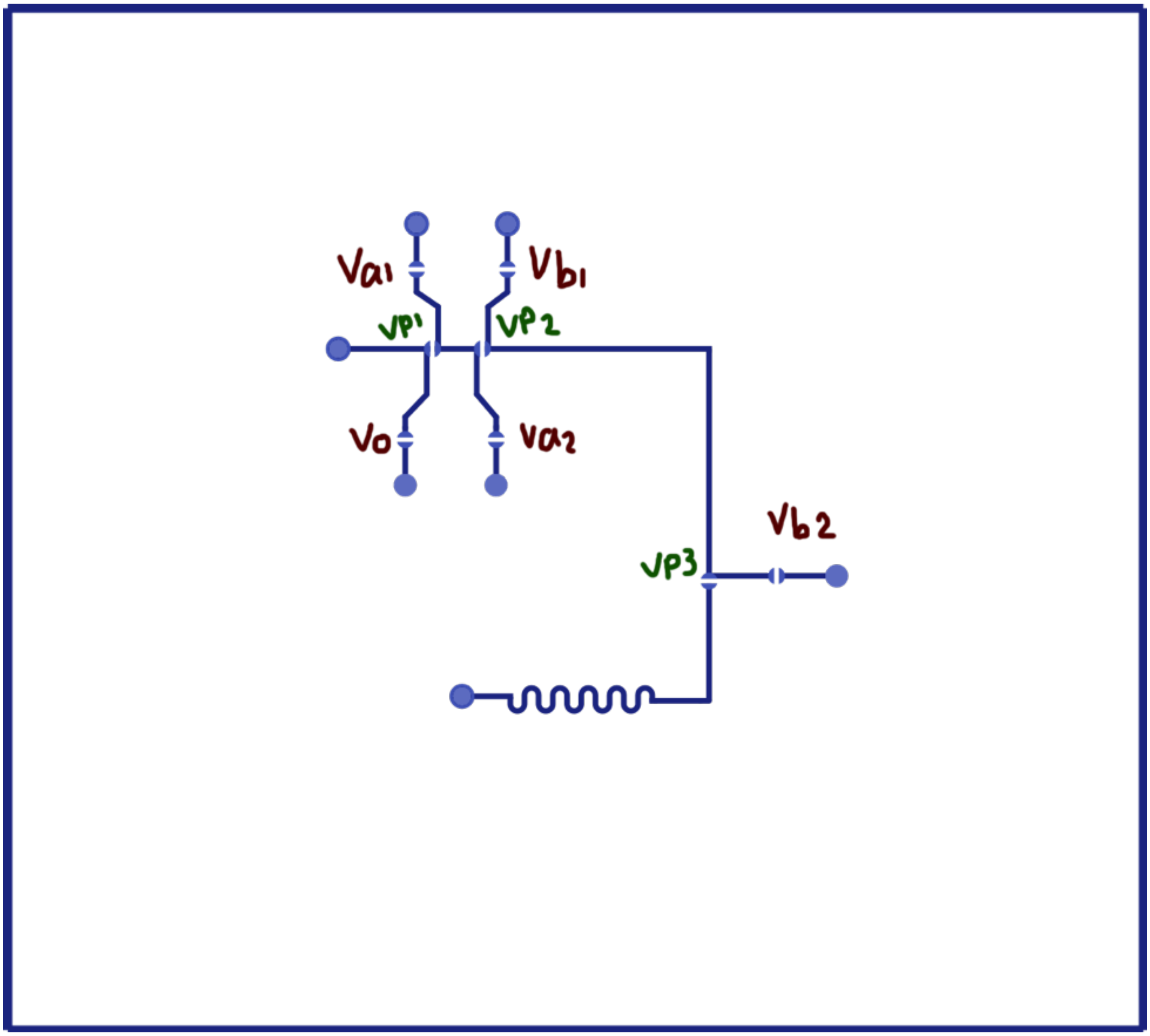

Figure 18: Sketch annotating the component names used for the device

Sequential Instructions All time units are in s.

```
SET va1 OPEN
SET vb1 OPEN
SET vb2 OPEN
SET va2 OPEN
SET vc OPEN
WAIT 150
SET va1 CLOSE
SET vb1 CLOSE
WAIT 2
SET va2 CLOSE
SET vb2 CLOSE
SET vc CLOSE
WAIT 2
```

```
SET vp1 OPEN
SET vp2 OPEN
SET vp3 OPEN
WAIT 200
SET vp1 CLOSE
WAIT 2
SET vp2 CLOSE
WAIT 2
SET vp3 CLOSE
```

10.3 Ligation

Ligation is a commonly used protocol in synthetic biology. In molecular cloning ligation is the process by which external DNA is inserted into a vector DNA, often a plasmid, using the enzyme DNA ligase. The newly formed DNA, or recombinant DNA, can then be analyzed or transformed.

Molecular cloning ligation is the process by which external DNA is inserted into a vector DNA, often a plasmid, using the enzyme DNA ligase. The newly formed DNA, or recombinant DNA, can then be analyzed or transformed. In this device, the T4 DNA Ligase Buffer, vector DNA, insert DNA, water, and T4 DNA Ligase are metered on the chip and are then mixed. The solution then undergoes incubation, after which the recombinant DNA solution can be pipetted out from the chip and used in further molecular cloning procedures

10.3.1 Chip Design

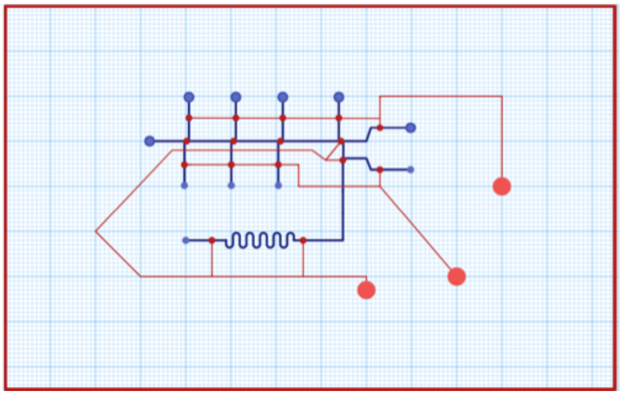

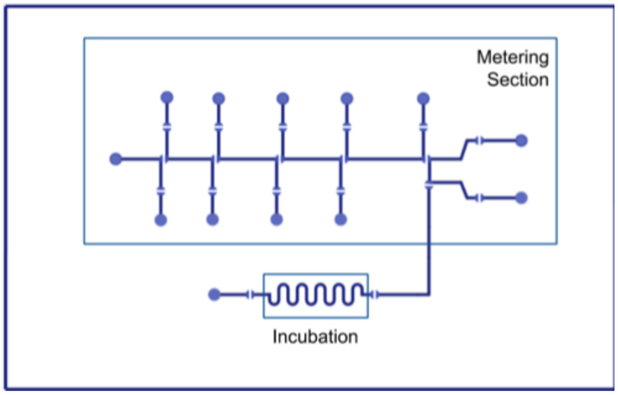

This microfluidic chip is designed to perform ligation. T4 DNA Ligase Buffer, vector DNA, insert DNA, water, and T4 DNA Ligase are metered on the chip and are then mixed together. The solution then undergoes incubation, after which the recombinant DNA solution can pipetted out from the chip and used in further molecular cloning procedures.

10.3.2 Design Layers

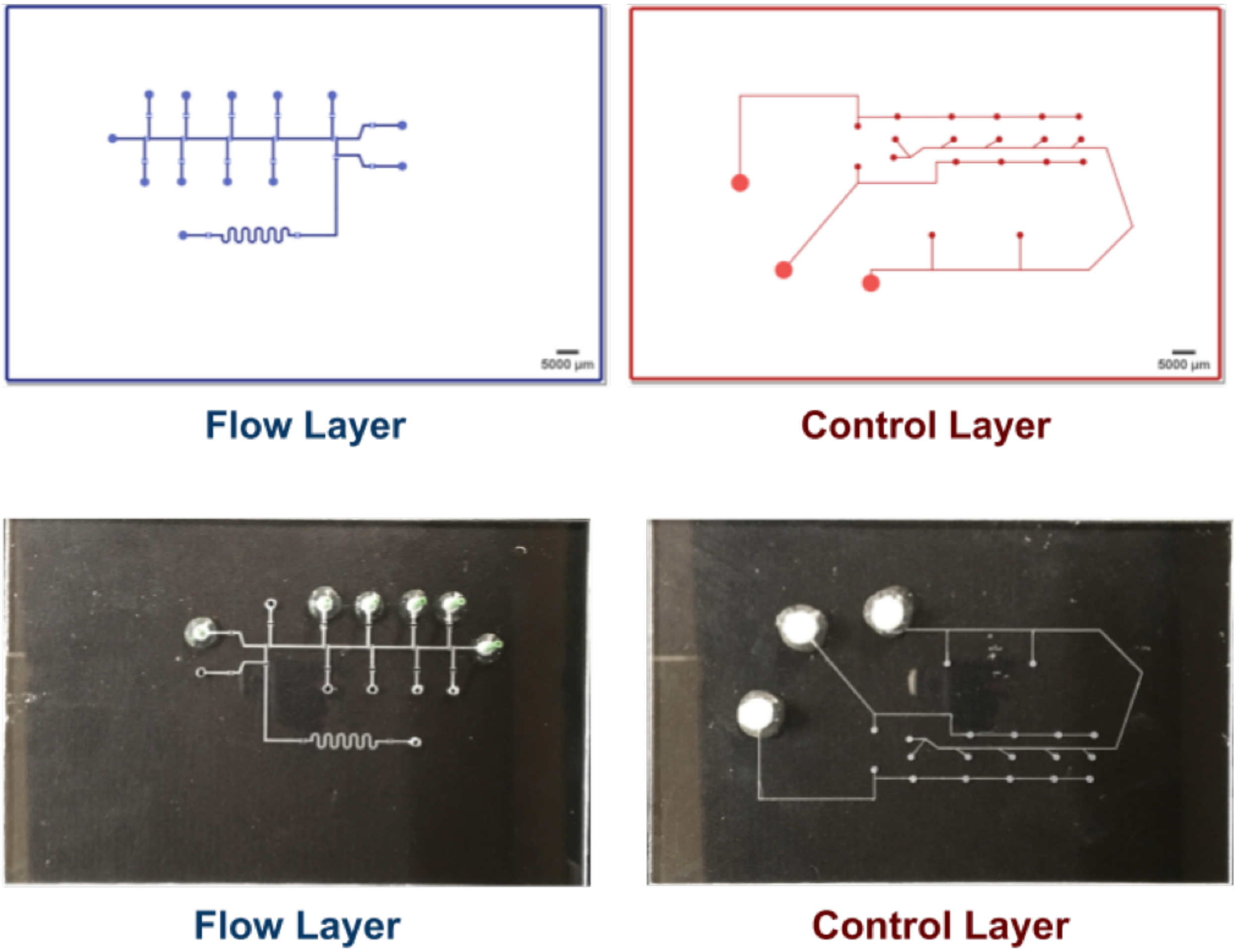

10.3.3 Inputs and Outputs

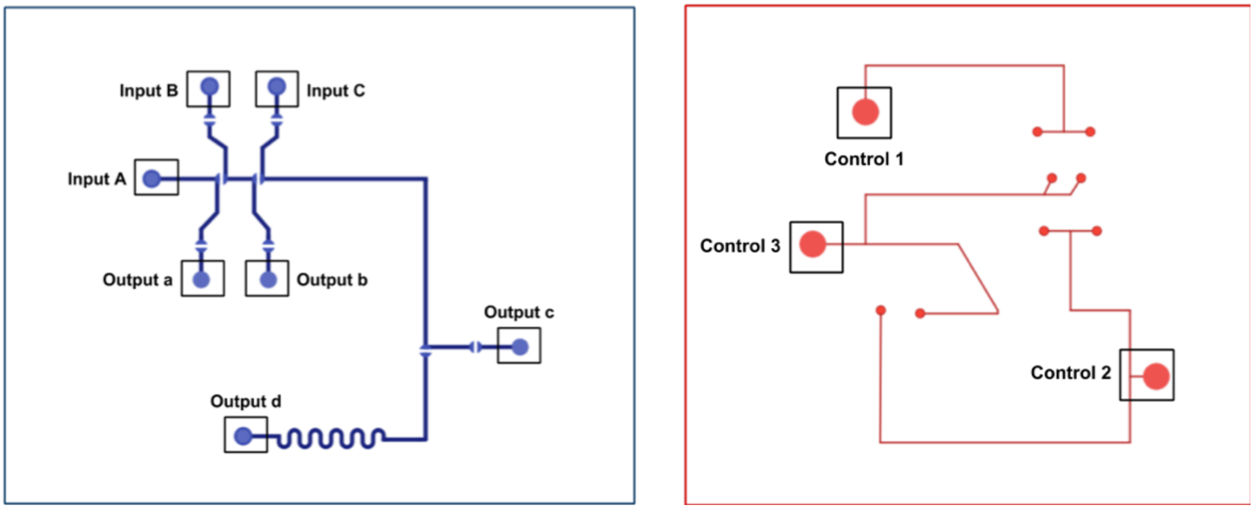

Name	Liquid	Flow Rate
A	Mineral Oil	0.5 mL/hour
B	T4 DNA Ligase Buffer	0.5 mL/hour
C	Vector DNA	0.5 mL/hour
D	Insert DNA	0.5 mL/hour
E	Nuclease-free water	0.5 mL/hour
F	T4 DNA Ligase	0.5 mL/hour

Table 10: Inputs

Name	Liquid
A	Excess Mineral Oil
B	Excess T4 DNA Ligase Buffer
C	Excess Vector DNA
D	Excess Insert DNA
E	Excess Nuclease-free water
F	Excess T4 DNA Ligase
G	Final Output

Table 11: Outputs

10.3.4 Protocol

Setup

1. Prepare 10 syringes
   (a) 5 filled with different colored water
   (b) 1 filled with mineral oil
   (c) 3 empty 10 mL control syringes
2. Attach your syringe containing mineral oil to Input A
3. Attach the remaining colored water syringes to inputs B-F
4. Attach your waste output tubing to Outputs a-f; this liquid will be excess fluid
5. Attach your output tubing to Output g; this tube should connect to an Eppendorf or other small collection receptacle
6. Attach three separate control syringes to Control 1, Control 2, and Control 3
7. If a heating element is being utilized, ensure this element is turned on and at the correct temperature

Execution

1. Open Control 1, then Control 2; you should feel significant resistance while you open these control valves
2. Begin flowing your mineral oil and all colored waters at flow rates of 0.5 mL/hour each
3. Once the mineral oil and colored waters have filled their metering sections and have begun filling the output port, ensure all of their syringe pumps have been turned off
4. Close Control 1, pause, then close Control 2
5. Open Control 3
6. Flow the mineral oil again at 0.5 mL/hour
7. The oil will push and mix the two colored waters
8. When the colored water has moved into the mixer and has begun crossing the final valve, turn off the mineral oil syringe pump
9. Close Control 3
10. Incubate for the required amount of time, depending on the specific protocol
11. Open Control 3
12. Flow the mineral oil again at 0.5 mL/hour until all the colored liquid has been pushed out
13. Collect all of the output colored liquid in your designated receptacle

Sequential Instructions All time units are in s.

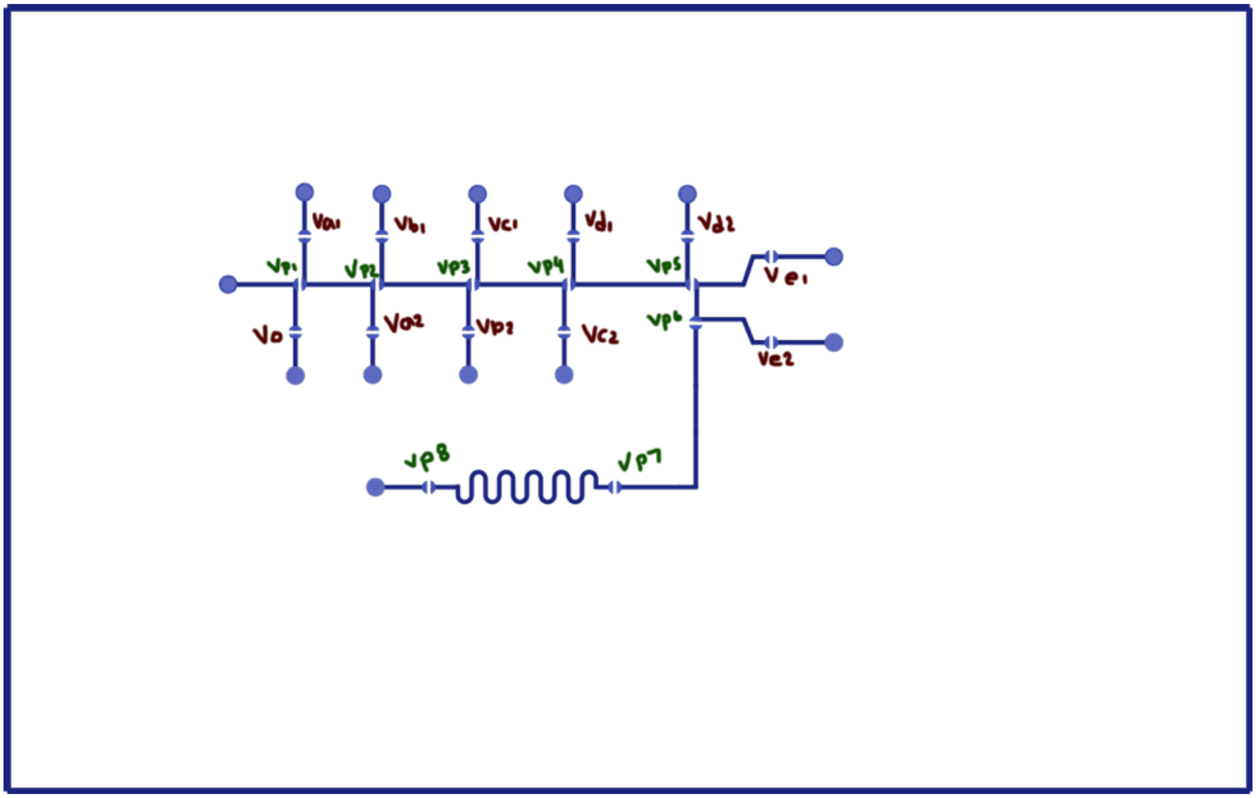

Figure 19: Sketch annotating the component names used for the device

```
SET va1 OPEN
SET vb1 OPEN
SET vc1 OPEN
SET vd1 OPEN
SET ve1 OPEN
SET va2 OPEN
SET vb2 OPEN
SET vc2 OPEN
SET vd2 OPEN
SET ve2 OPEN
WAIT 100
SET va1 CLOSE
SET vb1 CLOSE
SET vc1 CLOSE
SET vd1 CLOSE
SET ve1 CLOSE
WAIT 2
SET va2 CLOSE
SET vb2 CLOSE
SET vc2 CLOSE
SET vd2 CLOSE
```

```
SET ve2 CLOSE
WAIT 2
SET vp1 OPEN
SET vp2 OPEN
SET vp3 OPEN
SET vp4 OPEN
SET vp5 OPEN
SET vp6 OPEN
SET vp7 OPEN
SET vp8 OPEN
WAIT 250
SET vp1 CLOSE
SET vp2 CLOSE
SET vp3 CLOSE
SET vp4 CLOSE
SET vp5 CLOSE
SET vp6 CLOSE
SET vp7 CLOSE
SET vp8 CLOSE
WAIT 3600
SET vp1 OPEN
SET vp2 OPEN
SET vp3 OPEN
SET vp4 OPEN
SET vp5 OPEN
SET vp6 OPEN
SET vp7 OPEN
SET vp8 OPEN
WAIT 120
SET vp1 CLOSE
SET vp2 CLOSE
SET vp3 CLOSE
SET vp4 CLOSE
SET vp5 CLOSE
SET vp6 CLOSE
SET vp7 CLOSE
SET vp8 CLOSE
```

10.4 DNA Digest

DNA digestion, or DNA fragmentation, is typically performed before analysis of the DNA sequence, or in order to perform further protocols. Restriction enzymes are mixed and then incubated with DNA in a buffer solution. This yields DNA fragments cleaved at specific sites according to the enzymes used.

10.4.1 Chip Design

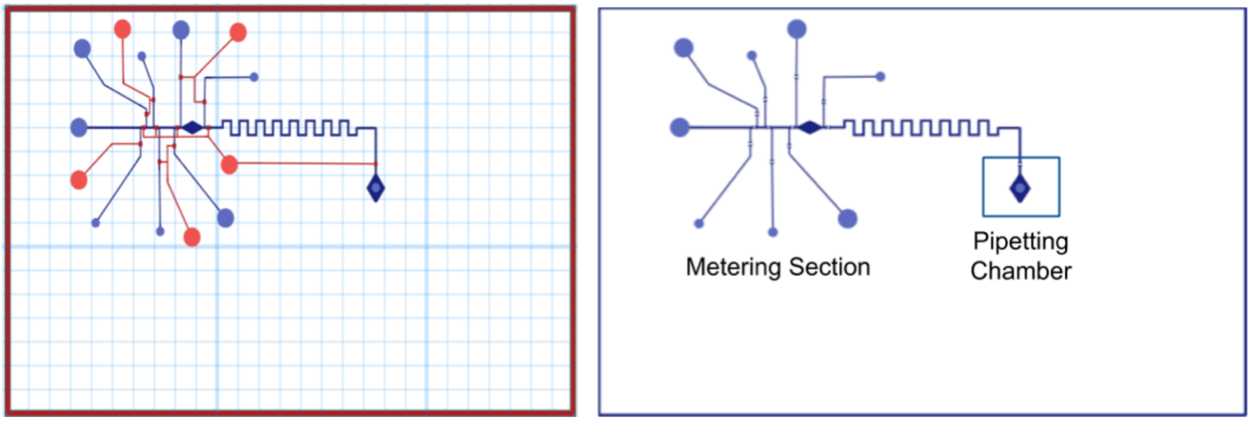

This microfluidic chip is designed to perform ligation. T4 DNA Ligase Buffer, vector DNA, insert DNA, water, and T4 DNA Ligase are metered on the chip and are then mixed together. The solution then undergoes incubation, after which the recombinant DNA solution can pipetted out from the chip and used in further molecular cloning procedures.

10.4.2 Design Layers

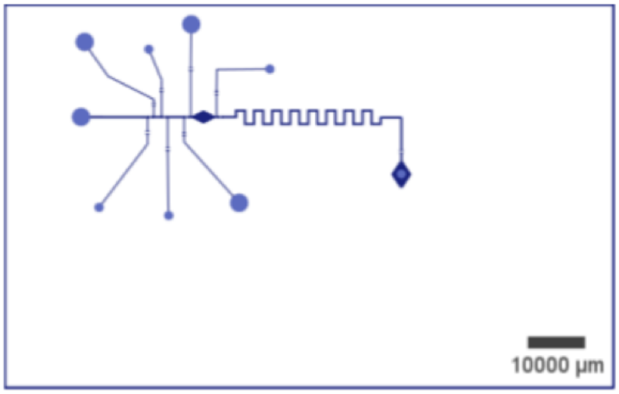

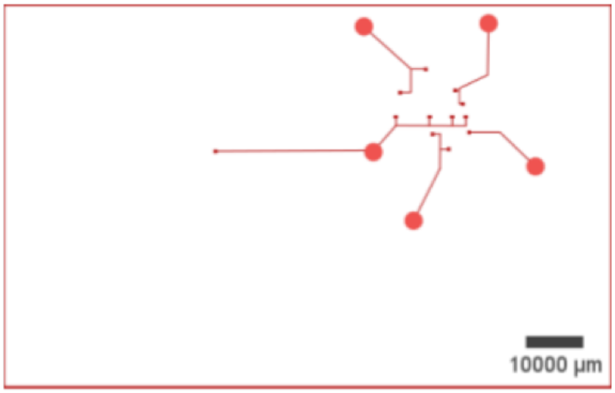

Flow Layer

Control Layer

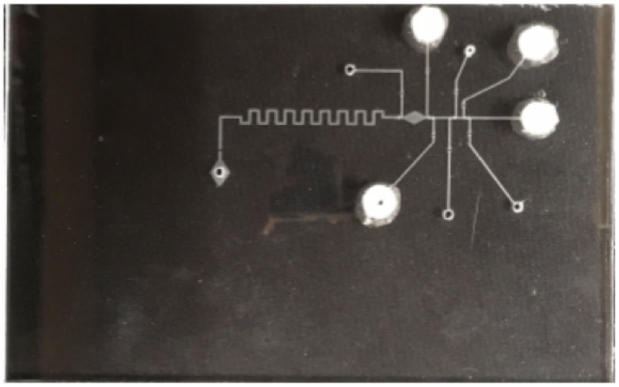

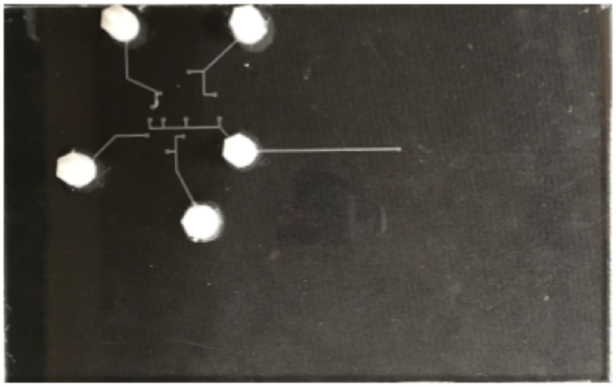

Flow Layer

Control Layer

10.4.3 Inputs and Outputs

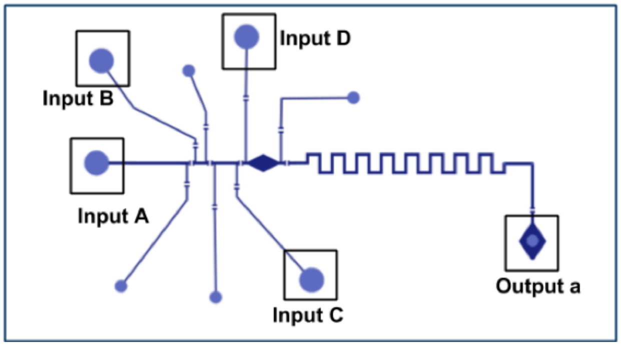

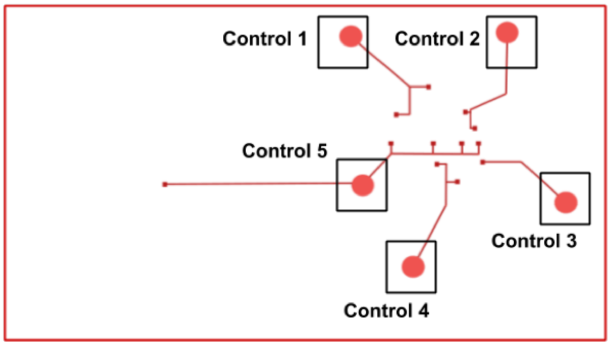

A	Mineral Oil	0.5 mL/hour
B	DNA Suspension	0.5 mL/hour
C	Buffer and Enzyme Solution	0.5 mL/hour
D	Water	0.5 mL/hour

Table 12: Inputs

Name	Liquid
A	DNA Fragments in Suspension

Table 13: Outputs

10.4.4 Protocol

Setup

1. Prepare 8 syringes
   (a) 3 containing coloured water
   (b) 1 filled with mineral oil
   (c) 4 control syringes
2. Attach the syringes containing colored water to inputs B,C and D
3. Attach your output tubing to Output a; this tube should connect to an eppendorf or other small collection receptacle
4. Attach two separate control syringes to Control 1 and Control 2

Execution

1. Open Control 1, Control 2, Control 3 and Control 4
2. Begin flowing inputs A, B, C and D at 0.5 mL/hour
3. Halt each flow when its respective metered channel on the chip is filled, then close its corresponding Control line
4. Open Control 5 and begin flowing input A at 0.5 mL/hour again
5. When the oil is 1 cm away from crossing the final valve, halt the flow of input A
6. After 20 seconds, close Control 5
7. Leave the mixture to incubate for 1 hour
8. Use a pipette tip to break the seal over the incubation chamber and transfer the DNA digest

Sequential Instructions All time units are in *s*.

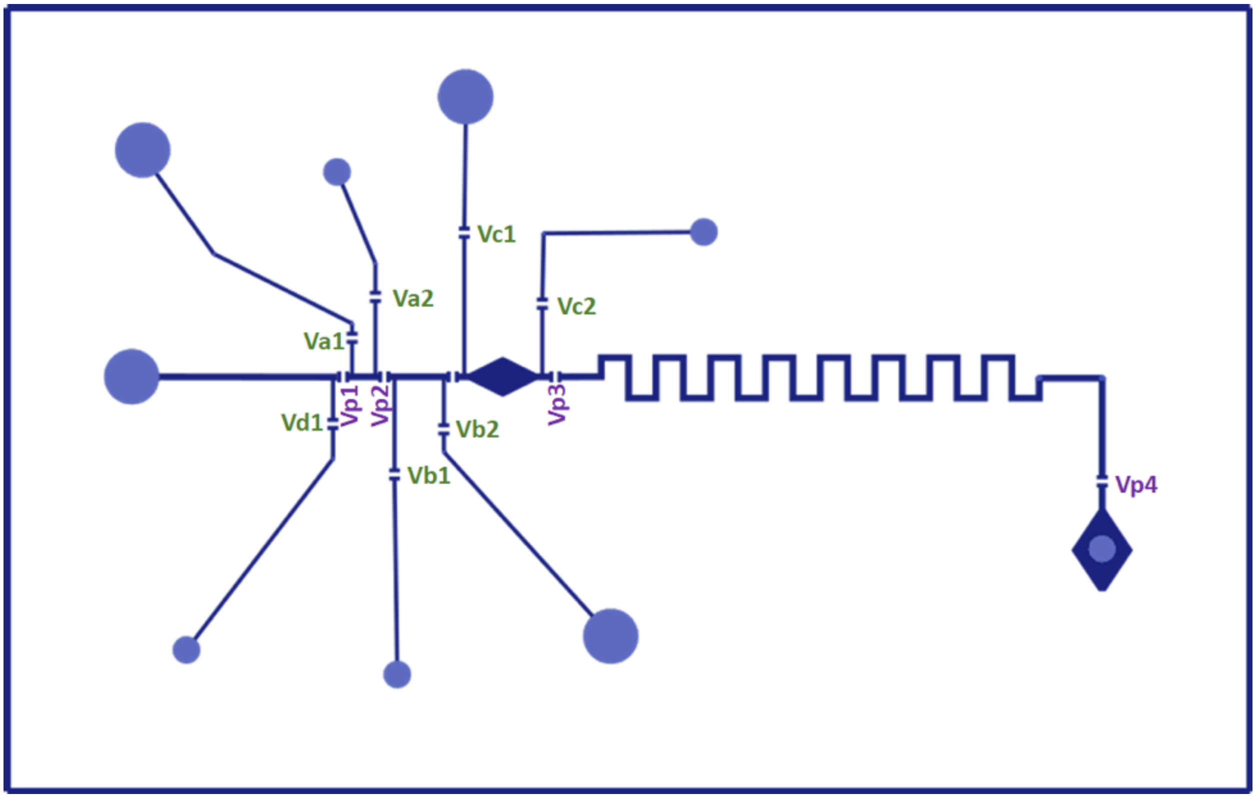

Figure 20: Sketch annotating the component names used for the device

```
SET Va1 OPEN
SET Va2 OPEN
SET Vb1 OPEN
SET Vb2 OPEN
SET Vc1 OPEN
SET Vc2 OPEN
SET Vd1 OPEN
WAIT 70
SET Va1 CLOSE
SET Vb1 CLOSE
WAIT 20
SET Va2 CLOSE
SET Vb2 CLOSE
WAIT 30
SET Vd1 CLOSE
WAIT 20
SET Vc1 CLOSE
SET Vc2 CLOSE
WAIT 3
SET Vp1 OPEN
SET Vp2 OPEN
```

```
SET Vp3 OPEN
WAIT 180
SET Vp1 CLOSE
WAIT 2
SET Vp2 CLOSE
WAIT 2
SET Vp3 CLOSE
```

10.5 Cell Lysis

The ability to sort cells by type or physical properties is a valuable tool in many synthetic biology labs. Prior to analysis or in order to perform specialised protocols, creating homogenous cell suspensions from a mixture is necessary. In addition to sorting cells, the removal of cell fragments, activated magnetic particles or unwanted debris through sorting also makes up a key part of purification protocols.

This device was created from the ground up by converting the chemical lysis protocol from the paper by Junkyu et al. [9]. First, the lysis buffer and the suspended cell are mixed using the micromixer which then is mixed with the neutralization, elution buffers and forwarded into the second mixer stage. Finally, the lysis mixture is separated using a magnetic separator stage as before the final product is extracted from the outlet on the bottom right of the chip.

10.5.1 Chip Design

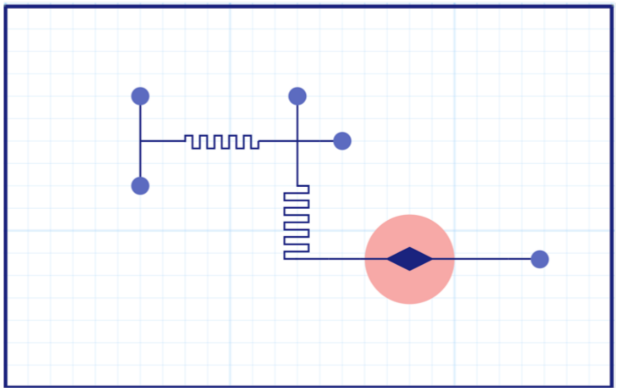

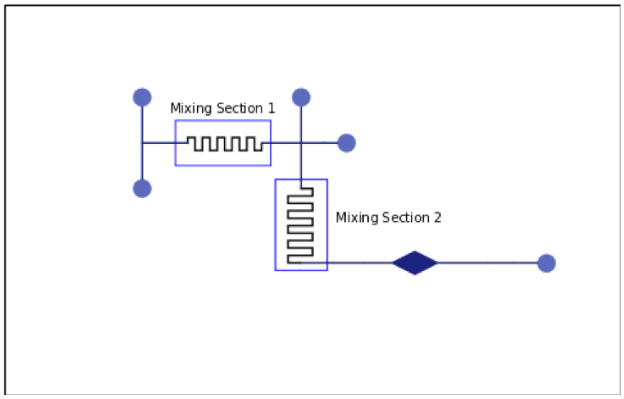

This microfluidic chip design carries out cell sorting as a cell suspension is passed through it. Cells are sorted based on size and pushed to the periphery of the channel. These cells are then carried away from the main solution through the two periphery outputs, and the cell-free solution can be collected from the central output.

10.5.2 Design Layers

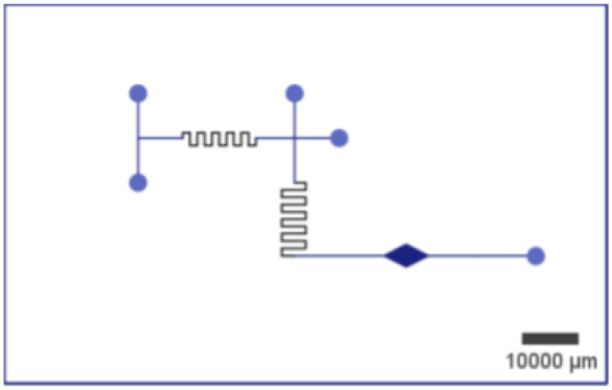

Flow Layer

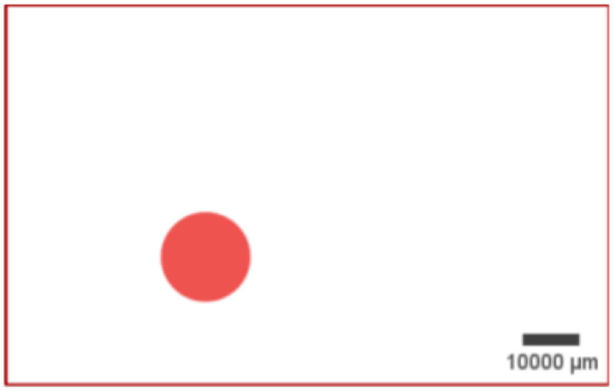

Control Layer

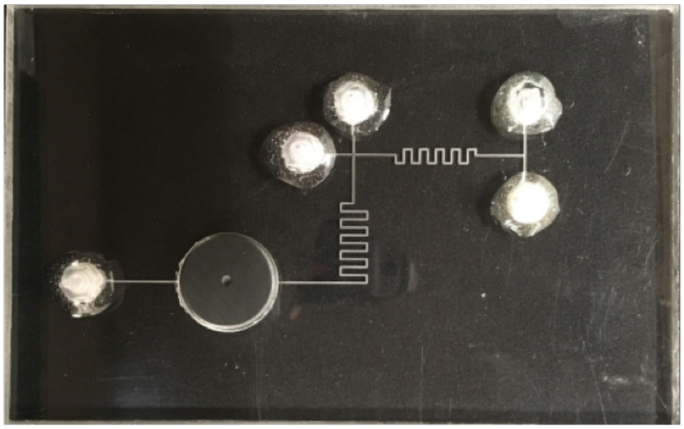

Flow Layer

10.5.3 Inputs and Outputs

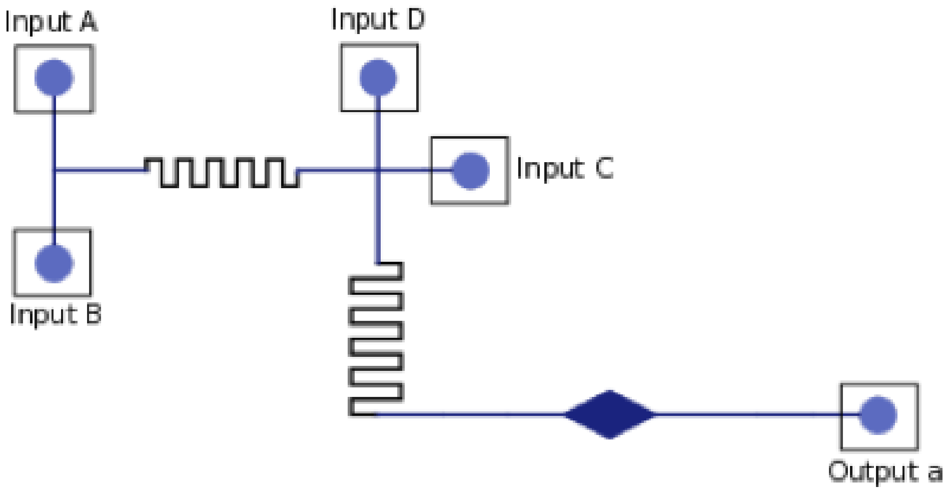

A	Lysing Buffer	3.0 mL/hour
B	Suspended Cells	3.0 mL/hour
C	Neutralization Buffer	6.0 mL/hour
D	Elution Buffer	3.0 mL/hour

Table 14: Inputs

Name	Liquid
A	Excess Buffer/Cells and DNA

Table 15: Outputs

10.5.4 Protocol

Setup

1. Prepare 4 syringes

    (a) 1 filled with yellow colored water

(b) 1 filled with blue colored water
(c) 1 filled with purple colored water
(d) 1 filled with black colored water

2. Attach your syringe containing yellow colored water to Input A
3. Attach your syringe containing blue colored water to Input B
4. Attach your syringe containing purple colored water to Input C
5. Attach your syringe containing black colored water to Input D
6. Attach your waste output tubing to Outputs a; this liquid will be excess fluid or fluid containing DNA

Execution

1. Begin flowing the cell suspension into the chip at a rate of 0.3 mL/hour
2. Allow the fluid to completely fill the channels and flow out of the three outputs
3. Continue to flow liquid through the channels for thirty seconds, pause the syringe pump and dispose of the original eppendorf tubes
4. Attach new eppendorf tubes to the outputs and restart the fluid flow

Sequential Instructions Since device has no valves on the design, the sequential instructions set the flow rates at different ports rather than set the valves to OPEN/CLOSE. All the flow rate units shown here are in $\mu L/hr$. All time units are in s.

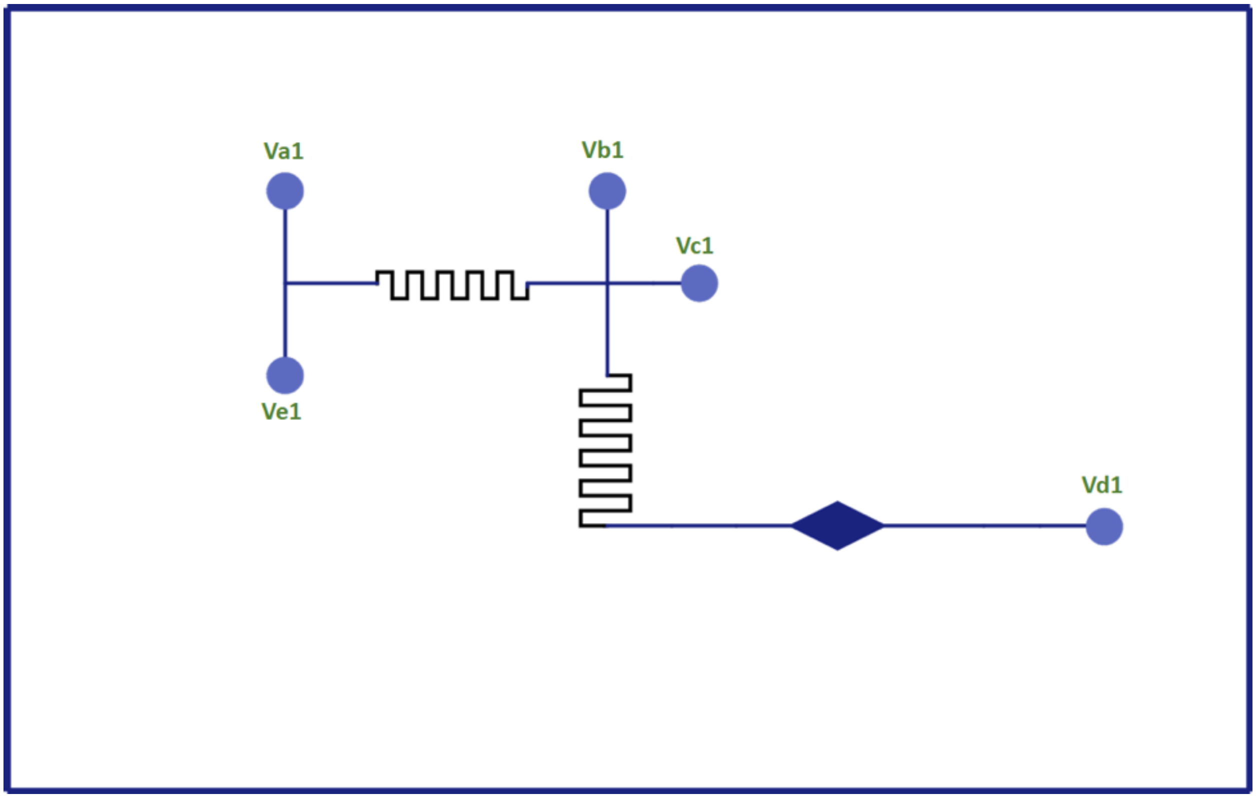

Figure 21: Sketch annotating the component names used for the device

```
SET va1 3000
SET ve1 3000
WAIT 40
SET vc1 6000
WAIT 120
SET Va1 0
SET Ve1 0
SET Vc1 0
WAIT 2
SET Vb1 3000
WAIT 120
SET Vb1 0
```

11 Platform Extensibility Demonstrations

11.1 Overview

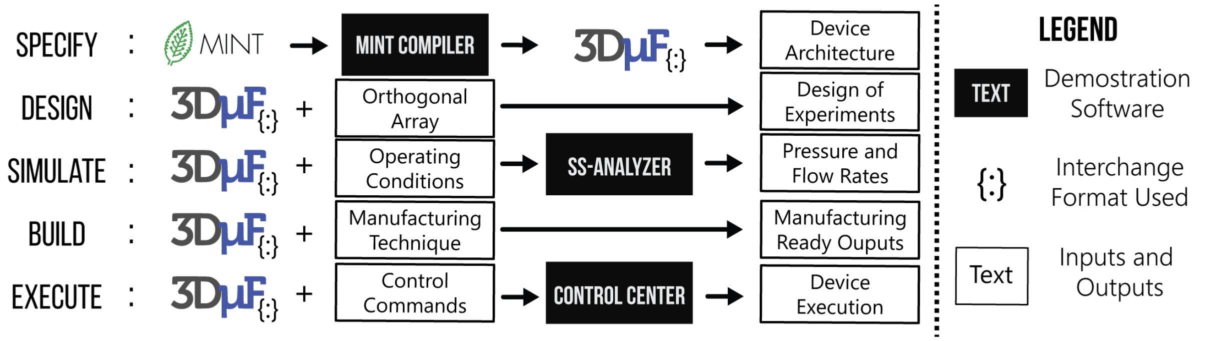

Figure 22: Overview of how the different stages of microfluidic design can be extended using 3DµF as platform. The figure also highlights the different demonstration tools developed to demonstrate the capability designed for each one of these applications.

11.2 Specify - Support for High-Level Device Descriptions

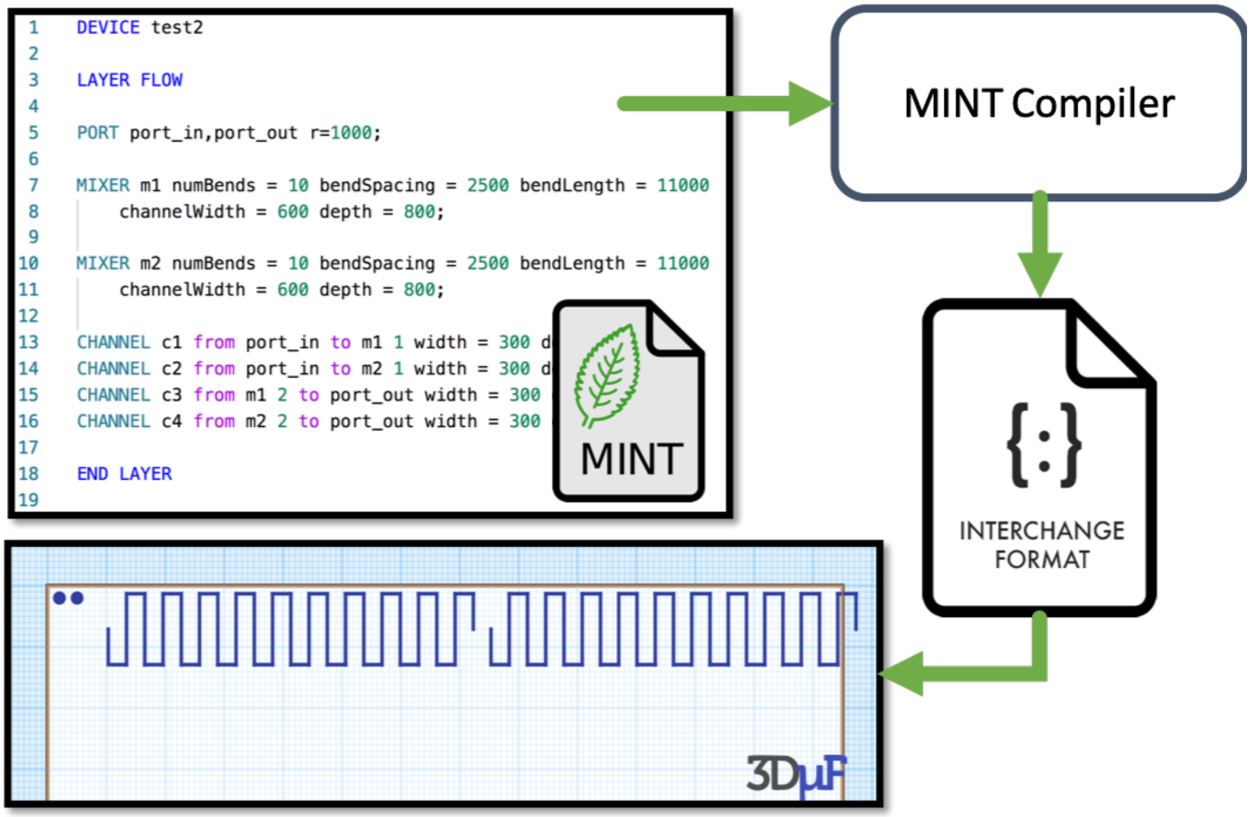

Figure 23: Designs created in MINT can be compiled to the same JSON format representing both the unfinished and finished layouts of the microfluidic device.

11.2.1 Explanation of MINT Syntax

MINT is a microfluidic hardware description language used for describing microfluidic circuits and devices. Just like any other Hardware Description Language like Verilog or VHDL, MINT aims to help microfluidic designers leverage the use of CAD tools by allowing them to describe complex architectures in a human and machine-readable format. By using MINT descriptions of the hardware. The designers can easily share and tweak the circuit and component parameters and speed up the entire experimental process. The MINT/netlist file is a plain text file that has the extension '.uf' (eg. device.uf).

The basic structure of the netlist as follows:

1. Instantiate the type of device and the device name.
2. Declare each layer present in the device.
3. Within each layer declare the components and channels on that layer.

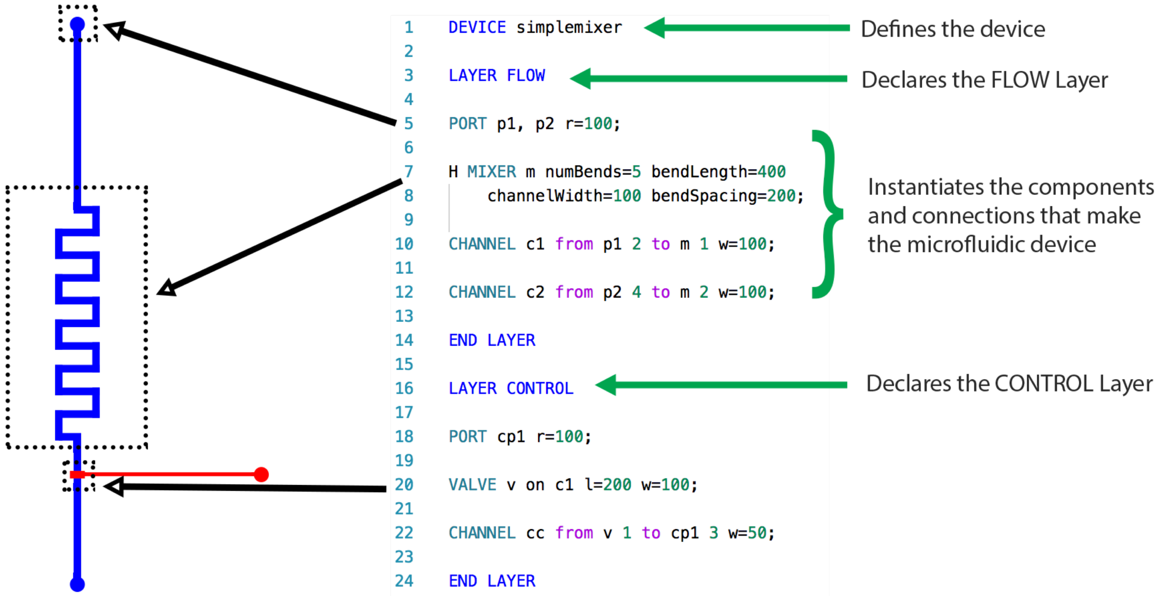

Figure 24: Example of a simple device described using MINT and how it relates to the rest of the design. **NOTE:** This the example shown here corresponds to a design that is completely laid out. Currently 3DµF does not automatically generate the layout of the device description.

Example of the minimal MINT device A step-by-step tutorial for creating MINT devices is available at `https://github.com/CIDARLAB/mint/wiki/Netlist-Rules-and-Examples`.

11.2.2 Demonstration

```
DEVICE test

LAYER FLOW

PORT port_in1, port_in2, port_out portRadius=1000;

MIXER m1 numBends = 5 bendSpacing = 1250 bendLength = 5500
    channelWidth = 300 depth = 400;

CHANNEL c1 from port_in1 to m1 1 width = 300 depth = 400;
CHANNEL c2 from port_in2 to m1 1 width = 300 depth = 400;
CHANNEL c3 from m1 2 to port_out width = 300 depth = 400;

END LAYER

```

(a) MINT example used to demonstrate Specify.

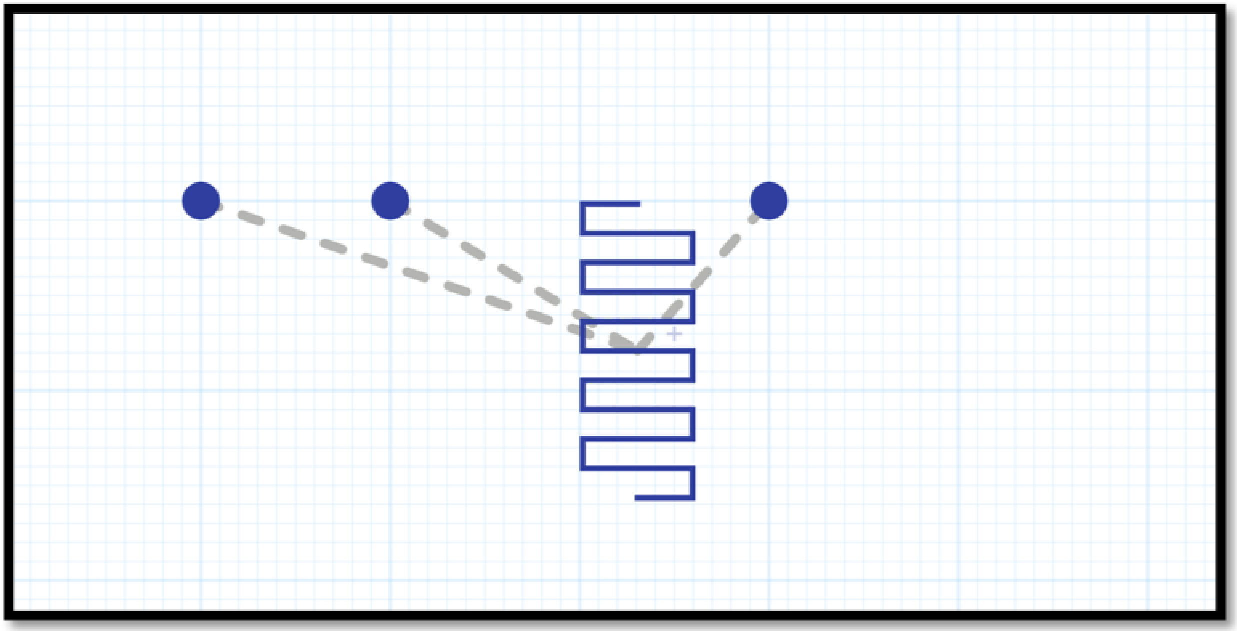

(b) A Rat's Nest visualization used to represent the unrouted connections in the designs.

Figure 25: MINT example used to demonstrate the 3DμF's ability to understand designs created using a hardware description language and the visualization that can represent the device design.

Here we have an example of the tool being able to import designs described using the language MINT, a hardware description language. In order to understand the devices specified in MINT[7], we first compile the MINT design using the MINT compiler generate a JSON file which can then be imported by 3DμF. By leveraging both the MINT standard component library and standard interchange format defined by Parchmint[2] 3DμF can allow the user to generate the layout of the device abstractly specified in hardware description languages as seen in Figure 25.

11.2.3 Resources

- MINT Wiki - `https://github.com/CIDARLAB/MINT/wiki`
- MINT Technology Files - `https://github.com/CIDARLAB/MINT/tree/REFRESH`
- MINT Compiler Github Repository - `https://github.com/CIDARLAB/miniFluigi`

11.3 Design - Support for Design of Experiments

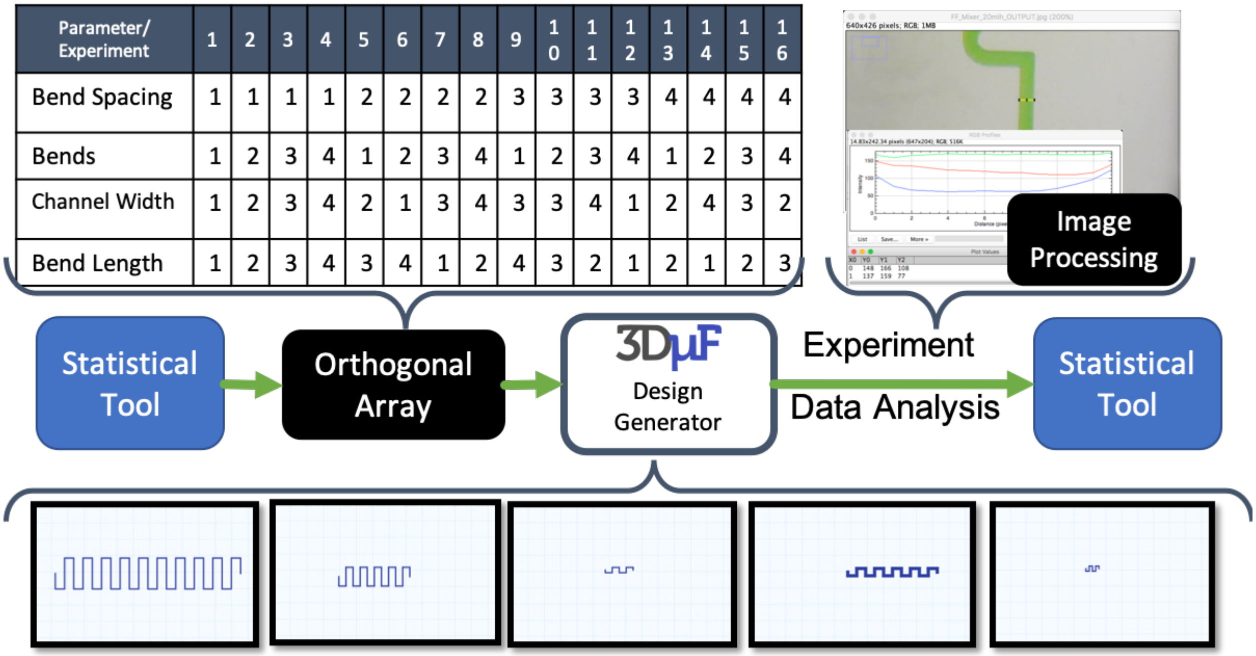

Figure 26: This figure illustrates how the design process takes place.

11.3.1 Example - DOE to improve Mixer Efficiency

The mixing of two fluids is something that can often be qualitatively observed, however in order to properly execute an experiment, specific degrees of mixing must be achieved. In order to evaluate the degree of which fluids are being mixed in a mixer primitive a mixer efficiency test is needed to be performed. This quantitative test is broken down into a two-part process where the first step is *Image Processing* and *Efficiency Calculation*.

Image Processing After running fluid through the mixer, pictures need to be taken at the regions before entering the primitive and after it exits the primitive. In order to obtain RGB data from these images, image processing software such as ImageJ need to be implemented. Using ImageJ a 1-pixel box is created that spans the length of the channel. The two crucial pieces of data that need to be obtained using this box are the average RGB value over that area and the RGB value at the outer edge of the channel. After collecting this data, we can move on to calculate the mixing efficiency.

Efficiency Calculation The efficiency of the mixing itself can be measured using Equation (5). Once this value is obtained, the channel primitive can be measured to see if it is

suitable to perform the needed function.

$$\gamma = 1 - 2\left[\frac{\int (RGB - RGB_{avg})dL}{\int dL}\right] \tag{5}$$

11.3.2 Generate Orthogonal Array

In order to perform design of experiments, the user first has to use statistical software such as Minitab www.minitab.com to generate the orthogonal array and to process the data generated during the experiment. Figure 27 shows how an orthogonal array can be generated and save subsequently saved as a .csv/.xlsx file.

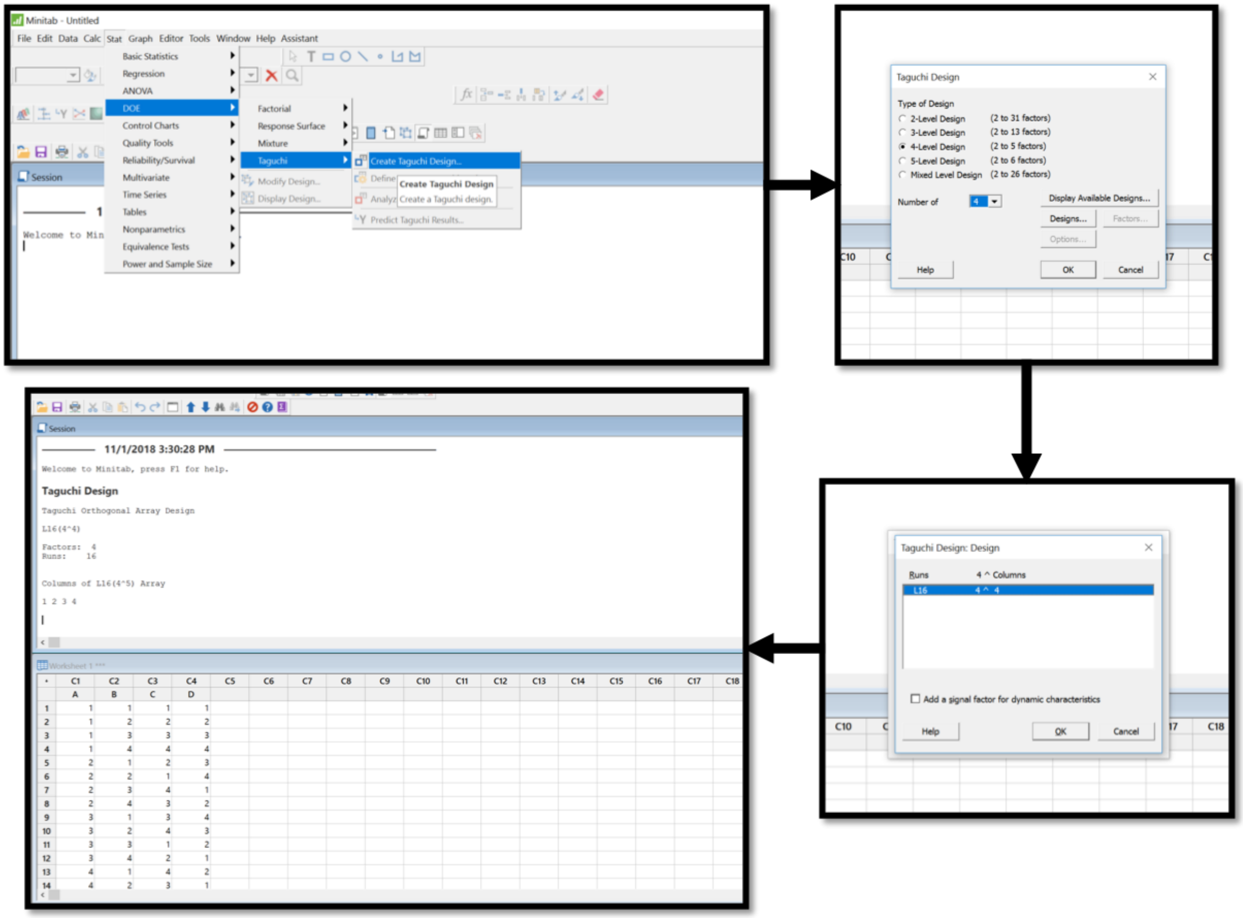

Figure 27: Minitab is a statistical tool that can be used to generate the orthogonal design array that will be used to generate the design of experiments.

11.3.3 Demonstration

Since the DOE designer is still experimental, the user needs to execute a command in the browser's developer console to activate the UI necessary to generate the DOE variations.

The command that needs to be entered into the console is as follows:

```
Registry.viewManager.taguchiDesigner.openDialog("ComponentName");
```

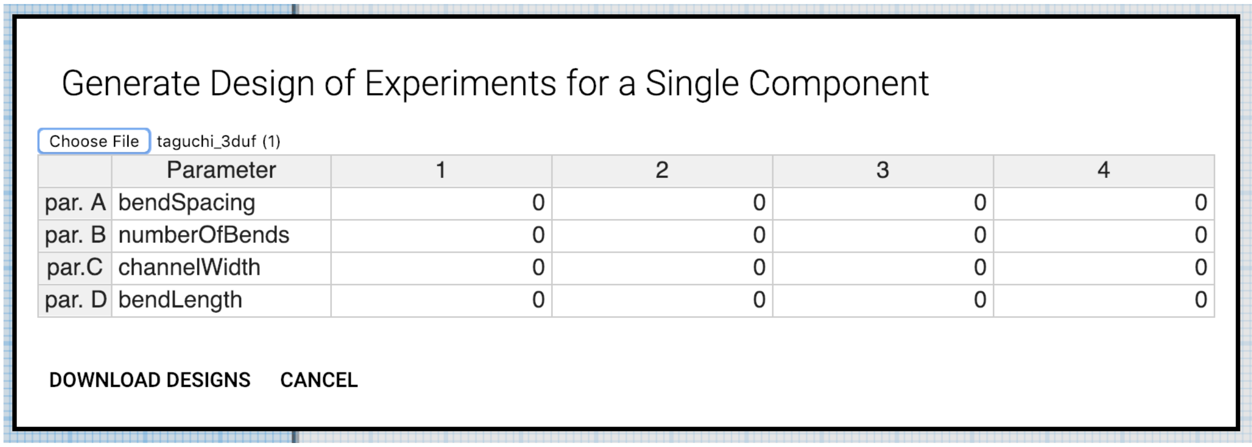

Figure 28: Screenshot of the Taguchi Designer UI Concept built into 3DμF.

First, the user has to select the orthogonal array file in order to populate the tables with the parameter and level options as seen in Figure 28. The user can download the design variations by clicking the *DOWNLOAD DESIGNS* button.

11.3.4 Resources

- Minitab - www.minitab.com
- Demonstration files and Video - Supporting Materials

11.4 Simulate - Support for Network Characterization

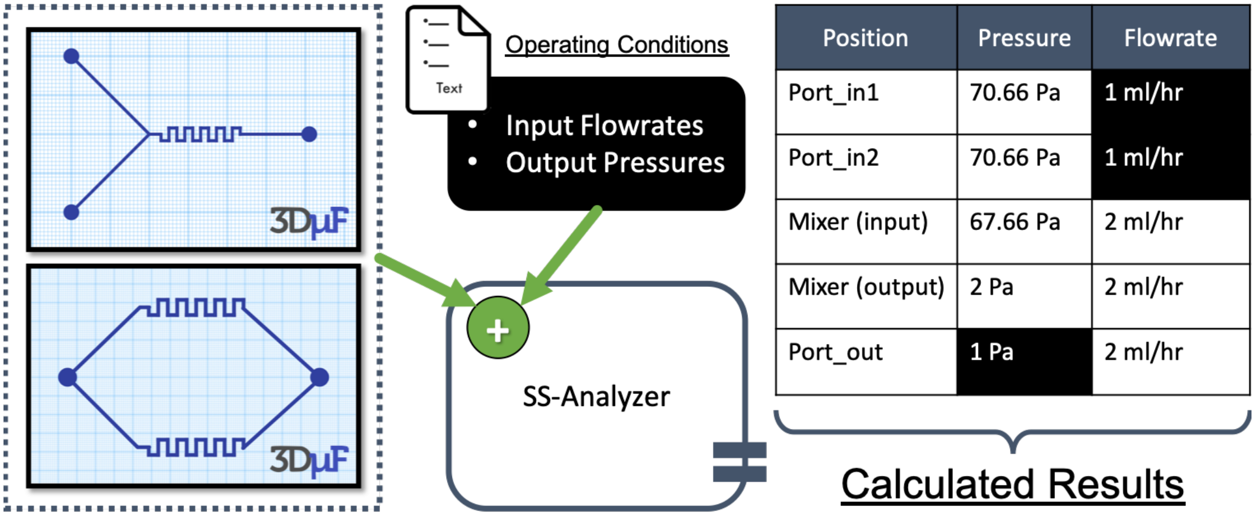

Figure 29: Diagram that shows an example demonstration of the simulate workflow.

11.4.1 Demonstration

The demonstration for the analysis tool only supports *MIXER*s and 1-1 *CONNECTIONS* currently. The tool ingests the design file and converts the microfluidic network into an equivalent electrical model, which is then solved by constructing a series of pressure, flow-rate equations[17].

The solver can be run by executing the following command on the command line.

```
python tool.py designFile.json -i config.txt
```

Each line in the config file consists of 3 parameters, *component name*, *input/output state* and *pressure/flowrate value*. An example is given below:

```
port_in1, IN, 1
port_in2, IN, 1
port_out, OUT, 101325
```

11.4.2 Resources

- Github Repository for SS-Analyzer - https://github.com/CIDARLAB/SS-Analyzer

11.5 Build - Support for Fabrication

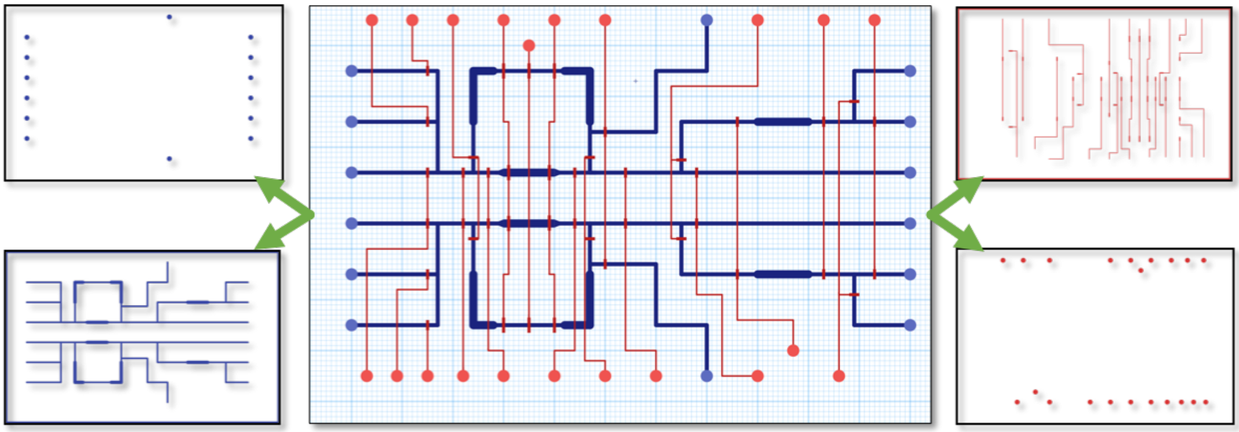

Figure 30: Every design created in 3DμF can be automatically separated into individual layers that can be manufactured easily.

11.5.1 Design For Manufacturing Classes

The use of Design For Manufacturing (DFM) classes to annotate each of the features generated by 3DμF helps ensure that the automation of the manufacturing ready outputs of the tool. Each of the DFM classes is described in Table 16.

DFM Class	Description	MFG Process	Generated Output
XY	2D Profile	Lithography, Micromilling,	SVG
XYZ	3D Solid Object	3D Printing, Micromilling	STL
Z	2D Drill Profile	3D Printing, Micromilling	SVG
EDGE	Outline Profile	Any	SVG

Table 16: Description of the Design for Manufacturing classes used in 3DμF.

11.5.2 Manufacturing Output Generation

In order to generate outputs for manufacturing, 3DμF processes each feature based on the Z-Axis depth and the associated DFM class to generate the required design output described

by table 16. The algorithm used to process the *Feature* objects is described in Algorithm 1.

```
Data: List of Geometric Features - F
Result: Manufacturing Outputs - M
intialize depthMap, solidObjectList, drillList, bordersList , M;
for feature f in F do
    if f.DFMClass = "XY" then
        featureList;
        depth ← f.depth;
        if depthMap.keyexists(depth) then
            featureList ← depthMap.value(depth);
        end
        else
            initialize featureList;
            depthMap[depth] ← featureList;
        end
        featureList.append(f);
    end
    else if f.DFMClass = "XYZ" then
        solidObjectList.append(f);
    end
    else if f.DFMClass = "Z" then
        drillList.append(f);
    end
    else if f.DFMClass = "EDGE" then
        bordersList.append(f);
    end
    M.layers ← depthMap.values;
    M.borders ← bordersList;
    M.3Dobjects ← solidObjectList;
    M.drills ← drillList;
end
```

Algorithm 1: Manufacturing Output Generation

11.5.3 Demonstration

The demonstration available by pressing the *CNC* output download button on the tool at http://3duf.org

11.5.4 Resources

- OtherMill Micromilling Tool Library for Polycarbonate - https://github.com/CIDARLAB/Makerfluidics/

11.6 Execute - Support for Valve Control Programability

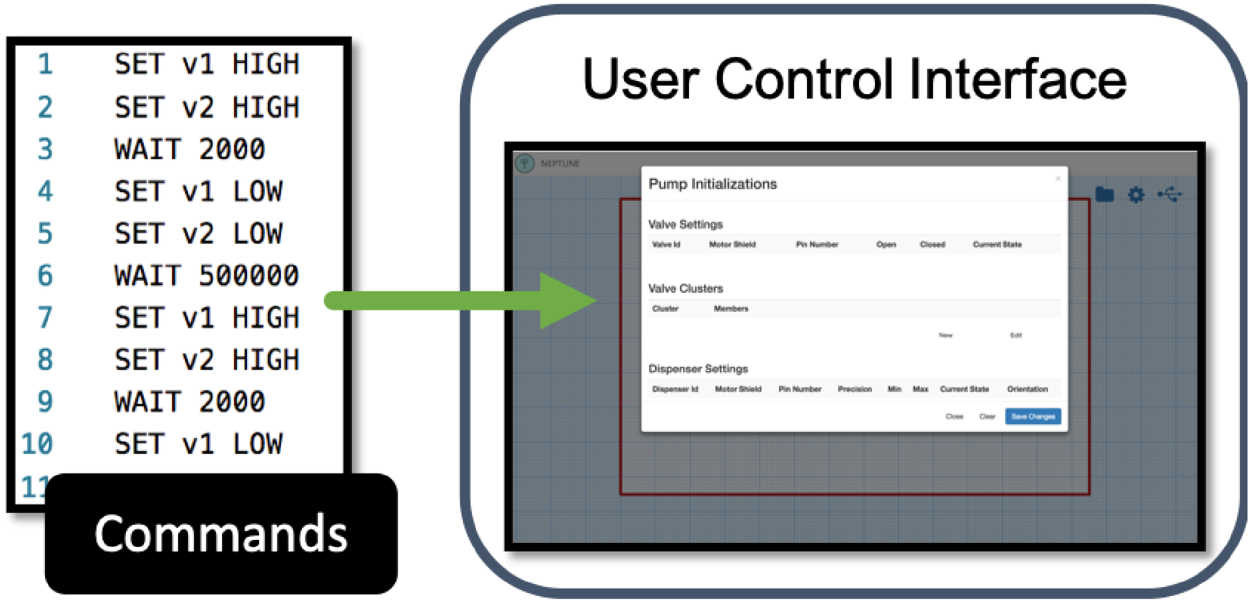

Figure 31: Screenshot of the Control Software UI that can execute instructions to actuate valves on the microfluidic device.

One of the challenges in deploying microfluidics into the research environment is realizing the execution of the microfluidic devices. Different vendors, labs usually opt for different hardware, programming languages to program the microfluidic devices. This becomes far more problematic because there is no automated mechanism to bind functionality to the microfluidic design. Designs created using 3DµF can be read by control automation tools where each of the components seen in the canvas are identified and can be mapped back to the code that describes the behavior of the device. The following demonstration shows an environment that can execute instructions for a simple device that directs the fluids to two different ports as seen in Figure 32. The demo software is available at https://cidarlab.github.com/CC.

11.6.1 Demonstration

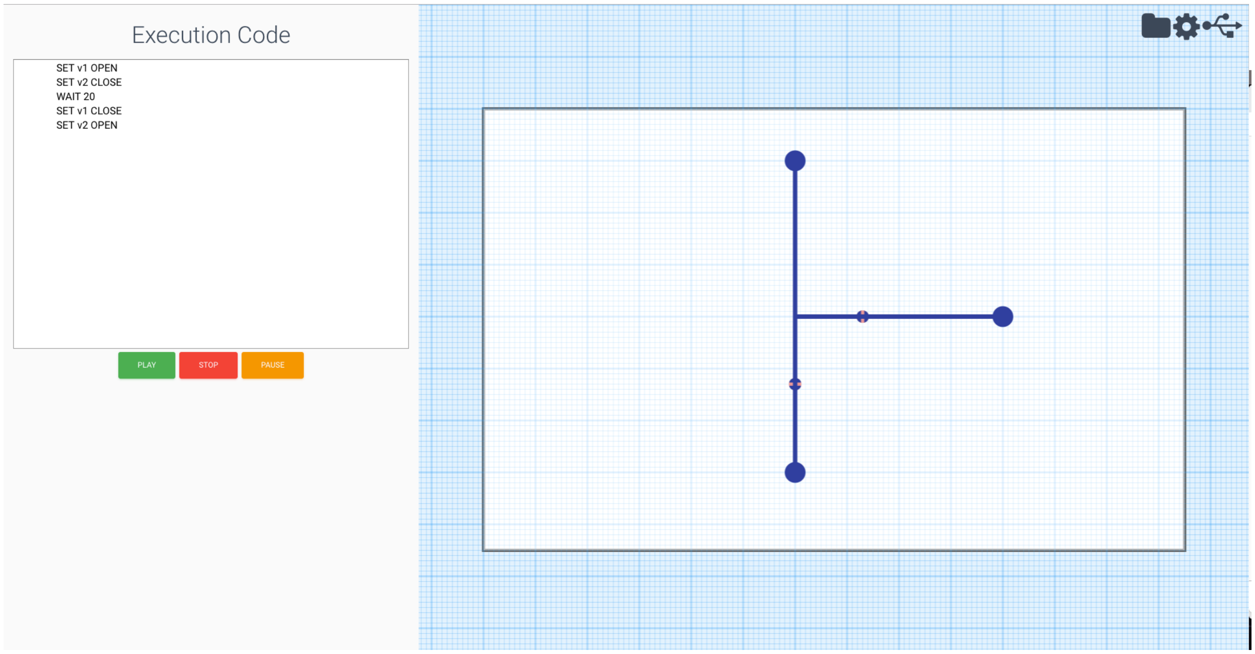

Figure 32: Demonstration software for the control UI that can be used to visualize execution of the microfluidic device.

The demonstration software utilizes 3DμF as the visualization engine for displaying the device. The software additionally has the ability to map each of the components to *hardware identifiers* and send messages to the hardware from the browser using *Peripheral Manager* as a communication middleware that establishes a channel between the browser and the hardware connected to the computer. **NOTE:** CC is active research project and is currently under development.

11.6.2 Sequential Command Examples

In order to demonstrate what valve sequences would look like for devices implementing real life protocols, we include example valve sequences for each of the devices in Case Study 2 of the manuscript, these programs can be found in 10.5.4, 10.4.4, 10.2.4, 10.5.4.

11.6.3 Resources

- Control software Github repository - `https://github.com/CIDARLAB/CC`
- Browser-Hardware Communication Bridge Github repository - `https://github.com/CIDARLAB/Peripheral-Manager`
- Control software demo website - `https://cidarlab.github.io/CC/`

12 Makerfluidics Protocol - SVG Manipulation

In order to rapidly validate the functionality of the devices developed using 3DμF, a low cost a low-cost, rapid prototyping protocol called *Makerfluidics*[20] was used to fabricate and test all the devices shown in the paper. This manufacturing protocol uses polycarbonate as the primary substrate and takes advantage of commercially available, low-cost CNC mills[12] as the primary equipment to pattern the features into the substrate. The application of this manufacturing protocol reduces the cost of fabrication to approximately $6 per chip and reduces the time to 3 hours. 3DμF takes advantage of the layers, feature types and parameters of the geometries in the design to generate the SVG files, thus simplifying the fabrication process. We utilize the Othermill `http://bantamtool.com` and the packaged automated SVG-CAM generation tools to machine the microfluidic onto the substrate. Once the design is milled onto the polycarbonate substrate, the patterned substrates are then cleaned with ethanol and DI water. Finally, the polycarbonate layers are then assembled with a flexible PDMS (Polydimethylsiloxane) membrane sandwiched between them to act as the valve actuation membrane which is held together using binder clips and the device is vacuum sealed in a desiccator.

3DμF supports the automatic generation of the SVG files required for micro-milling a device using the Makerfluidics protocol. However, if the user wants to generate or understand the SVG requirements for micro-milling manually, this chapter provides a short overview of the procedure involved in preparing the SVG for the Othermill software.

12.1 Preparing the SVG

In order to fabricate the microfluidic design using the Othermill, the SVGs generated by 3DμF (unseparated)[7] need to be manually edited using a vector editing tool like Inkscape. The following steps need to be performed on each of the files in Inkscape:

1. Flip the CONTROL layer horizontally but leave the FLOW layer as is
2. Create new layers in the SVG file: **ports**, **cutout** and **design**.
3. Ungroup all the SVG objects.
4. Select all the borders and move them to the **cutout** layer.
5. Select all the ports and move them to the **ports** layer.
6. Toggle visibility for each of the layers and save separate SVGs for the design, cut out and ports.

[7] When generating the CNC mill outputs from 3DμF , the user can skip the preparation step and directly use the SVG generated by the tool without any preprocessing in inkscape

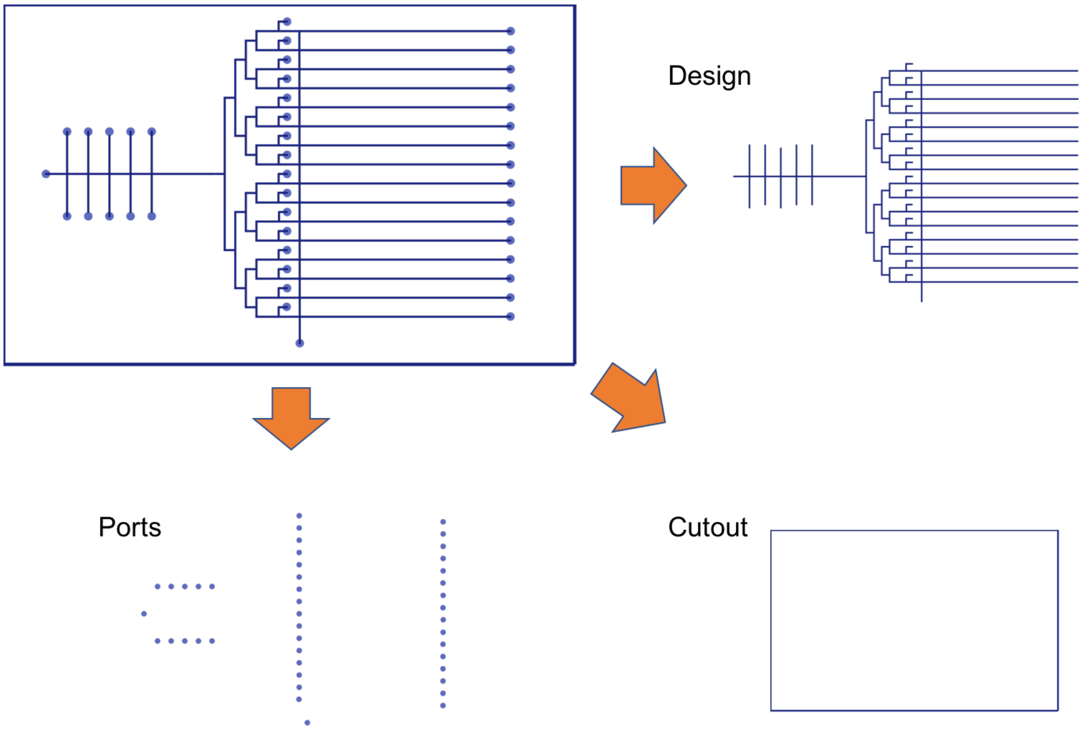

Figure 33: The ports, borders and the remainder of the design is separated into 3 different SVG files.

By following the above steps, we obtain 3 SVGs as seen in the fig. 33. The 3 SVGs can then be used to fabricate the ports, design and the cutout separately with different depth settings.

12.2 Tool Settings

In order to fabricate the chips in polycarbonate, we created a tool library that can be imported by the mill's CAM package. The tool library is available for download at Makerfluidics Github Repo. The designs provided with this paper will require the user to have the 1/8″, 1/16″, 1/64″ and 1/100″ flat endmills to be used during the fabrication process.

12.3 Tool-path Generation

We use the CAM package bundled with the mill's free control software Bantam Tools Software[8] to generate the tools paths. In order to generate the correct tools paths, the users need to follow the checklist given below:

[8]Formerly known as Otherplan

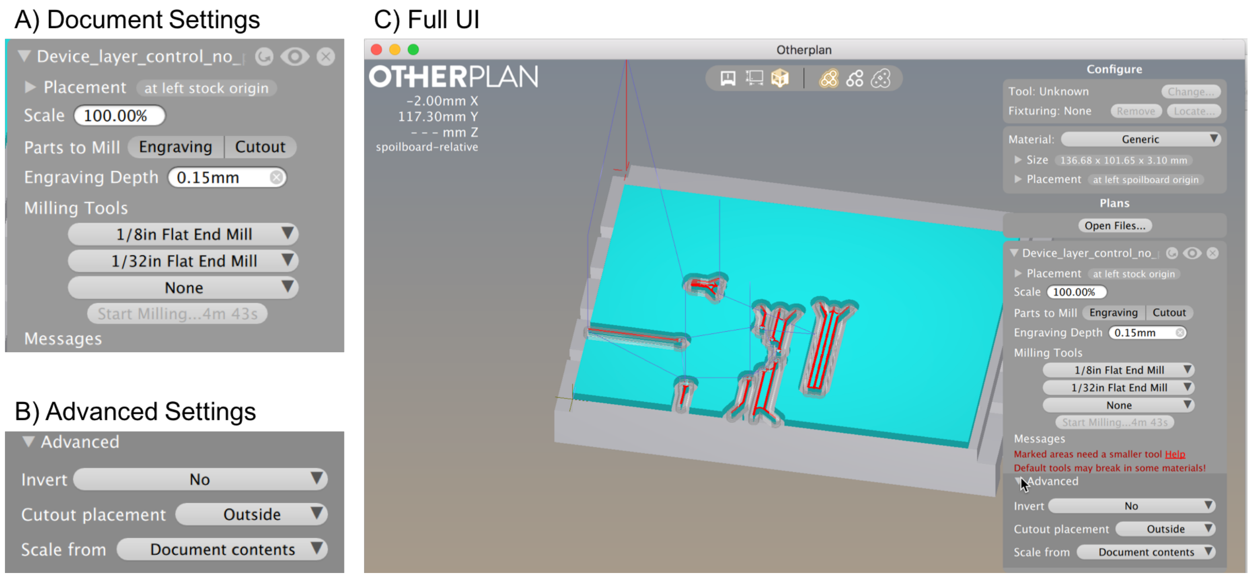

Figure 34: A) The document settings page where the tools and the primary fabrication details are set B) The advanced settings pane.

- ☐ Ensure that the device is scaled to 100% as show in fig. 34 - A
- ☐ Ensure that the device is scaled form Document Bounds as show in fig. 34 - B
- ☐ Ensure that the height of the stock is accurate[9]
- ☐ Deselect the "Cutout" option for each tool-path under Document Settings as seen in fig. 34 - A
- ☐ Set the desired Engraving Depth and End Mills for each layer under Documents Settings as shown in fig. 34 - A

On changing any of the settings, the tool automatically generates the new tool path that the mill will follow to fabricate the design. The areas highlighted in red on the design preview window are locations the tool cannot reach. These are typically sharp corners and features smaller than the tool's effective diameter. Once these areas, as in Figure 34, are either small enough not to be a concern or no longer highlighted in RED, the user should be able to fabricate the design by hitting the **Start Milling ...** button and following the instructions to on the screen.

NOTE: Additional resources on fabricating the devices using a CNC mill are available at MARS Video Tutorials.

[9]This can be done by placing a sheet of paper on the stock and lowering the endmill onto the paper until the paper feels some resistance from the endmill.

References

[1] Frederick K. Balagadde, Lingchong You, Carl L. Hansen, Frances H. Arnold, and Stephen R. Quake. Long-Term Monitoring of Bacteria Undergoing Programmed Population Control in a Microchemostat. *Science*, 309(5731):137–140, July 2005.

[2] B. Crites, R. Sanka, J. Lippai, J. McDaniel, P. Brisk, and D. Densmore. ParchMint: A Microfluidics Benchmark Suite. In *2018 IEEE International Symposium on Workload Characterization (IISWC)*, pages 78–79, September 2018.

[3] Tal Danino, Octavio Mondragon-Palomino, Lev Tsimring, and Jeff Hasty. A synchronized quorum of genetic clocks. *Nature*, 463(7279):326–330, January 2010.

[4] Stephan K. W. Dertinger, Daniel T. Chiu, Noo Li Jeon, and George M. Whitesides. Generation of Gradients Having Complex Shapes Using Microfluidic Networks. *Analytical Chemistry*, 73(6):1240–1246, March 2001.

[5] Cong Fang, Yanju Wang, Nam T. Vu, Wei-Yu Lin, Yao-Te Hsieh, Liudmilla Rubbi, Michael E. Phelps, Markus Mschen, Yong-Mi Kim, Arion F. Chatziioannou, Hsian-Rong Tseng, and Thomas G. Graeber. Integrated Microfluidic and Imaging Platform for a Kinase Activity Radioassay to Analyze Minute Patient Cancer Samples. *Cancer Research*, 70(21):8299–8308, November 2010.

[6] William H Grover, Alison M Skelley, Chung N Liu, Eric T Lagally, and Richard A Mathies. Monolithic membrane valves and diaphragm pumps for practical large-scale integration into glass microfluidic devices. *Sensors and Actuators B: Chemical*, 89(3):315–323, April 2003.

[7] Haiyao Huang. *Fluigi: An end-to-end Software Workflow for Microfluidic Design*. PhD thesis, Boston University, 2015.

[8] Haiyao Huang and Douglas Densmore. Fluigi: Microfluidic Device Synthesis for Synthetic Biology. *J. Emerg. Technol. Comput. Syst.*, 11(3):26:1–26:19, December 2014.

[9] Jungkyu Kim, Michael Johnson, Parker Hill, and Bruce K. Gale. Microfluidic sample preparation: cell lysis and nucleic acidpurification. *Integrative Biology*, 1(10):574–586, September 2009.

[10] Juta Kobayashi, Yuichiro Mori, Kuniaki Okamoto, Ryo Akiyama, Masaharu Ueno, Takehiko Kitamori, and Sh Kobayashi. A Microfluidic Device for Conducting Gas-Liquid-Solid Hydrogenation Reactions. *Science*, 304(5675):1305–1308, May 2004.

[11] Ali Lashkaripour, Ali Abouei Mehrizi, Mohamadreza Rasouli, and Masoud Goharimanesh. Numerical Study of Droplet Generation Process in a Microfluidic Flow Focusing. *Journal of Computational Applied Mechanics*, 46(2):167–175, July 2015.

[12] Ali Lashkaripour, Ryan Silva, and Douglas Densmore. Desktop micromilled microfluidics. *Microfluidics and Nanofluidics*, 22(3):31, February 2018.

[13] Cheng-Chung Lee, Thomas M Snyder, and Stephen R Quake. A microfluidic oligonucleotide synthesizer. *Nucleic acids research*, 38(8):2514–2521, 2010.

[14] Y. C. Lim, A. Z. Kouzani, and W. Duan. Lab-on-a-chip: a component view. *Microsystem Technologies*, 16(12):1995–2015, December 2010.

[15] Wenming Liu, Li Li, Xuming Wang, Li Ren, Xueqin Wang, Jianchun Wang, Qin Tu, Xiaowen Huang, and Jinyi Wang. An integrated microfluidic system for studying cell-microenvironmental interactions versatilely and dynamically. *Lab on a Chip*, 10(13):1717–1724, July 2010.

[16] Transon V. Nguyen, Philip N. Duncan, Siavash Ahrar, and Elliot E. Hui. Semi-autonomous liquid handling via on-chip pneumatic digital logic. *Lab on a Chip*, 12(20):3991–3994, September 2012.

[17] Kwang W. Oh, Kangsun Lee, Byungwook Ahn, and Edward P. Furlani. Design of pressure-driven microfluidic networks using electric circuit analogy. *Lab on a Chip*, 12(3):515–545, January 2012.

[18] Arthur Prindle, Phillip Samayoa, Ivan Razinkov, Tal Danino, Lev S. Tsimring, and Jeff Hasty. A sensing array of radically coupled genetic /'biopixels/'. *Nature*, 481(7379):39–44, January 2012.

[19] R. Silva, P. Dow, R. Dubay, C. Lissandrello, J. Holder, D. Densmore, and J. Fiering. Rapid prototyping and parametric optimization of plastic acoustofluidic devices for blood–bacteria separation. *Biomedical Microdevices*, 19(3):70, Aug 2017.

[20] Ryan Silva. *Makerfluidics: Low Cost Microfluidics for Synthetic Biology*. PhD thesis, Boston University, 2017.

[21] Ryan Silva, Swapnil Bhatia, and Douglas Densmore. A reconfigurable continuous-flow fluidic routing fabric using a modular, scalable primitive. *Lab on a Chip*, 16(14):2730–2741, July 2016.

[22] Todd M. Squires and Stephen R. Quake. Microfluidics: Fluid physics at the nanoliter scale. *Reviews of Modern Physics*, 77(3):977–1026, October 2005.

[23] Shia-Yen Teh, Robert Lin, Lung-Hsin Hung, and Abraham P. Lee. Droplet microfluidics. *Lab on a Chip*, 8(2):198–220, January 2008.

[24] Todd Thorsen, Sebastian J. Maerkl, and Stephen R. Quake. Microfluidic Large-Scale Integration. *Science*, 298(5593):580–584, October 2002.

[25] Todd Thorsen, Richard W. Roberts, Frances H. Arnold, and Stephen R. Quake. Dynamic Pattern Formation in a Vesicle-Generating Microfluidic Device. *Physical Review Letters*, 86(18):4163–4166, April 2001.

[26] John Paul Urbanski, William Thies, Christopher Rhodes, Saman Amarasinghe, and Todd Thorsen. Digital microfluidics using soft lithography. *Lab on a Chip*, 6(1):96–104, December 2006.

[27] C. Joanne Wang, Adriel Bergmann, Benjamin Lin, Kyuri Kim, and Andre Levchenko. Diverse Sensitivity Thresholds in Dynamic Signaling Responses by Social Amoebae. *Sci. Signal.*, 5(213):ra17–ra17, February 2012.

[28] Aaron R. Wheeler, William R. Throndset, Rebecca J. Whelan, Andrew M. Leach, Richard N. Zare, Yish Hann Liao, Kevin Farrell, Ian D. Manger, and Antoine Daridon. Microfluidic Device for Single-Cell Analysis. *Analytical Chemistry*, 75(14):3581–3586, July 2003.
